# No evidence for local adaptation and an epigenetic underpinning in native and non‐native ruderal plant species in Germany

**DOI:** 10.1002/ece3.5325

**Published:** 2019-08-06

**Authors:** Jasmin Herden, Silvia Eckert, Marc Stift, Jasmin Joshi, Mark van Kleunen

**Affiliations:** ^1^ Ecology, Department of Biology University of Konstanz Konstanz Germany; ^2^ Biodiversity Research/Systematic Botany, Institute of Biochemistry and Biology University of Potsdam Potsdam Germany; ^3^ Berlin‐Brandenburg Institute of Advanced Biodiversity Research (BBIB), Institute of Biology Freie Universität Berlin Berlin Germany; ^4^ Institute for Landscape and Open Space Hochschule für Technik Rapperswil (HSR) Rapperswil Switzerland; ^5^ Zhejiang Provincial Key Laboratory of Plant Evolutionary Ecology and Conservation Taizhou University Taizhou China

**Keywords:** biological invasions, epigenetics, local adaptation, reciprocal transplant experiment, ruderal plant species, zebularine

## Abstract

1. Many invasive species have rapidly adapted to different environments in their new ranges. This is surprising, as colonization is usually associated with reduced genetic variation. Heritable phenotypic variation with an epigenetic basis may explain this paradox.
2. Here, we assessed the contribution of DNA methylation to local adaptation in native and naturalized non‐native ruderal plant species in Germany. We reciprocally transplanted offspring from natural populations of seven native and five non‐native plant species between the Konstanz region in the south and the Potsdam region in the north of Germany. Before the transplant, half of the seeds were treated with the demethylation agent zebularine. We recorded survival, flowering probability, and biomass production as fitness estimates.
3. Contrary to our expectations, we found little evidence for local adaptation, both among the native and among the non‐native plant species. Zebularine treatment had mostly negative effects on overall plant performance, regardless of whether plants were local or not, and regardless of whether they were native or non‐native.
4. *Synthesis*. We conclude that local adaptation, at least at the scale of our study, plays no major role in the success of non‐native and native ruderal plants. Consequently, we found no evidence yet for an epigenetic basis of local adaptation.

## 1 INTRODUCTION

Over the last centuries, human activities have led to the introduction of thousands of plant species across biogeographical barriers (van Kleunen et al., 2018). Of these, more than 13,000 have become naturalized (van Kleunen et al., 2015; Pyšek et al., 2017), and, occasionally, such naturalized species become invasive with negative ecological and socioeconomic impacts (Simberloff et al., 2013; Vilà et al., 2011; Vilà & Hulme, 2017). Understanding how invasive species cope with the abiotic and biotic environment in their new range is therefore both of fundamental and applied interest (Allendorf & Lundquist, 2003; Estoup et al., 2016; Schrieber & Lachmuth, 2017).

The adaptability of invasive species is surprising, since many non‐native species go through genetic bottlenecks during introduction, which is likely to reduce genetic variation (Dlugosch & Parker, 2008; Hollingsworth & Bailey, 2000; Schrey et al., 2012; Zhang, Zhang, & Barrett, 2010). Nevertheless, there is evidence from comparisons between native and introduced populations that some invasive species have rapidly adapted to new environments (Joshi & Vrieling, 2005; Zhang et al., 2018). Moreover, common‐garden studies revealed that trait expression of naturalized non‐native plants often appears to follow altitudinal, climatic, or latitudinal clines (Agrawal et al., 2005; Alexander, Kleunen, Ghezzi, & Edwards, 2012; Bhattarai et al., 2017; Kollmann & Bañuelos, 2004; Weber & Schmid, 1998; but see, e.g., Colautti & Lau, 2015; Datta, Kühn, Ahmad, Michalski, & Auge, 2017; Ebeling, Stöcklin, Hensen, & Auge, 2011). Such clines imply that local populations of non‐native species have been subject to divergent selection. Indeed, a number of common‐garden and reciprocal transplant studies have found evidence for local adaptation in non‐native species within their introduced range (Colautti & Barrett, 2013; Maron, Vilá, Bommarco, Elmendorf, & Beardsley, 2004; Oduor, Leimu, & Kleunen, 2016). However, it remains unknown whether such patterns of rapid adaptation within the introduced ranges of invasive species are very common and whether they are achieved by genetic change alone.

Local adaptation could in theory also have an epigenetic basis (Bossdorf, Richards, & Pigliucci, 2008; Hawes et al., 2018), and this might be particularly important in the absence of genetic variation. Local adaptation through epigenetic modification might involve gene regulation via micro‐RNAs, small interfering RNAs, histone modifications, or cytosine methylation (hereafter, DNA methylation; Henderson & Jacobsen, 2007; Nicotra et al., 2010; Rapp & Wendel, 2005). Of those different epigenetic mechanisms, DNA methylation is the most widely studied (Hawes et al., 2018; Kilvitis et al., 2014; Schrey et al., 2013). In plants, DNA methylation can occur at different sequence positions of cytosines (i.e. ^m^CG, ^m^CHG or ^m^CHH; ^m^C—5‐methyl‐cytosine, G—guanine, H—any other DNA base except guanine; van der Graaf et al., 2015) and is under control of a suite of cellular maintenance mechanisms (Kawashima & Berger, 2014; Niederhuth & Schmitz, 2017). Loss and gain of DNA methylation at specific sites is thought to be spontaneous (Johannes & Schmitz, 2018; van der Graaf et al., 2015), and epimutation rates appear to exceed mutation rates (Johannes & Schmitz, 2018). Most importantly, in angiosperms, DNA methylation can be transmitted transgenerationally, through both asexual and sexual reproduction (Henderson & Jacobsen, 2007; Kawashima & Berger, 2014), and thus produce heritable phenotypes (Cubas, Vincent, & Coen, 1999; Manning et al., 2006; Niederhuth & Schmitz, 2014; Wilschut, Oplaat, Snoek, Kirschner, & Verhoeven, 2016). This implies that DNA methylation could be an epigenetic mechanism that allows for fast local adaptation.

Previous studies detected differentiated DNA methylation patterns across natural populations with the help of methylation‐sensitive molecular markers. DNA methylation patterns have been linked to specific habitats of native and non‐native plant species (Lira‐Medeiros et al., 2010; Platt, Gugger, Pellegrini, & Sork, 2015; Richards, Schrey, & Pigliucci, 2012), disturbance (Herrera & Bazaga, 2016), and environmental stress (Herrera & Bazaga, 2011; Kooke et al., 2015; Robertson, Schrey, Shayter, Moss, & Richards, 2017). However, while these studies provide evidence for epigenetic differentiation, they cannot infer whether the observed patterns reflect local adaptation. Therefore, the next logical step would be for studies to experimentally modify DNA methylation in plants before testing their fitness under field conditions. However, to the best of our knowledge, such studies have not been done yet.

Here, we tested in a regional reciprocal transplant experiment whether treatment with the demethylation agent zebularine affects local adaptation in native and non‐native ruderal plant species. Zebularine works as an inhibitor to DNA methyltransferases (Baubec, Pecinka, Rozhon, & Mittelsten Scheid, 2009; Griffin, Niederhuth, & Schmitz, 2016; Marquez, Barchi, et al., 2005; Marquez, Kelley, et al., 2005), which are an important part of the cellular maintenance mechanisms for DNA methylation (Baubec et al., 2009; Niederhuth & Schmitz, 2014). Importantly, zebularine does not induce genetic mutations (Bossdorf et al., 2008). Zebularine treatment during germination and the seedling stage in *Arabidopsis thaliana* was shown to result in hypomethylation of cytosine residues at all sites (e.g. reduction of total DNA methylation from 81.4% in untreated to 58.8% in treated plants after 80 µM zebularine; Baubec et al., 2009). This hypomethylation has the potential to erase transgenerationally transmitted methylation states conferring improved responses to drought (Herman, Sultan, Horgan‐Kobelski, & Riggs, 2012), herbivory, and salt stress (Verhoeven, Van Dijk, & Biere, 2010).

We expected that epigenetic inheritance would contribute more to local adaptation in naturalized non‐native species than in native species. This is because in contrast to native species, non‐native species may have less genetic variation, as a consequence of genetic bottlenecks during introduction (Dlugosch & Parker, 2008), and have had less time, due to their recent introduction, to allow for local adaptation by genetic mechanisms.

To address this, we collected seeds from multiple maternal lines of seven native and five non‐native short‐lived ruderal species from two climatically and latitudinally different regions in Germany: the Konstanz region in southern Germany and the Potsdam region, situated c. 600 km to the northeast of Konstanz. Half of the seeds of each maternal line were treated with zebularine during germination. We then planted the zebularine‐ and non‐zebularine‐treated offspring from these two regions into three field sites in the Potsdam region and three field sites in the Konstanz region. We recorded survival, flowering probability, aboveground biomass, and reproductive biomass as fitness‐related traits.

We asked three specific questions: (a) Do local plants outperform nonlocal plants of the same species (i.e., is there local adaptation *sensu* Kawecki & Ebert, 2004)? If local plants show higher survival or flowering, or produced more biomass than nonlocal plants in transplant sites of both regions, this would indicate local adaptation. Based on previous meta‐analyses of local adaptation in plants (Leimu & Fischer, 2008; Oduor et al., 2016), we expected to find evidence for local adaptation in most study species. (b) Does the degree of local adaptation differ between native and non‐native species? We expected local adaptation of similar strength and frequency in native and non‐native species, in line with the meta‐analysis results of Oduor et al. (2016). (c) Do zebularine‐treated plants show less evidence for local adaptation than control plants, and is this effect stronger for non‐native than for native plant species? We expected local plants to outperform nonlocal plants under control conditions, but that zebularine treatment would weaken or remove this effect, especially in non‐native plants. Such a finding would indicate that DNA methylations are a mechanism underlying local adaptation, particularly in non‐native species.

## 2 MATERIALS AND METHODS

### 2.1 Species selection and seed collection

As study species for the reciprocal transplant experiment, we chose native and non‐native species that are common throughout Germany and occur in the Konstanz (47.6779°N, 9.1732°E) and Potsdam (52.3906°N, 13.0645°E) regions according to the FloraWeb database (www.floraweb.de, Bundesamt für Naturschutz). To facilitate approximation of lifetime fitness, and facilitate interspecific comparisons, we specifically targeted short‐lived (mainly annual) species from similar ruderal habitats. This habitat type was selected, because ruderal sites such as agricultural fields and fallow land in urban areas are especially rich in naturalized neophytes (Chytrý, Jarošík et al., 2008; Chytrý, Maskell et al., 2008), and the ruderal strategy is widely shared among naturalized non‐native plants (Baker, 1974; Guo et al., 2018). To avoid confounding floristic status with taxonomy, we selected multiple confamilial groups that each contained at least one native and one naturalized non‐native species. Using these criteria, we managed to collect viable seeds within a radius of 50 km around Konstanz and Potsdam for seven native and five naturalized non‐native species, representing four families (Amaranthaceae, Asteraceae, Plantaginaceae, and Solanaceae; Table 1; species determined with Senghas & Seybold, 1993 and Jäger et al., 2013). Seeds were collected from July to November 2015, and we aimed to collect seeds from at least 10 plants (maternal lines) per population. (See Table S1 for species, number of maternal lines and sampling locations, and Table S19 for native range and invasion history of non‐native species.) Seeds were stored at room temperature in paper bags until sowing.

**Table 1:** The 12 ruderal study species used in our reciprocal transplant experiment between the Konstanz and Potsdam regions of Germany. Standardized species names were obtained from The Plant List (http://www.theplantlist.org/)

Family	Species	Status^a^	Growth form^b^	Life form^b^
Amaranthaceae	*Amaranthus retroflexus* L.	Non‐native	Annual	Therophyte
	*Chenopodium album* L.	Native	Annual	Therophyte
Asteraceae	*Erigeron canadensis* L.	Non‐native	Annual	Therophyte/hemicryptophyte
	*Erigeron annuus* (L.) Pers.	Non‐native	Biennial	Hemicryptophyte
	*Lactuca serriola* L.	Native	Annual	Therophyte/hemicryptophyte
	*Senecio vulgaris* L.	Native	Annual	therophyte/hemicryptophyte
	*Sonchus oleraceus* (L.) L.	Native	Annual	Therophyte/hemicryptophyte
	*Tripleurospermum inodorum* (L.) Sch.Bip.	Native	Annual	Therophyte/hemicryptophyte
Plantaginaceae	*Veronica persica* Poir.	Non‐native	Annual	Therophyte/hemicryptophyte
	*Plantago major* L.	Native	Perennial (plurienn‐pollakanth)	Hemicryptophyte
Solanaceae	*Datura stramonium* L.	Non‐native	Annual	Therophyte
	*Solanum nigrum* L.	Native	Annual	Therophyte

a Data on the native status of species were obtained from FloraWeb (Bundesamt für Naturschutz).

b Data on growth form and life form were obtained from the BiolFlor database (Kühn, Durka, & Klotz, 2004).

### 2.2 Pre‐cultivation of study species and zebularine treatment

Before transplant into the common‐garden field sites, we pre‐cultivation plants in the botanical gardens of the University of Konstanz (for the Konstanz region) and the University of Potsdam (for the Potsdam region) during the second half of April and the first half of May 2016. For some species, the seeds were scarified with H_2_SO_4_ or soaked in water before sowing to promote germination (Table S2). Immediately before sowing, all seeds were surface sterilized in 5% NaClO for 3 min and then rinsed three times in deionized water. To assure that all plants would be at a viable size at the start of the experiment, the sowing dates of species were adjusted to known germination speed (see Table S2 for details).

For each of the maternal seed lines (see Table S1 for the number of maternal lines used per species), we prepared two plastic petri dishes (diameter: 35 mm) with filter paper on the bottom. For the control treatment, the filter paper was moistened with 200 µl of deionized water, and for the demethylation treatment, it was moistened with 200 µl of a 35 µM aqueous solution of the demethylation agent zebularine (Sigma‐Aldrich Corporation, St. Louis, Missouri, USA). The used concentration of zebularine, C_9_H_12_N_2_O_5_, a cytidine analogue, was chosen to be within the range of concentrations used by other studies, where they were shown to be effective without affecting plant survival (see Alonso, Medrano, Pérez, Bazaga, & Herrera, 2017; Verhoeven & van Gurp, 2012). Moreover, in a pilot study, we found that a concentration between 25 and 50 µM zebularine visibly slowed plant development, without affecting the viability of the plants (see Figure S1 for images of exemplary gradients of the zebularine trial). Depending on seed availability and size, we put 10–20 seeds in each petri dish. In total, we had 765 petri dishes in Konstanz and 768 petri dishes in Potsdam.

To prevent the seeds from drying out, we sealed the petri dishes with parafilm. Then, the petri dishes were randomly assigned to positions in a phytochamber (11‐hr light at 21°C and 13‐hr dark at 16°C) and covered with a single layer of 80 g/m^2^ white paper to reduce condensation on the inside of the lids of the petri dishes. Although zebularine has a higher chemical stability than other methyltransferase inhibitors (Cheng et al., 2003; Zhou et al., 2002), in an aqueous solution, it degrades within a few days (Marquez, Barchi, et al., 2005; Marquez, Kelley, et al., 2005). Therefore, every second day, we transferred the seeds to new petri dishes with a freshly prepared zebularine solution or, in the case of the control treatment, with fresh water, until at least three seedlings had germinated.

For each of the 12 species, we transplanted all seedlings as soon as there were at least three seedlings in the majority of petri dishes of that respective species. For petri dishes that had fewer than three seedlings at that point (up to 8% of petri dishes within a species), we transplanted all available seedlings, resealed the petri dishes, and continued transferring remaining seeds to fresh dishes. We did this until three seedlings had germinated or until the 8 May 2016 (in Konstanz) or the 13 May 2016 (in Potsdam) (see Table S3 for the transplanting timeline).

We transplanted the seedlings to 7×7×6.5 cm pots filled with a peat‐based substrate (Pikiererde Classic CL P, Einheitserdewerke Patzer). For each petri dish (i.e. maternal line by zebularine treatment combination), up to three pots were prepared. When there were more than three seedlings available, we planted up to three seedlings in a single pot, to increase the chance that at least one of them would survive until transplanting in the field sites. The pots were randomly allocated to positions in a glasshouse. At least 1 week before planting at the field sites, plants were placed outside in a sun‐protected place for acclimatization to field conditions.

### 2.3 Field sites and experimental set‐up

Seeds of the 12 study species had been collected in different locations in the Konstanz and Potsdam regions (Table S1). As it was logistically not possible to reciprocally transplant the offspring of species between the exact locations were the seeds had been collected, we instead established three experimental field sites in Konstanz and three experimental field sites in Potsdam, where we planted all 12 species. These sites were agricultural fields or tilled grasslands (i.e. disturbed to mimic ruderal sites; see Table S4 for exact descriptions of the field sites).

Each field site was at least 100 m^2^ and was divided into three blocks. Following a randomized block design per field site, we randomly allocated one‐third of the maternal lines of each species to each block. For each maternal line, we planted, if possible, one control individual and one zebularine‐treated individual into each of the three Konstanz and each of the three Potsdam field sites. To avoid interspecific competition, each block of a field site was subdivided into 12 plots, that is, one for each species. To avoid intraspecific competition within plots, we planted individual plants 30 cm apart in a 7×4 grid (1.7 m^2^; see Figure S2 for an example), except for the larger *Datura stramonium*, which was planted 50 cm apart in a 5×3 grid in plots of 3.0 m^2^. Although we aimed to have all maternal lines of each species represented with a control plant and a zebularine‐treated plant in all six field sites, this was not possible for all maternal lines due to insufficient germination or survival of seedlings. In such cases, the number of complete treatment level pairs per maternal line was maximized, and these pairs were randomly assigned to field sites in each region (Konstanz, Potsdam). Leftover single plants of these maternal lines were randomly assigned to the remaining field sites.

Plants were transplanted into the three Konstanz field sites from 17 to 25 May 2016 (i.e. 4–5 weeks after sowing) and into the three Potsdam field sites from 5 to 13 June 2016 (i.e. 7–8 weeks after sowing). To avoid damaging the root systems during transplant, we did not remove the potting soil from the plants before planting. As some pots had up to three small individuals in a pot, we kept the largest individual and removed the others. Plants were watered twice a week during the first 2 weeks after transplanting, to reduce mortality and facilitate establishment. Additionally, because the summer of 2016 was unusually dry in Potsdam, we watered the plants there once or twice a week during episodes of severe drought (all field sites from the beginning of June to mid‐July and the Gröben field site from mid‐August to the end of September 2016). At the Konstanz field sites, we reduced mortality due to mollusk herbivory by sprinkling a molluscicide (Schneckenkorn Spiess‐Urania®G2, Spiess‐Urania Chemicals GmbH, Hamburg, Germany) around the fields at the start of the experiment and at least once more during July–August 2016. At the Potsdam field sites, however, molluscicides were not required as slug and snail numbers there were low (Silvia Eckert, personal observation), probably due to the sandy soil and the unusually dry summer in 2016. We did not weed the plots, unless there was potential for confusion with experimental plants belonging to the same species.

### 2.4 Harvest and measurements

In the weeks before harvesting, we scored for each plant whether it flowered (or had flowered). We harvested all plants of a species in a specific field site as soon as at least 50% of all surviving plants had started to flower, and the first seeds were mature. In cases where seeds formed before 50% of the plants flowered (*Erigeron annuus*, *Erigeron canadensis*, and *Lactuca serriola*), we collected mature reproductive units from flowering plants to avoid losing reproductive biomass. At the end of the growing season (end of October 2016), we harvested all remaining plants on all field sites, regardless of the percentage of flowering plants. At harvest, we collected the aboveground biomass and separated it into reproductive and vegetative parts. Biomass was dried for at least 72 hr at 70°C in a drying oven and then weighed.

### 2.5 Statistical analysis

The final data set used for analysis comprised 3,864 plant individuals, 2,068 from the Konstanz field sites and 1,796 from the Potsdam field sites. As measures of plant fitness or performance, we used survival, flowering probability, aboveground biomass, and reproductive biomass. From the analyses of flowering probability and reproductive biomass, we excluded 33 plants that had started flowering before planting in the Potsdam field sites (9 out of 97 *D. stramonium* plants, 5 out of 94 *Plantago major* plants, and 19 out of 114 *Senecio vulgaris* plants). Survival was analyzed for all plants (*n* = 3,729). Total aboveground biomass (*n* = 2,951) and flowering probability (*n* = 2,956) were analyzed for the surviving plants, and reproductive biomass was only analyzed for flowering plants (*n* = 2,293). We used a meta‐analytical approach, which facilitates comparisons across species and field sites, to analyze effect sizes of differences between local and nonlocal plants. For explorative purposes, we also analyzed each species separately to test for effects of transplant region, zebularine treatment, and origin (see Methods S1).

We used a meta‐analytical approach to test (a) whether there was a general signature of local adaptation across all study species (see also Leimu & Fischer, 2008, Oduor et al., 2016), (b) whether this signature differed between native and naturalized non‐native species, and (c) whether zebularine treatment had an effect on local adaptation. To fulfill the requirements for local adaptation, local populations in both tested regions must outperform the nonlocal populations (Kawecki & Ebert, 2004). We calculated effect sizes for the meta‐regressions such that positive values corresponded to a higher performance of the local populations (and negative values corresponded to a higher performance of nonlocal populations). Therefore, positive effect sizes in both regions would indicate local adaptation, whereas negative effect sizes would indicate local maladaptation (Leimu & Fischer, 2008).

All statistical analyses were done with R (version R‐3.4.1; R Core Team, 2017) using RStudio (version 1.0.153; RStudio Team, 2015). We used the “escalc” function in the metafor R package (Viechtbauer, 2010) to calculate effect sizes separately by species and zebularine treatment level. Effect sizes for the two continuous variables, total aboveground biomass and reproductive biomass, were calculated separately for each of the three blocks of a field site. For these two biomass variables, we calculated the effect sizes as standardized mean differences (SMDs) between the local and the nonlocal populations (Borenstein, Hedges, Higgins, & Rothstein, 2009; Leimu & Fischer, 2008; Viechtbauer, 2010, 2016). Effect sizes for the two binomial variables, survival and flowering probability, were calculated separately for each field site (i.e. across the three blocks of a field site). For these two binomial variables, we calculated effect sizes as log‐transformed odds ratios (LORs) from 2×2 contingency tables (Borenstein et al., 2009; Viechtbauer, 2010). We accounted for zeroes in the 2×2 contingency tables by using the default continuity correction of 0.5 in the metafor package (Viechtbauer, 2010). However, we also analyzed these data using an alternative continuity correction that is based on the ratio of sample sizes between the compared groups (Sweeting, Sutton, & Lambert, 2004; see Methods S3 for more details). For the effect sizes (SMDs and LORs), we also calculated the corresponding variances (Borenstein et al., 2009; Viechtbauer, 2010, 2016). For the visualization of effect sizes in forest plots, effect sizes were summarized by species and zebularine treatments using the “rma.mv” function in the metafor R package (Viechtbauer, 2010, 2016). As random effects, we used fields and blocks (nested within field) for summarizing within regions (Figure S4 and Tables S9–S12), and for summarizing across regions (Tables S13–S16), we used region, field sites (nested within region), and blocks (nested within field sites). A significant effect size would have 95% confidence intervals not overlapping with zero.

**Figure 1:**
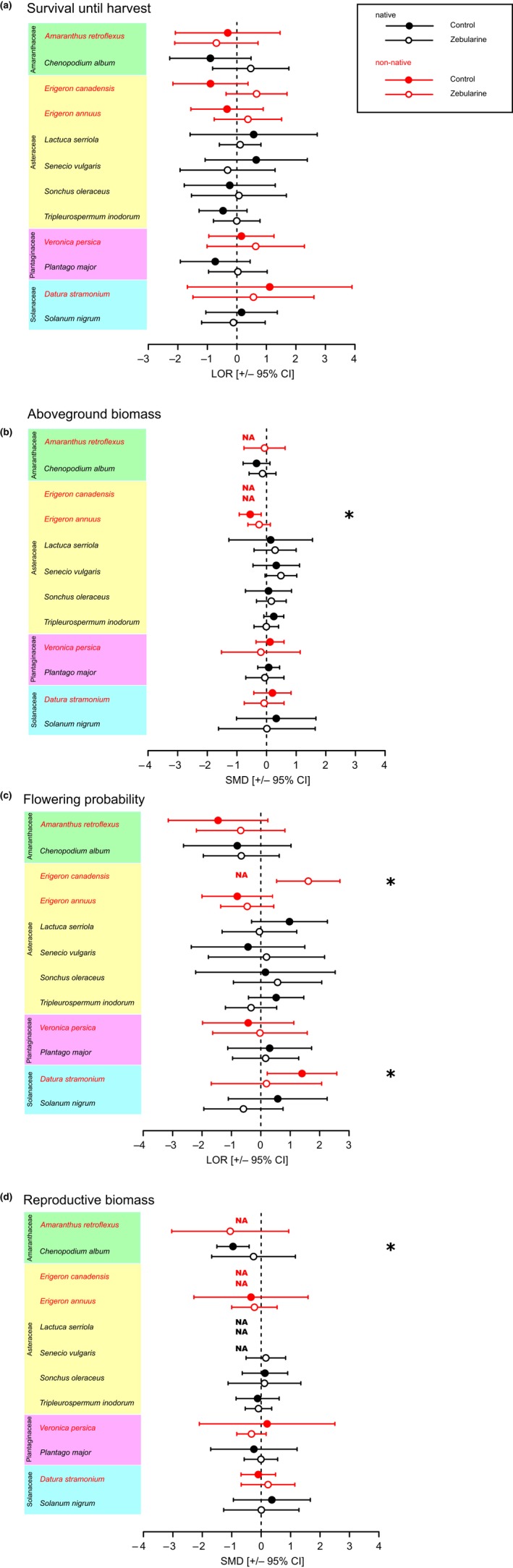
Forest plots with effect sizes summarized across regions. Effect sizes were calculated as the difference between local and nonlocal plants. Significantly positive across‐region effect sizes indicate local adaptation and negative ones indicate local maladaptation. Stars denote effect sizes significantly different from 0 (i.e. 95% confidence intervals nonoverlapping with 0). NAs denote cases with insufficient data for effect size calculation in one or both regions (see Methods S2). Closed and open symbols stand for control and zebularine treatment, respectively. Natives are marked in black, and non‐natives are marked in red. Survival with continuity correction based on the reciprocal of the opposite group size (a), aboveground biomass (b), flowering probability based on the reciprocal of the opposite group size (c), and reproductive biomass (d). LOR, log‐transformed odds ratio; SMD, standardized mean difference

To test whether effect sizes were significantly affected by the native versus non‐native status, and whether effect sizes significantly changed due to the zebularine treatment, we analyzed effect sizes of each fitness or performance variable (survival, aboveground biomass, flowering probability, and reproductive biomass) separately in mixed‐effects meta‐regression models with the “rma.mv” function. The models included region of the field site (Konstanz vs. Potsdam), floristic status of the species (native vs. non‐native), and zebularine treatment (untreated vs. treated) as two‐level factorial moderators, and their interactions. In addition, the models included field site, block nested within field site, plant family, and species nested within plant family as random effects. We aimed to use the full model whenever possible. However, in some cases, the full model did not converge, or profile likelihood plots indicated overparameterization. In such cases, we removed one or both of the outer random factors (i.e., plant family and field site) to get a converging model that was not overparameterized. Plots of distribution of the residuals, residuals versus fitted values, and qqplots indicated that the assumptions of the analysis were not violated. We obtained likelihood‐ratio‐test statistics and corresponding p‐values for moderators and their interactions by step‐wise model reduction. Finally, to test for the global effect of a fitness variable, we also analyzed effect sizes with meta‐regression models with the “rma.mv” function without moderators, but the full set of random effects (unless there were problems with convergence or overparameterization of the models.

**Table 2:** Results of meta‐regression for each fitness variable, without moderators, and random effects of blocks nested in field sites and species nested within plant family. The values are (in order) the continuity correction (CC) applied to the effect sizes of the respective model, the global effect size estimate of the model, the standard error (*SE*), and the corresponding *Z‐* and *p*‐values. Sample sizes of effect sizes were the same as specified for the respective fitness variable in mixed‐effects meta‐regressions (Table 3)

Fitness variable	Continuity correction (CC)	Effect size estimate ± SE	Z	*p*	Random effects structure
Survival	+0.5	0.02 ± 0.11	0.164	0.870	~1|Field
	+ local_CC_ +non‐local_CC_	0.03 ± 0.11	0.325	0.745	~1|Species
Aboveground biomass	NA	0.03 ± 0.09	0.307	0.759	~Block|Field
					~1|Species
Flowering probability	+0.5	0.01 ± 0.21	0.034	0.973	~1|Field
	+ localCC +non‐localCC	0.07 ± 0.19	0.358	0.720	~1|Species
Reproductive biomass	NA	−0.22 ± 0.24	−0.916	0.360	~Block|Field
					~Species|Plant family

**Table 3:** Results of mixed effects meta‐regression for each fitness variable, with transplant region (Konstanz or Potsdam), status (native or non‐native), zebularine treatment and their interactions as moderators and random effects of blocks nested in field sites and species nested within plant family. The values are for each moderator in the step‐wise model reduction, the χ2 value and the significance level of the likelihood ratio test. *p*‐values lower than 0.05 are marked in bold. For survival and flowering probability, results for both types of continuity correction are shown (for details see Methods S3)

Fitness variable	Survival		Aboveground biomass	Flowering probability		Reproductive biomass
Sample size	91	143	300	100	134	215
Effect size	LOR	LOR	SMD	LOR	LOR	SMD
Comparison level	Field	Field	Block	Field	Field	Block
Continuity correction	+0.5	+ local_CC_ +non‐local_CC_	NA	+0.5	+local_CC_ +non‐local_CC_	NA

Moderators	χ^2^ (*df* = 1)	*p*	χ^2^ (*df* = 1)	*p*	χ^2^ (*df* = 1)	*p*	χ^2^ (*df* = 1)	*p*	χ^2^ (*df* = 1)	*p*	χ^2^ (*df* = 1)	*p*
Region (R)	1.90	0.168	1.23	0.267	2.13	0.144	1.02	0.312	0.06	0.816	**8.68**	**0.003**
Status (S)	1.89	0.170	1.58	0.209	2.34	0.126	0.06	0.800	0.03	0.861	3.65	0.056
Zebularine treatment (Z)	2.22	0.136	1.65	0.199	0.02	0.882	0.00	0.952	0.01	0.929	0.00	0.963
R × S	1.37	0.241	1.18	0.277	**8.66**	**0.003**	0.84	0.359	0.50	0.479	0.35	0.552
R × Z	0.93	0.336	0.70	0.402	0.31	0.578	0.10	0.756	0.32	0.572	0.33	0.563
S × Z	0.00	0.986	0.00	0.944	0.95	0.331	2.31	0.129	2.43	0.119	0.54	0.462
R × S × Z	0.09	0.770	0.13	0.719	**4.39**	**0.036**	3.60	0.058	2.69	0.101	1.01	0.314
Random effects structure	~1|Field		~1|Field		~Block|Field		~1|Field		~1|Field		~Block|Field	
	~1|Species		~1|Species		~1|Species		~1|Species		~1|Species		~Species|Plant family	

Note

Abbreviations: LOR, log‐transformed odds ratio; SMD, standardized mean difference.

## 3 RESULTS

Overall, survival (80.3%) and flowering probability (78.4%) were high and most plants set seeds during the experiment. Survival ranged from 42.9% for the native *L. serriola* to 97.9% for the native *Senecio vulgaris* (Table S5). Flowering percentages ranged from 11.1% for the native *L. serriola* to 97.4% for the native *S. nigrum* (Table S7). Plants generally produced more biomass in the field sites of the Potsdam region than in the field sites of the Konstanz region (i.e. in three out of 12 species for aboveground biomass and in six out of 12 species in reproductive biomass) (Tables S6 and S8, Figures S5–S16). Only *Plantago major* produced more biomass in the Konstanz than in the Potsdam region (Tables S6 and S8, Figure S14). However, survival (Table S5) and flowering (Table S7) did not significantly differ between the Konstanz and Potsdam transplant regions.

### 3.1 Overall evidence for local adaptation of the study species?

With global effect sizes (i.e., effect sizes averaged across all study species) not significantly different from zero for any of the performance traits, meta‐regressiation revealed no evidence for local adaptation (Table 2). In other words, local and nonlocal plants performed similarly.

However, when effect sizes were summarized across both transplant regions for each individual species and treatment, we found a few significant effect sizes in the control treatment (Figure 1). One of those was a positive effect size for flowering probability in the non‐native *D. stramonium* (Figure 1c), indicating superior performance of local plants in both regions. On the other hand, there were significantly negative effect sizes for aboveground biomass in the non‐native *E. annuus* (Figure 1b) and for reproductive biomass in the native *Ch. album* (Figure 1d), indicating superior performance of nonlocal plants in both regions. Details on effect sizes of species in each of the two regions are provided in the Notes S2, Figure S4, and Tables S9–S12, and the results of single‐species analyses are provided in Notes S1, Tables S5–S8, and Figures S5–S16. Overall, both the meta‐analytical approaches and the single‐species analyses provide only scant evidence for local adaptation, but more evidence for local maladaptation.

### 3.2 Does the degree of local adaptation differ between native and non‐native species?

There was no evidence for differences in local adaptation between native and non‐native species (no significant effects of status in the meta‐regression models in Table 3). However, in the meta‐regression model for aboveground biomass, the region:status and region:status:zebularine treatment interactions were significant (Table 3). This reflects that in the Konstanz field sites all predicted effect sizes were close to zero, whereas in the Potsdam field sites the predicted effect size of the zebularine‐treated plants was positive for the natives and negative for the non‐natives (Figure S3c).

Furthermore, in the meta‐regression for reproductive biomass, the moderator region explained effect sizes significantly and the status had a marginally significant effect (Table 3). This reflects that in the Konstanz field sites the predicted effect sizes tended to be negative and that predicted effect sizes overall tended to be lower for non‐native than for native species (Figure S3f). Thus, overall, the meta‐analytical approach provides scant evidence for the importance of status (native vs. non‐native) for the expression of local adaptation or maladaptation.

### 3.3 Effect of zebularine on local adaptation in natives and non‐natives?

The single‐species analyses allowed us to test the direct effect of zebularine on performance traits. These analyses showed that survival was completely unaffected by the zebularine treatment (Table S5) and that flowering was affected in only one species (Table S7, Figure S11). On the other hand, the zebularine treatment had significant negative effects on aboveground biomass production in seven of the 12 species and on reproductive biomass in seven of the 12 species (Tables S6 and S8, Figures S5–S16). However, we found significant positive effects of zebularine treatment on aboveground and reproductive biomass in the native *Ch. album* (see Tables S6 and S8, Figure S6). So, zebularine treatment had significant effects on plants, but the effects depended on the fitness trait and on the species.

None of the meta‐regression models for the four fitness variables revealed significant zebularine effects or status:zebularine interactions (Table 3). In other words, zebularine did not affect the magnitude of local adaptation, and this was the same for native and non‐native species.

However, when effect sizes were summarized across both transplant regions for each individual species and treatment, we found a few significant changes in the effect sizes due to zebularine treatment (Figure 1). In the non‐native *D. stramonium*, the significantly positive effect size for flowering in the control treatment disappeared in the zebularine treatment (Figure 1c). For the non‐native *E. canadensis*, the effect size for flowering was significantly positive in the zebularine treatment (Figure 1c). However, because the corresponding effect size in the control treatment could not be calculated due to high mortality of *E. canadensis* plants in Potsdam (Figure S7), it is not clear whether this reflects a change in effect size or not. If we compare the *E. canadensis* flowering effect sizes for the Konstanz transplant region only (Figure S4c), it appears that the effect size was larger for the zebularine‐treated than for the control plants (for details, see Notes S2 and Figure S4c). On the other hand, in the non‐native *E. annuus*, the significantly negative effect size for aboveground biomass, indicating local maladaptation, disappeared in the zebularine treatment (Figure 1b). Similarly, the significantly negative effect size for reproductive biomass in the native *Ch. album* also disappeared in the zebularine treatment (Figure 1d). (Details on effect sizes of native and non‐native species in each of the two regions are provided in the Notes S2, Figure S4, and Tables S9–S12, and the results of single‐species analyses are provided in Notes S1, Tables S5–S8, and Figures S5–S16.).

Overall, the analyses revealed hardly any evidence for the influence of zebularine treatment on the expression of local adaptation or maladaptation in natives or non‐natives.

## 4 DISCUSSION

Our multispecies reciprocal transplant experiment of five naturalized non‐native and seven native ruderal plant species between the Konstanz and Potsdam regions in Germany revealed no consistent differences in survival, growth, and reproduction between local and nonlocal plants. Treatment with the demethylation agent zebularine reduced performance (particularly biomass) of most species but showed no consistent effects on differences between local and nonlocal plants. So, our study revealed no clear evidence for local adaptation. Consequently, there were also no differences between native and non‐native species in this respect, and no evidence for the role of epigenetic mechanism, such as DNA methylation, in rapid adaptation of ruderal plants.

### 4.1 Local adaptation of ruderal plants

Although our results could be interpreted as evidence for local adaptation in individual species with regard to certain fitness components (e.g. in *D. stramonium* with regard to flowering probability), overall our study revealed little evidence for local adaptation across all 12 species. For several species in our study (e.g. in *Ch. album* and *E. annuus*), nonlocal plants even performed better than local plants (see Figure 1), suggesting local maladaptation. These findings are surprising given that several meta‐analyses revealed that local adaptation is quite common, though not ubiquitous (Hereford, 2009; Hoeksema & Forde, 2008; Leimu & Fischer, 2008; Oduor et al., 2016). For example, Leimu and Fischer (2008) found that local plants outperformed nonlocal plants in 71% of the study sites and that this happened at both sites of a reciprocally transplanted pair of populations in 45% of the cases. Leimu and Fischer (2008) found, however, more evidence for local adaptation when the populations were large (>1,000 individuals) than when they were small, possibly because of larger evolutionary potential and lower inbreeding and drift in large populations. The fact that most populations that we sampled were relatively small might partly explain the limited evidence for local adaptation in our study.

We used a multi‐species approach, which is powerful for detecting general patterns across species (van Kleunen, Dawson, Bossdorf, & Fischer, 2014). The results for the individual species should, however, be interpreted with caution, as our design merely included two populations for each of the 12 species (Table S1). Differences in performance between the two populations of a species, irrespective of whether the differences are in line with local adaptation or maladaptation, suggest that there is genetic (or epigenetic) differentiation (Tables S5–S8, Figures S5–S16). However, these differences could also have arisen due to random evolutionary processes, such as genetic drift, rather than due to adaptive evolution (Excoffier & Ray, 2008; Kawecki & Ebert, 2004). Furthermore, like in some previous studies on local adaptation (e.g. Colautti & Barrett, 2013), we could not transplant the species into the exact same locations where we had collected the seeds. Therefore, some field sites might by chance have been more similar to the collection locations of nonlocal seeds than to the collection locations of local seeds. So, even if local populations were adapted to the local conditions in the places where their seeds had been collected, they might not be adapted to the more regional environmental conditions of the field sites in their home region.

As ruderal plant species typically occur in recently disturbed but ephemeral open habitats, such as building sites and fallow land (Baker, 1974), they are likely to follow meta‐population dynamics (Bastin & Thomas, 1999; Schleicher, Biedermann, & Kleyer, 2011). Therefore, we expected our study species to be adapted to their regional climatic, edaphic, and biotic conditions (Bucharova et al., 2017; Keller, Kollmann, & Edwards, 2000), and thus, that plants from the Potsdam region would outperform plants from the Konstanz region in the Potsdam field sites and *vice versa*. Konstanz and Potsdam are more than 600 km apart, and whereas Konstanz has a warm climate to temperate oceanic climate, Potsdam has a rather temperate continental climate (Peel, Finlayson, & McMahon, 2007). So, generally, in Konstanz, climatic conditions are milder and wetter (also see Table S18). For instance, on average, Konstanz has a 33% higher mean annual precipitation, 16% fewer frost days, and a four degrees‐higher minimum temperature (Table S18). Furthermore, edaphic conditions clearly differed between regions (Table S17): The soil samples in the Konstanz field sites had on average a higher water content (22.3% vs. 6.6%), a higher potential pH value (7.3 vs. 5.6), and a higher organic matter content (6.7% vs. 3.9%) than soil samples from Potsdam field sites (cf. Table S17). This probably reflects more loamy soils in the Konstanz region and more sandy soils in the Potsdam region. Differences in performance of several of our study species between the Konstanz and Potsdam transplant regions (Figures S5–S16) further confirm the environmental differences between both regions. It is thus unlikely that the prevailing selective regimes between both regions were not sufficiently different to drive local adaptation.

Another explanation for the absence of local adaptation might be gene flow between northern and southern populations that is so high that local adaptation is impossible due to gene swamping (Kirkpatrick & Barton, 1997; Lenormand, 2002). This gene flow might also be partly facilitated by human impact, such as the transport of soil within the considered Central European range. Additionally, even though the non‐native study species have been introduced to both regions more than a century ago (see Table S19), humans might still continue to facilitate genetic exchange between the native and non‐native ranges, thus preventing local adaptation. Therefore, while we focused on ruderal species, because their short life cycle allows for better estimation of lifetime fitness, and because many successful non‐native species are ruderals (Guo et al., 2018; Kalusová et al., 2017), future studies should also consider nonruderal more specialist species occurring in spatially variable but stable environments (Kassen, 2002).

Previous studies have shown that plant populations can adapt to local environmental conditions already within a few centuries or even a decade (Carroll, Hendry, Reznick, & Fox, 2007; Linhart & Grant, 1996). This appears to be the case not only in native but also in non‐native species (see e.g. Colautti & Barrett, 2013; Maron et al., 2004). Our non‐native study species have probably been present in the Konstanz and Potsdam regions for more than 100 years (Table S19), and therefore, local adaptation should have had time to arise. However, in principle, local adaptation‐like patterns could also arise through several introduction events to different regions. For example, if cold‐adapted genotypes of a non‐native species are introduced to high latitudes and warm‐adapted genotypes to low latitudes. As we did not find clear patterns of local adaptation, it is not clear to what extent pre‐adaptation might have played a role in our non‐native study species.

As evidence for local adaptation was largely absent from our study, there were also no obvious differences in this regard between the five non‐native and the seven native species. Nevertheless, native‐non‐native status had a marginally non‐significant effect on the reproductive biomass (*p* = 0.056, Table 3), as effect sizes tended to be higher for some of the natives (cf. Table S12, Figures S3f and S4d). However, as these differences were very small, and not found for the other fitness components, we conclude that there were no clear differences in local adaptation between the native and non‐native ruderal species.

### 4.2 Effects of the demethylation agent zebularine

One of the best‐studied mechanisms of epigenetic inheritance in plants is DNA methylation (Hawes et al., 2018; Kilvitis et al., 2014; Schrey et al., 2013). Therefore, several studies have used demethylation agents, such as 5‐azacytidine and zebularine, to study the role of DNA methylation in transgenerational plasticity (Herman & Sultan, 2016; Verhoeven & van Gurp, 2012) and inbreeding depression (Vergeer, Wagemaker, & Ouborg, 2012). However, to the best of our knowledge, our study is the first one to use a demethylation agent to test for a potential epigenetic mechanism behind rapid local adaptation.

Local adaptation through epigenetic mutations (epimutations) is expected to be faster than through genetic mutations, since epimutation rates are several orders of magnitudes higher than normal mutation rates (cf. Johannes & Schmitz, 2018). One would therefore expect epimutations (e.g. changes in DNA methylation) to precede mutational changes to the genome (Richards, 2006). Depending on genomic context, DNA methylation can, for example, result in prolonged epigenetic silencing (Cubas et al., 1999; Schmitz et al., 2013; Verhoeven et al., 2010), and subsequently, genetic mutations in the affected gene region would be hidden from purifying selection (Arnheim & Calabrese, 2009; Diez, Roessler, & Gaut, 2014; Hwang & Green, 2004; Walsh & Xu, 2006). Therefore, adaptive methylation states may at the same time allow site‐specific genetic mutations to accumulate that could at a later stage, when methylations are removed, provide the raw material for genetic change (Hughes, 2012; Rodin & Riggs, 2003).

Although the limited evidence for local adaptation in our study prevents us from making inferences about the role of DNA methylation in local adaptation, zebularine‐treated plants overall had a lower biomass production than control plants (Tables S6 and S8, Figures S5‐S16). This reduced performance could reflect toxic side effects of zebularine (Baubec et al., 2009; Liu et al., 2015; Marquez, Barchi, et al., 2005; Marquez, Kelley, et al., 2005). However, it could also indicate that the zebularine treatment removed methylations of genes (see e.g. Cheng et al., 2003) that play a role in adaptation to a broad range of environmental conditions. Furthermore, some of the single‐species analyses revealed significant interactions of zebularine treatment with region and origin (Tables S5–S8, Figures S5–S16). These genotype‐ and environment‐specific effects of zebularine suggest that DNA methylation could still play a role in adaptation. Therefore, we conclude that more studies are needed on the potential role of DNA methylation and other epigenetic mechanisms in local adaptation.

## 5 CONCLUSIONS

Many studies in the last 70 years have conducted common‐garden and reciprocal transplant studies to test for population differentiation and local adaptation (Carroll et al., 2007; Clausen, Keck, & Hiesey, 1941, 1947; Hendry, Nosil, & Riesenberg, 2007; Hiesey, Clausen, & Keck, 1942; Linhart & Grant, 1996). Furthermore, numerous studies have tested for maternal carry‐over effects (Agrawal, Laforsch, & Tollrian, 1999) and adaptive transgenerational plasticity (Colicchio, 2017; Groot et al., 2017; Herman et al., 2012; Rendina González, Dumalasóva, Rosenthal, Skuhrovec, & Latzel, 2017). However, the potential ecological and evolutionary relevance of the epigenetic process gained attention only recently (Bossdorf et al., 2008; Hawes et al., 2018; Richards, 2011). Here, we studied whether DNA methylation can play a role in local adaptation, and particularly so in non‐native species, which might have had limited genetic variation and limited time to adapt by genetic change (Dlugosch, Anderson, Braasch, Cang, & Gillette, 2015; Richards et al., 2012; Richards, 2006; Suarez & Tsutsui, 2008). Our study, however, revealed little evidence for local adaptation overall and therefore also could not reveal whether there is a role for epigenetic mechanisms in local adaptation. Possibly, our results reflect that the ruderal species on which we focused are general‐purpose genotypes selected by the meta‐population dynamics in the ephemeral habitats in which they occur (Sultan & Spencer, 2002). To further assess the role of epigenetic mechanisms in local adaptation, we therefore call for studies that use species from more stable environments and preferably use study systems in which local adaptation in the invaded range has been demonstrated already (e.g. *Lythrum salicaria* or *Hypericum perforatum*) (Colautti & Barrett, 2013; Maron et al., 2004). For these species, it might also be interesting to compare whether the relative adaptive importance of epigenetic mechanisms differs between the native and the invaded ranges, and to use recently developed molecular tools to study changes in the methylation states of genes (Paun, Verhoeven, & Richards, 2019; Schield et al., 2016). Finally, it remains to be tested whether other mechanisms of epigenetic inheritance than DNA methylation can play a role in local adaptation.

## CONFLICT OF INTEREST

The authors have no competing interests to declare.

## AUTHORS' CONTRIBUTIONS

MvK, JJ, and MS designed the study. JH and SE performed the research and collected the data. JH analyzed the data with inputs from MvK and MS. All authors contributed substantially to the interpretation of the data. JH wrote the first draft of the manuscript and all authors contributed substantially to the revisions. All authors have approved the final manuscript draft for publication. JH and SE share first authorship.

## Data Availability

Data supporting the manuscript results were archived in the Dryad Digital Repository (DOI: https://doi.org/10.5061/dryad.8pv1309).
