# Supplementary material for: No evidence for local adaptation and an epigenetic underpinning in native and non‐native ruderal plant species in Germany
Source: Ecol Evol. 2019 Aug 6;9(17):9412–26. doi: 10.1002/ece3.5325 (PMC6745855; doi:10.1002/ece3.5325)
Supplement: Supplementary file 1 [file ECE3-9-9412-s001.docx]

## Supporting information

Article title: No evidence for local adaptation and an epigenetic underpinning in native and non-native ruderal plant species in Germany

Authors: Jasmin Herden, Silvia Eckert, Marc Stift, Jasmin Joshi and Mark van Kleunen

**Fig. S1** Zebularine concentration trial.

**Fig. S2** Layout of field sites.

**Fig. S3** Prediction plots of the meta-regression models in Table 3.

**Fig. S4** Forest plots with effect sizes summarized at the region level.

**Fig. S5** Reaction norms in *Amaranthus retroflexus*.

**Fig. S6** Reaction norms in *Chenopodium album*.

**Fig. S7** Reaction norms in *Erigeron canadensis.*

**Fig. S8** Reaction norms in *Erigeron annuus*.

**Fig. S9** Reaction norms in *Lactuca serriola*.

**Fig. S10** Reaction norms in *Senecio vulgaris*.

**Fig. S11** Reaction norms in *Sonchus oleraceus*.

**Fig. S12** Reaction norms in *Tripleurospermum inodorum*.

**Fig. S13** Reaction norms in *Veronica persica*.

**Fig. S14** Reaction norms in *Plantago major*.

**Fig. S15** Reaction norms in *Datura stramonium*.

**Fig. S16** Reaction norms in *Solanum nigrum*.

**Fig. S17** Soil parameters at field sites.

**Table S1** Number of used maternal lines per species and sampling region.

**Table S2** Notes on species-specific treatments (pre-sowing treatment + sowing).

**Table S3** Notes on time-line of the experiment (transplanting, planting and harvesting time line per species).

**Table S4** Description of the field sites.

**Table S5** Results of generalized linear mixed models for survival.

**Table S6** Results of linear mixed models for complete aboveground biomass.

**Table S7** Results of generalized linear mixed models for flowering probability.

**Table S8** Results of linear mixed models for reproductive biomass.

**Table S9** Estimates and confidence intervals of survival effect sizes summarized at the region-level, in Fig. S4A).

**Table S10** Estimates and confidence intervals of aboveground biomass effect sizes summarized at the region-level, in Fig. S4B).

**Table S11** Estimates and confidence intervals of flowering probability effect sizes summarized at the region-level, in Fig. S4C).

**Table S12** Estimates and confidence intervals of reproductive biomass effect sizes summarized at the region-level, in Fig. S4D).

**Table S13** Estimates and confidence intervals of across-region summarized effect sizes for survival (Fig. 1A).

**Table S14** Estimates and confidence intervals of across-region summarized effect sizes for aboveground biomass (Fig. 1B).

**Table S15** Estimates and confidence intervals of across-region summarized effect sizes for flowering probability (Fig. 1C).

**Table S16** Estimates and confidence intervals of across-region summarized effect sizes for reproductive biomass (Fig. 1D).

**Table S17** Soil parameters at field sites.

**Table S18** Climatic differences

**Table S19** First record of the non-native study species in Germany and in the respective regions.

**Methods S1** Single-species analysis of fitness parameters

**Methods S2** Effect size calculation in meta-regression

**Methods S3** Effect sizes corrected with the ratio of sample sizes of the compared groups **Methods S4** Soil parameter measurements in field sites

**Notes S1** Results of the single species analyses

**Notes S2** Results of effect sizes summarized at the region-level.

**Fig. S1** Zebularine concentration trial.

Exemplary gradients of zebularine treatment during germination for study species. From left to right: 0 µM (control), 12.5 µM, 25 µM, 50 µM and 100 µM. Based on these gradients we decided on a concentration between 25 and 50 µM zebularine.


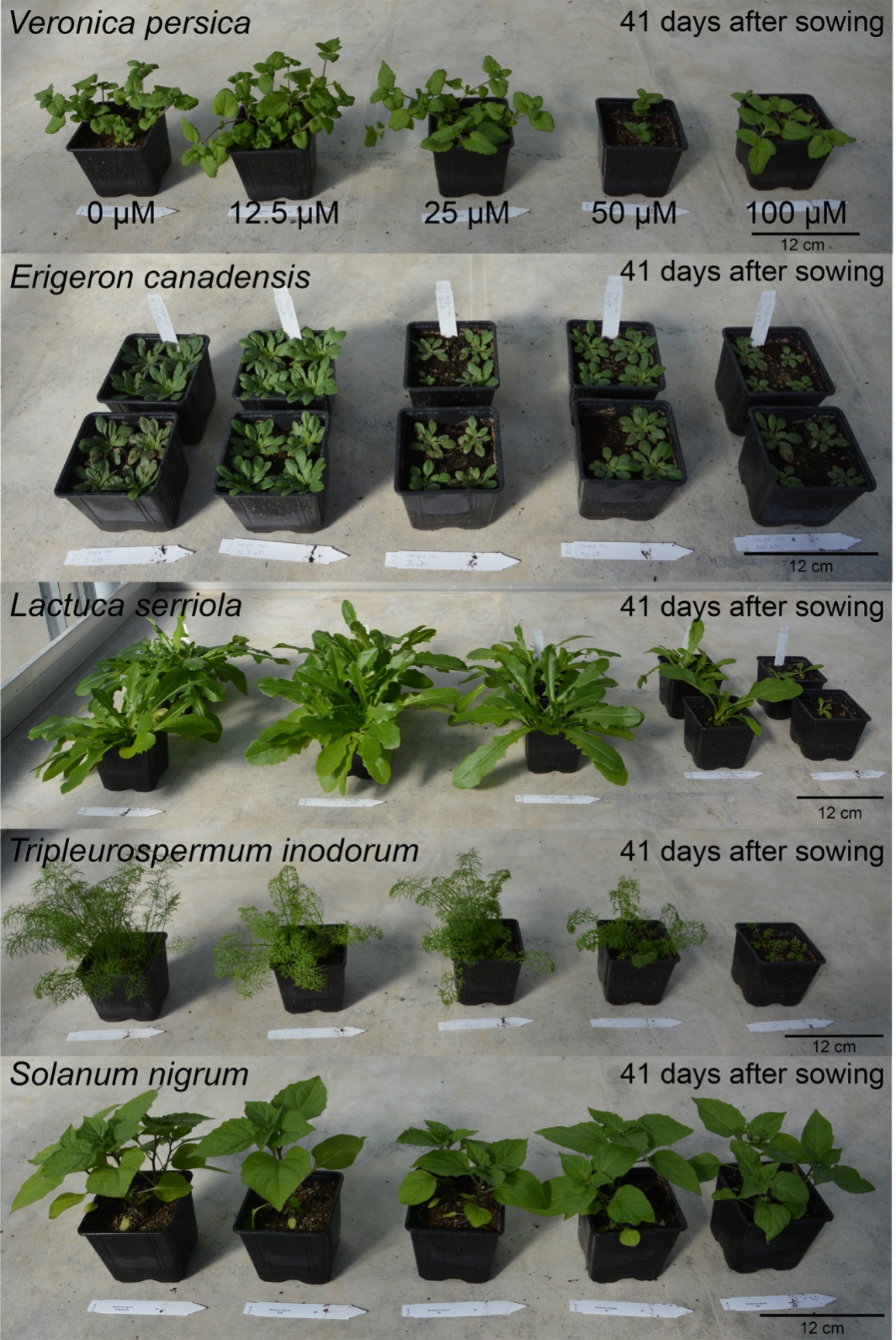


**Fig. S2** Layout of field sites. As an example, the positions of blocks and species plots at the Botanical Garden Konstanz field site are shown (A). An example of random allocation of control and zebularine-treated local and non-local plants to positions within a species plot (B).

#####
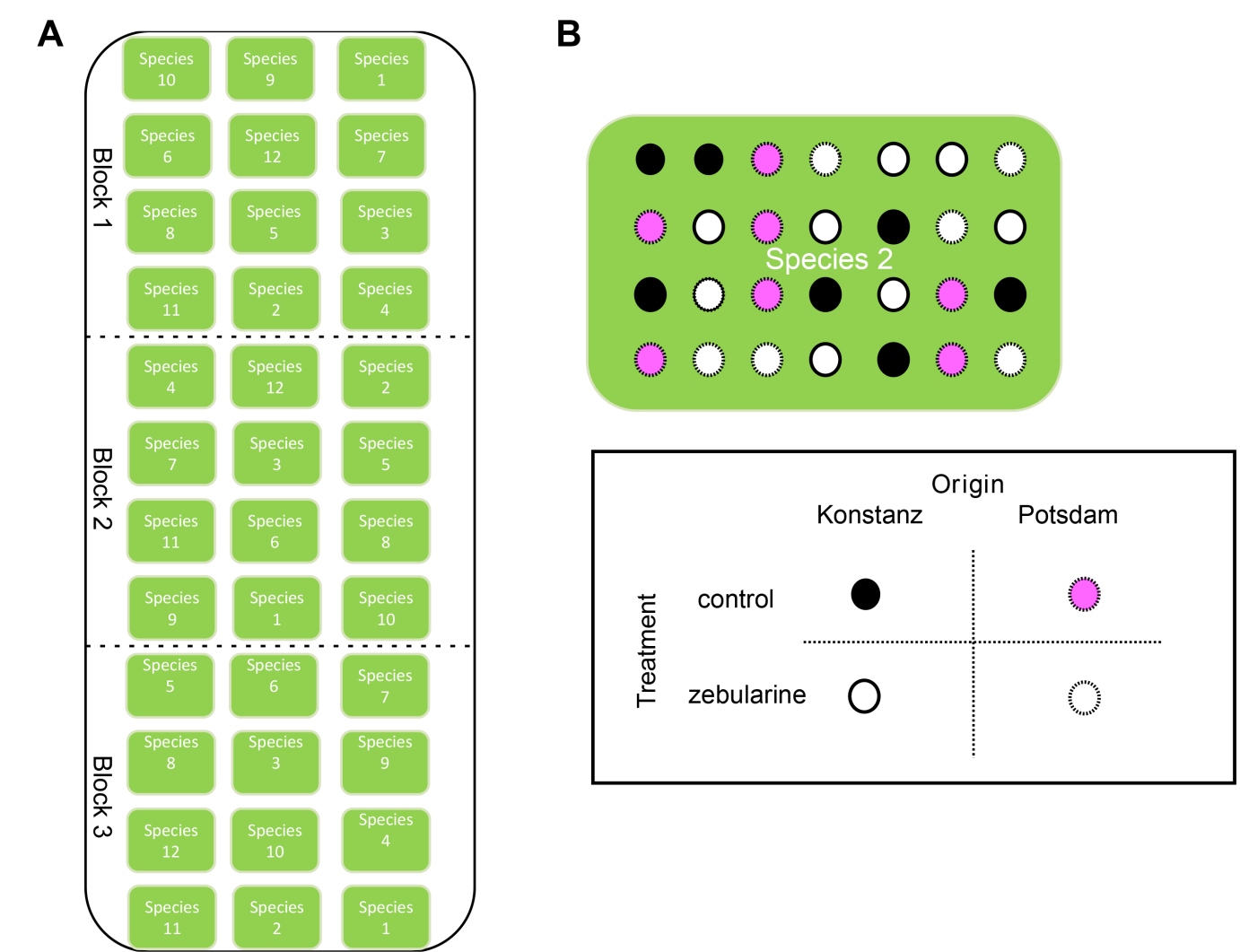


**Fig. S3** Prediction plots of the meta-regression models in Table 3. Plotted predictions for native and non-native plant species in the control and zebularine treatment in Konstanz and Potsdam, based on meta-regression models in Table 3. Squares illustrate Konstanz data and triangles illustrate Potsdam data. Closed and open symbols stand for control and zebularine treatments, respectively. Natives are marked in black and non-natives are marked in red. Stars denote effect sizes significantly different from 0 (i.e. 95% confidence intervals non-overlapping with 0). Survival model with 0.5 continuity correction (A), survival model with continuity correction based on the ratio of sample sizes between the compared groups (B), aboveground biomass model (C), flowering probability model with 0.5 continuity correction (D), flowering probability model with continuity correction based on the ratio of sample sizes between the compared groups (E), and reproductive biomass model (F).


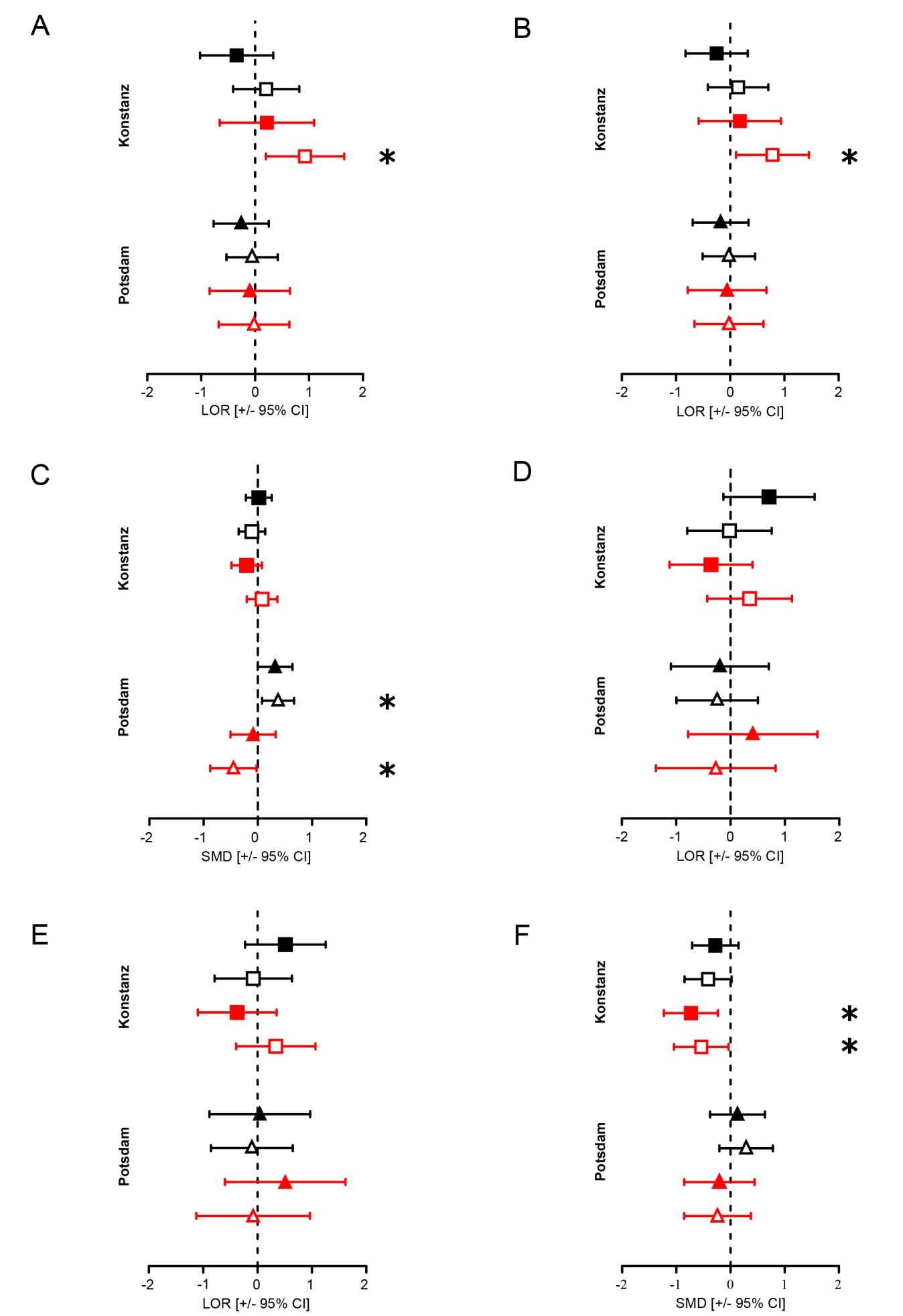


**Fig. S4** Forest plots with effect sizes of survival (A), aboveground biomass (B), flowering probability (C) and reproductive biomass summarized at the region-level (D). Squares illustrate Konstanz data and triangles illustrate Potsdam data. Closed and open symbols stand for control and zebularine treatment, respectively. Natives are marked in black and non-natives are marked in red. Stars denote effect sizes significantly different from 0 (i.e. 95% confidence intervals non-overlapping with 0). Green arrows denote cases with a single effect size, without summarization. NAs denote cases with insufficient data for effect size calculation. Survival and flowering probability effect sizes were calculated with continuity correction based on the ratio of sample sizes between the compared groups. LOR – log-transformed odds ratio, SMD – standardized mean difference.


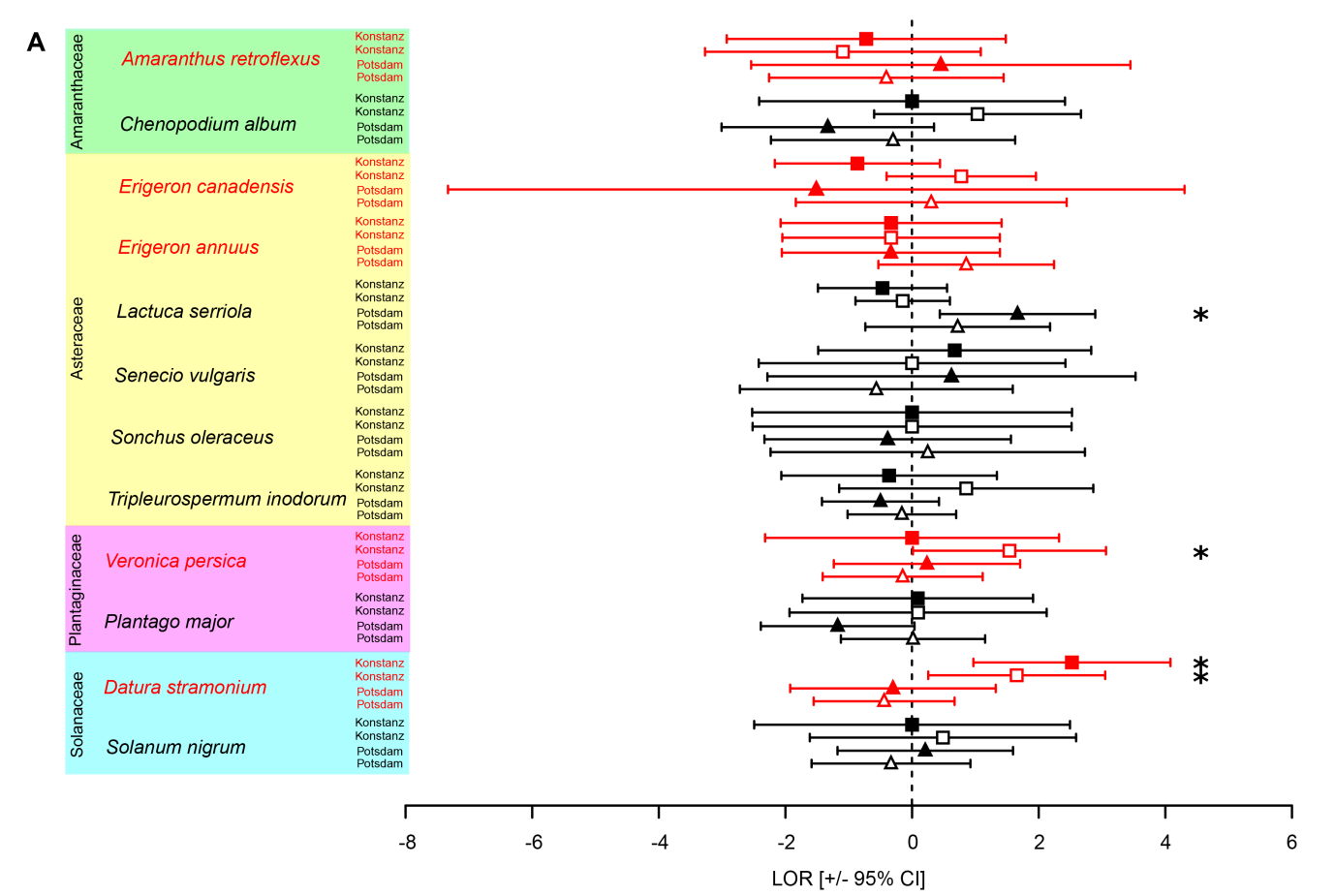

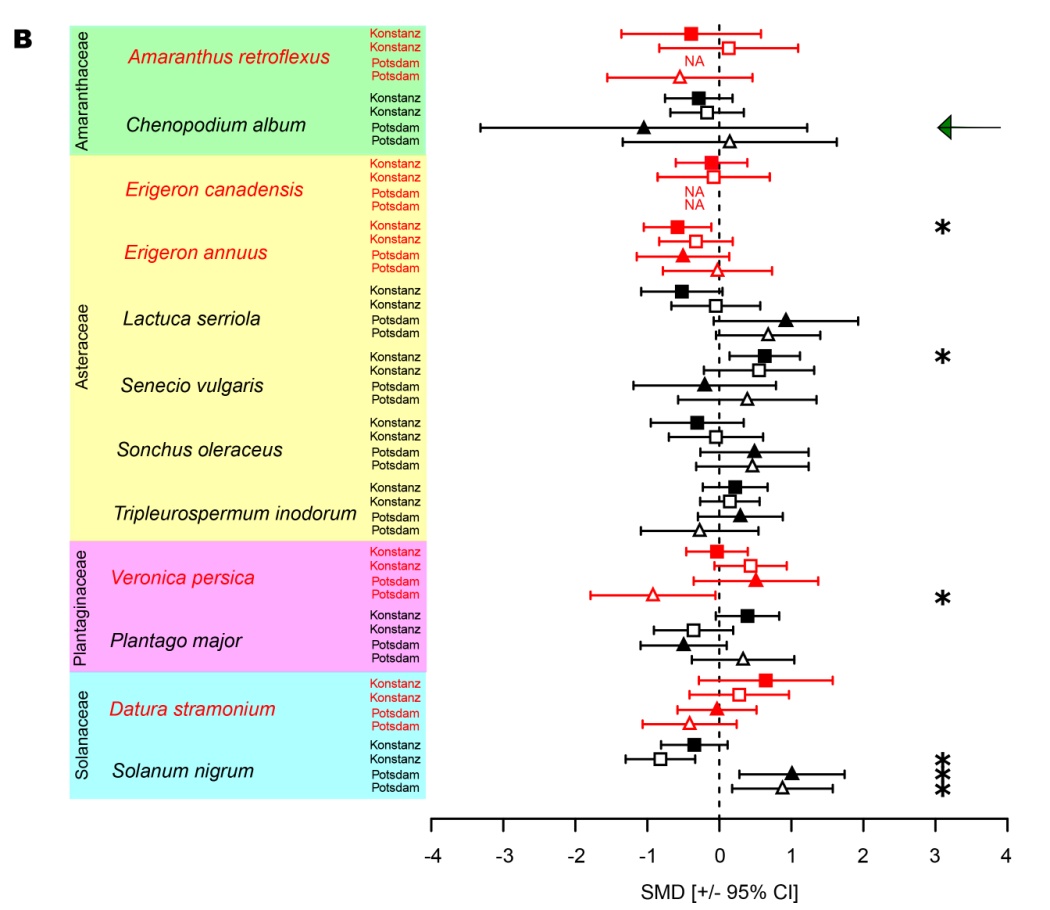

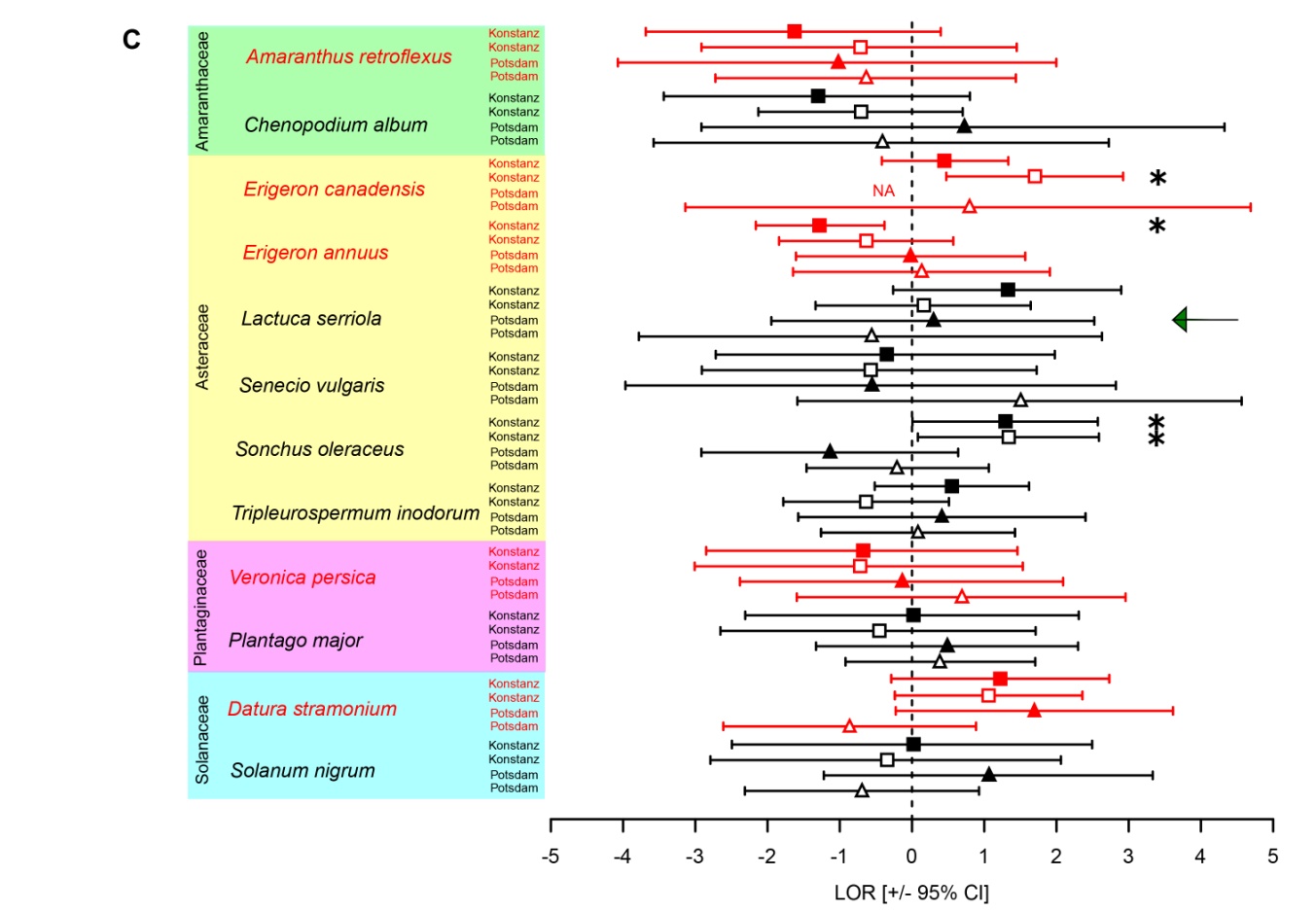

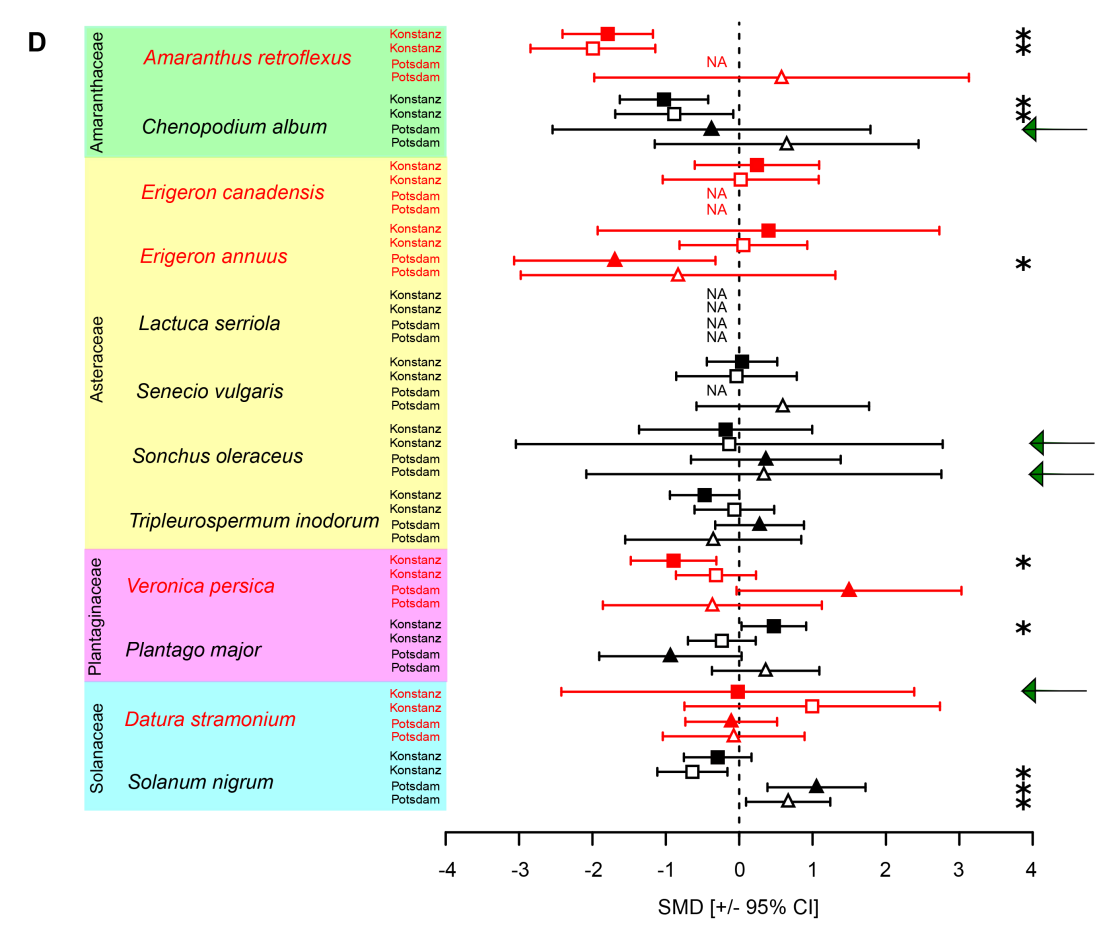


**Fig. S5** Reaction norms of the non-native species *Amaranthus retroflexus* for survival (A), aboveground biomass (B), flowering probability (C) and reproductive biomass (D). Mean and 95% confidence intervals for untransformed data. Closed and open symbols denote control and zebularine treatment, respectively. Squares indicate Konstanz site data and triangles indicate Potsdam site data. Reaction norm for origins in the Konstanz transplant region are indicated in black, and origins in the Potsdam transplant region are indicated in purple.

#####
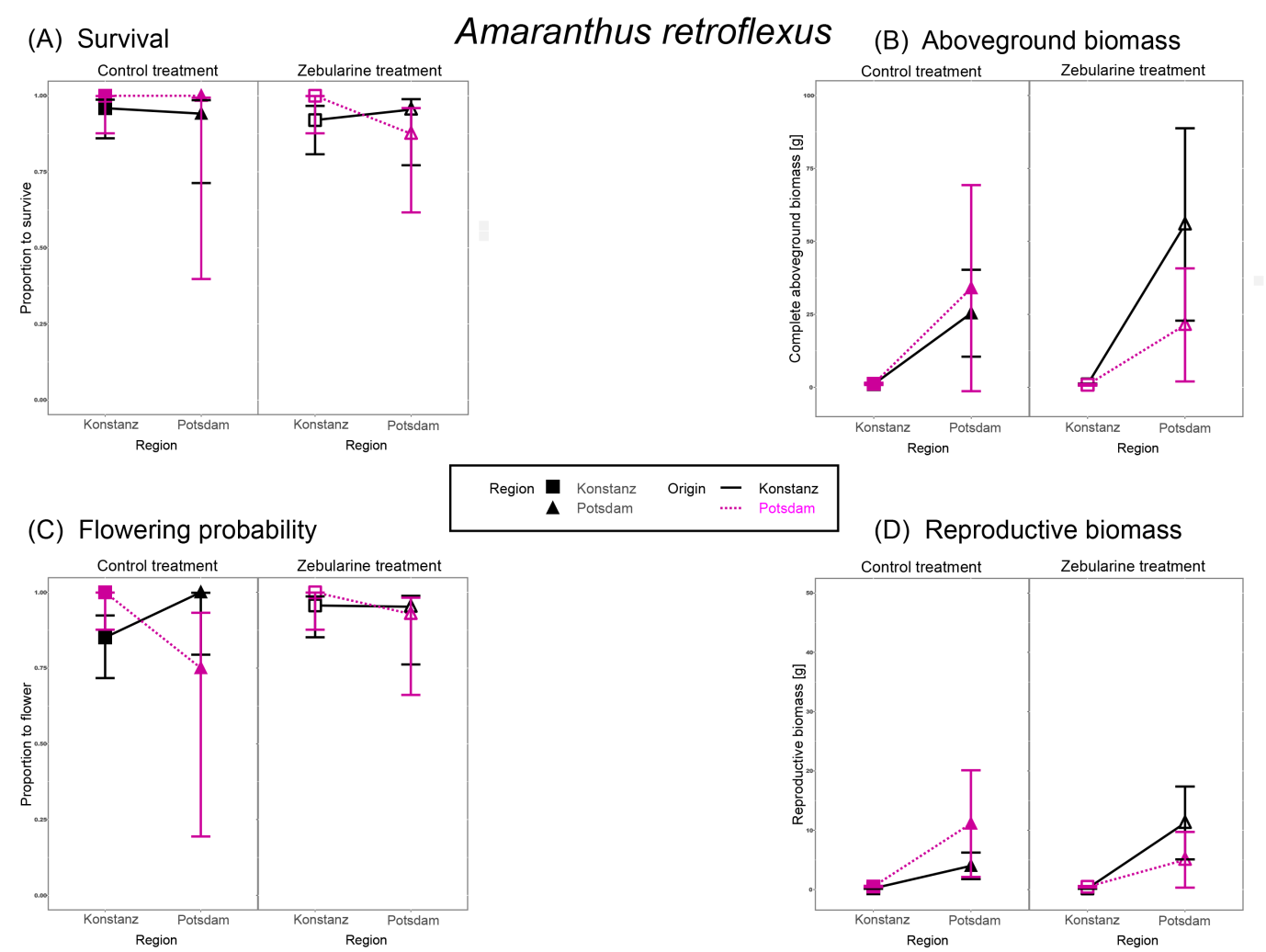


**Fig. S6** Reaction norms of the native species *Chenopodium album* for survival (A), aboveground biomass (B), flowering probability (C) and reproductive biomass (D). Mean and 95% confidence intervals for untransformed data. Closed and open symbols denote control and zebularine treatment, respectively. Squares indicate Konstanz region data and triangles indicate Potsdam region data. Reaction norm for origins in the Konstanz transplant region are indicated in black, and origins in the Potsdam transplant region are indicated in purple.

#####
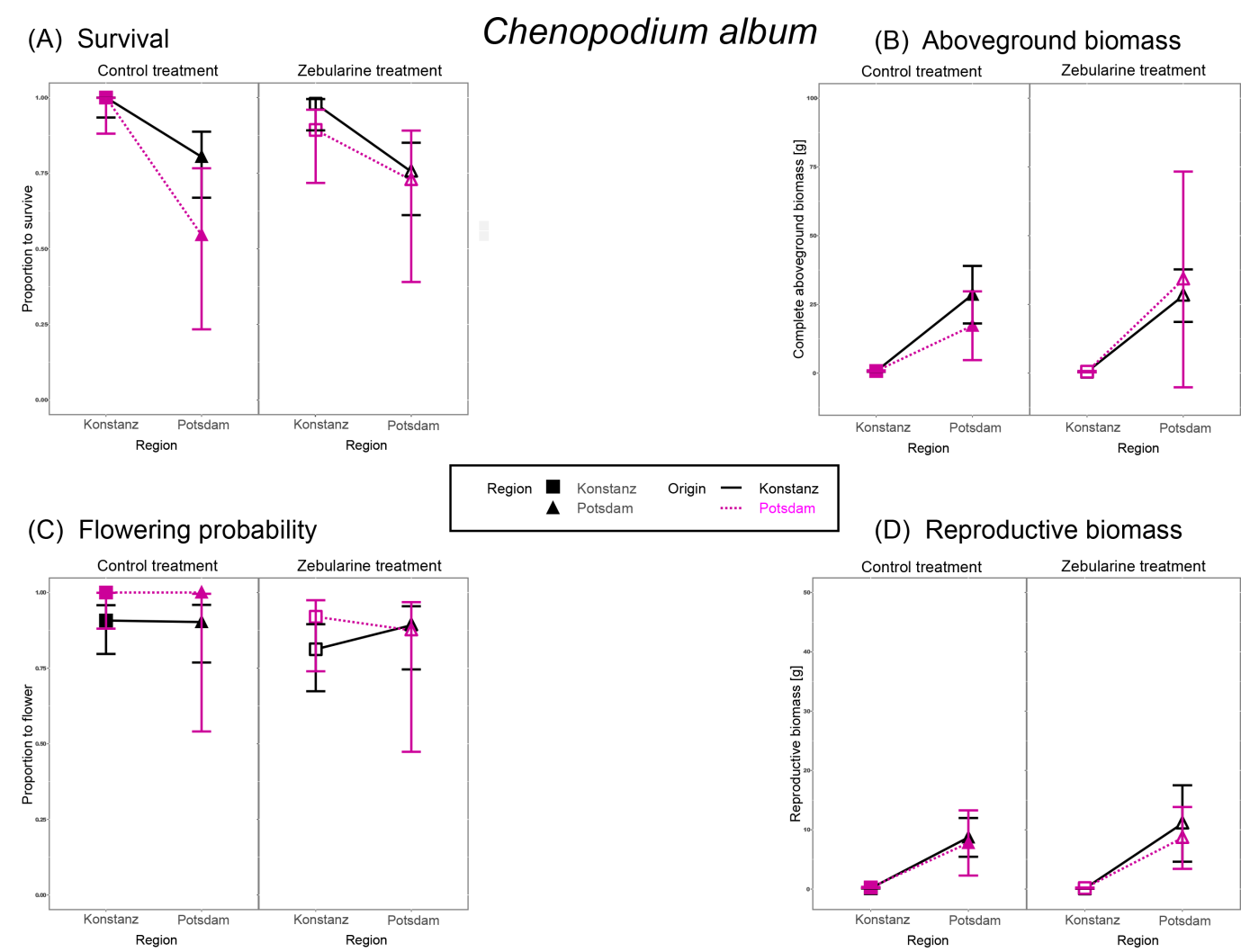


**Fig. S7** Reaction norms of the non-native species *Erigeron canadensis* for survival (A), aboveground biomass (B), flowering probability (C) and reproductive biomass (D). Mean and 95% confidence intervals for untransformed data. Closed and open symbols denote control and zebularine treatment, respectively. Squares indicate Konstanz region data and triangles indicate Potsdam region data. Reaction norm for origins in the Konstanz transplant region are indicated in black, and origins in the Potsdam transplant region are indicated in purple.

#####
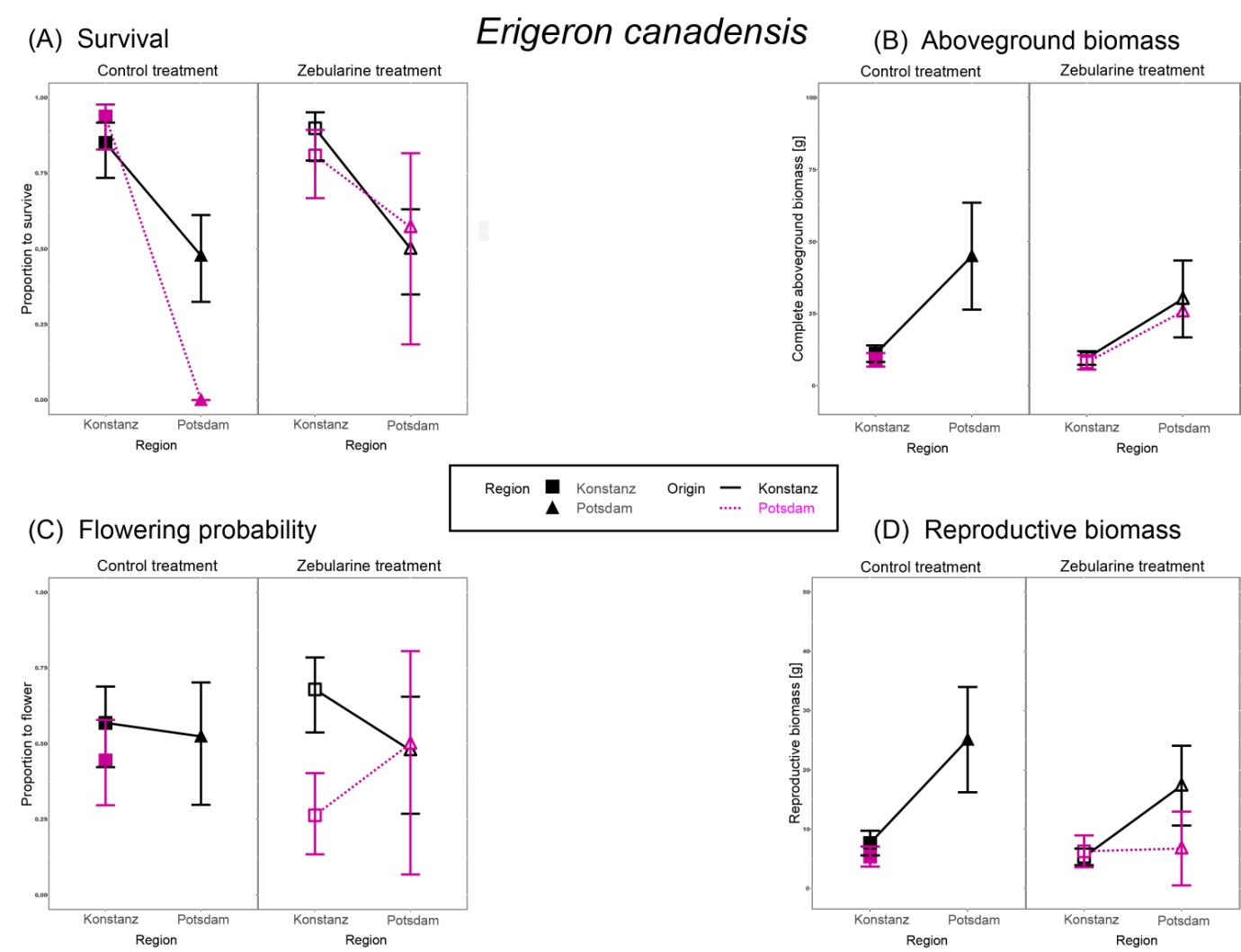


**Fig. S8** Reaction norms of the non-native species *Erigeron annuus* for survival (A), aboveground biomass (B), flowering probability (C) and reproductive biomass (D). Mean and 95% confidence intervals for untransformed data. Closed and open symbols denote control and zebularine treatment, respectively. Squares indicate Konstanz region data and triangles indicate Potsdam region data. Reaction norm for origins in the Konstanz transplant region are indicated in black, and origins in the Potsdam transplant region are indicated in purple.

#####
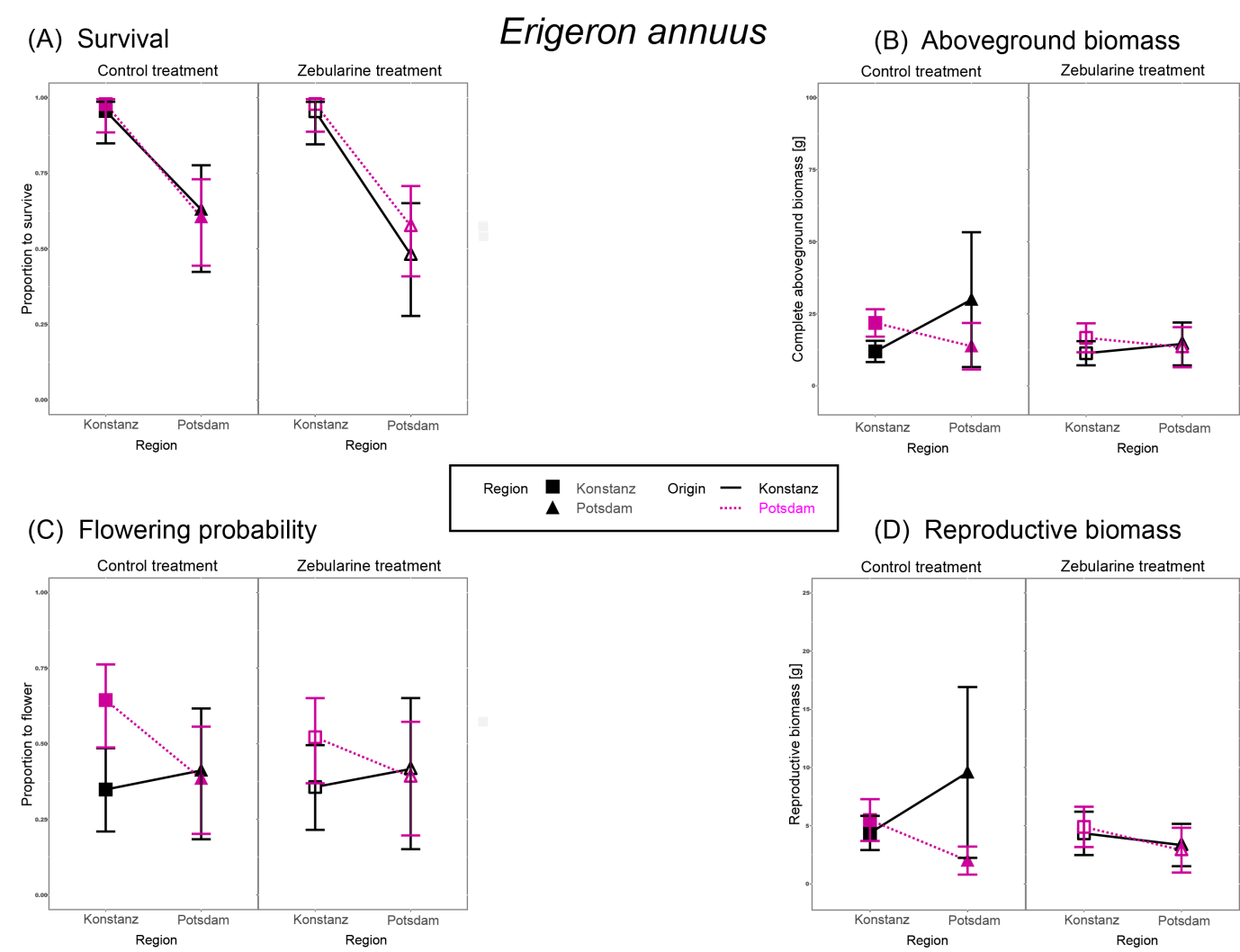


**Fig. S9** Reaction norms of the native species *Lactuca serriola* for survival (A), aboveground biomass (B), flowering probability (C) and reproductive biomass (D). Mean and 95% confidence intervals for untransformed data. Closed and open symbols denote control and zebularine treatment, respectively. Squares indicate Konstanz region data and triangles indicate Potsdam region data. Reaction norm for origins in the Konstanz transplant region are indicated in black, and origins in the Potsdam transplant region are indicated in purple.

#####
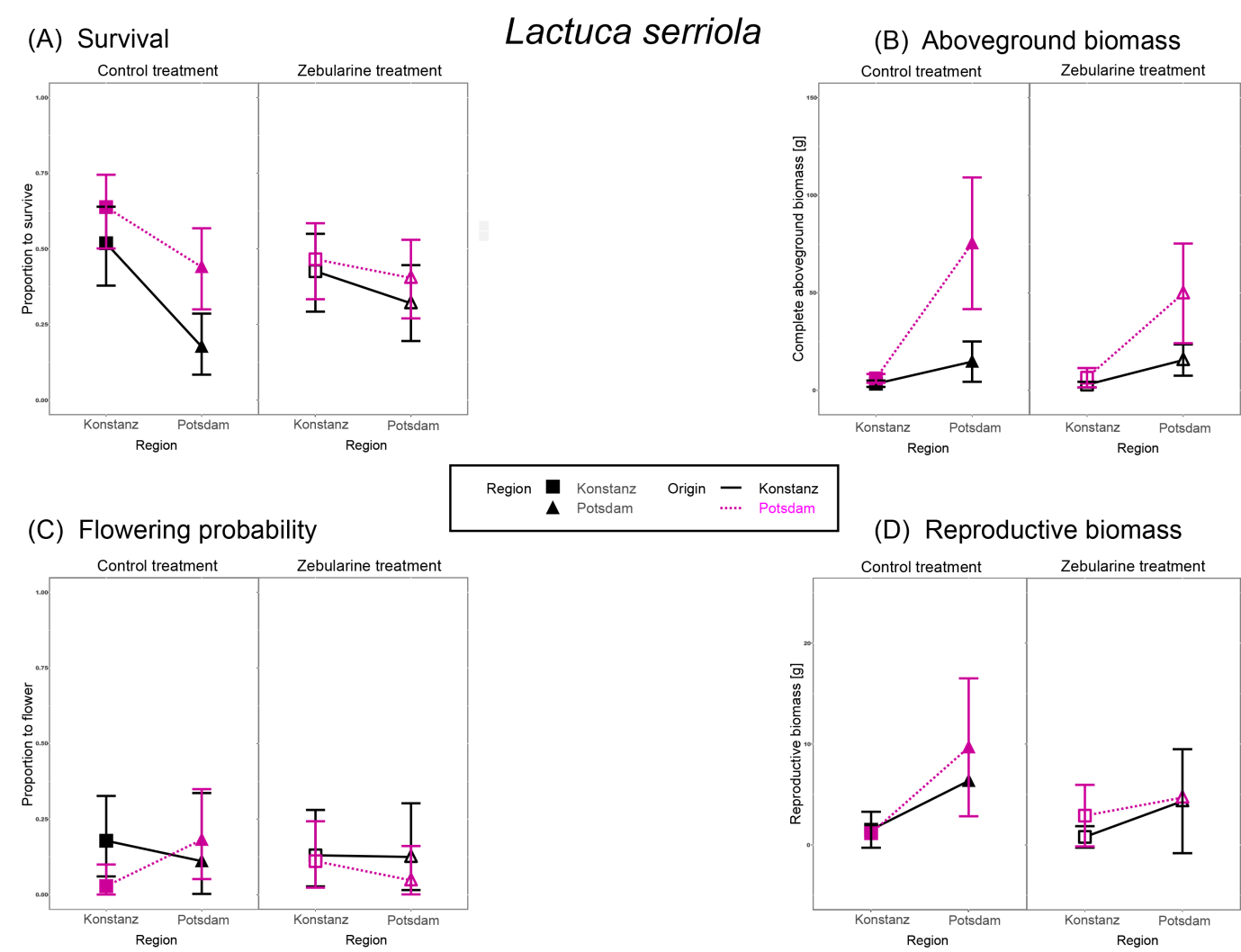


**Fig. S10** Reaction norms of the native species *Senecio vulgaris* for survival (A), aboveground biomass (B), flowering probability (C) and reproductive biomass (D). Mean and 95% confidence intervals for untransformed data. Closed and open symbols denote control and zebularine treatment, respectively. Squares indicate Konstanz region data and triangles indicate Potsdam region data. Reaction norm for origins in the Konstanz transplant region are indicated in black, and origins in the Potsdam transplant region are indicated in purple.

#####
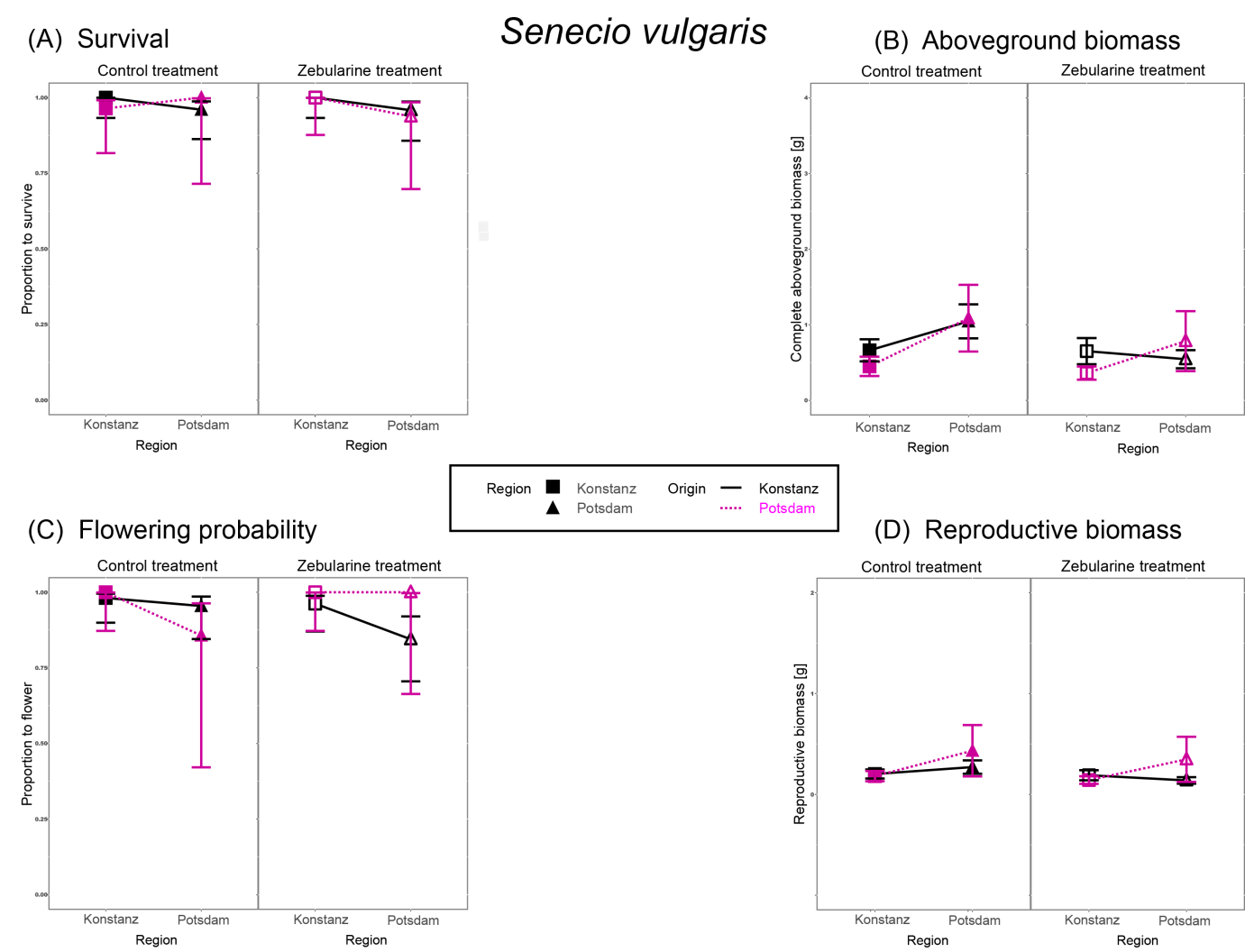


**Fig. S11** Reaction norms of the native species *Sonchus oleraceus* for survival (A), aboveground biomass (B), flowering probability (C) and reproductive biomass (D). Mean and 95% confidence intervals for untransformed data. Closed and open symbols denote control and zebularine treatment, respectively. Squares indicate Konstanz region data and triangles indicate Potsdam region data. Reaction norm for origins in the Konstanz transplant region are indicated in black, and origins in the Potsdam transplant region are indicated in purple.

#####
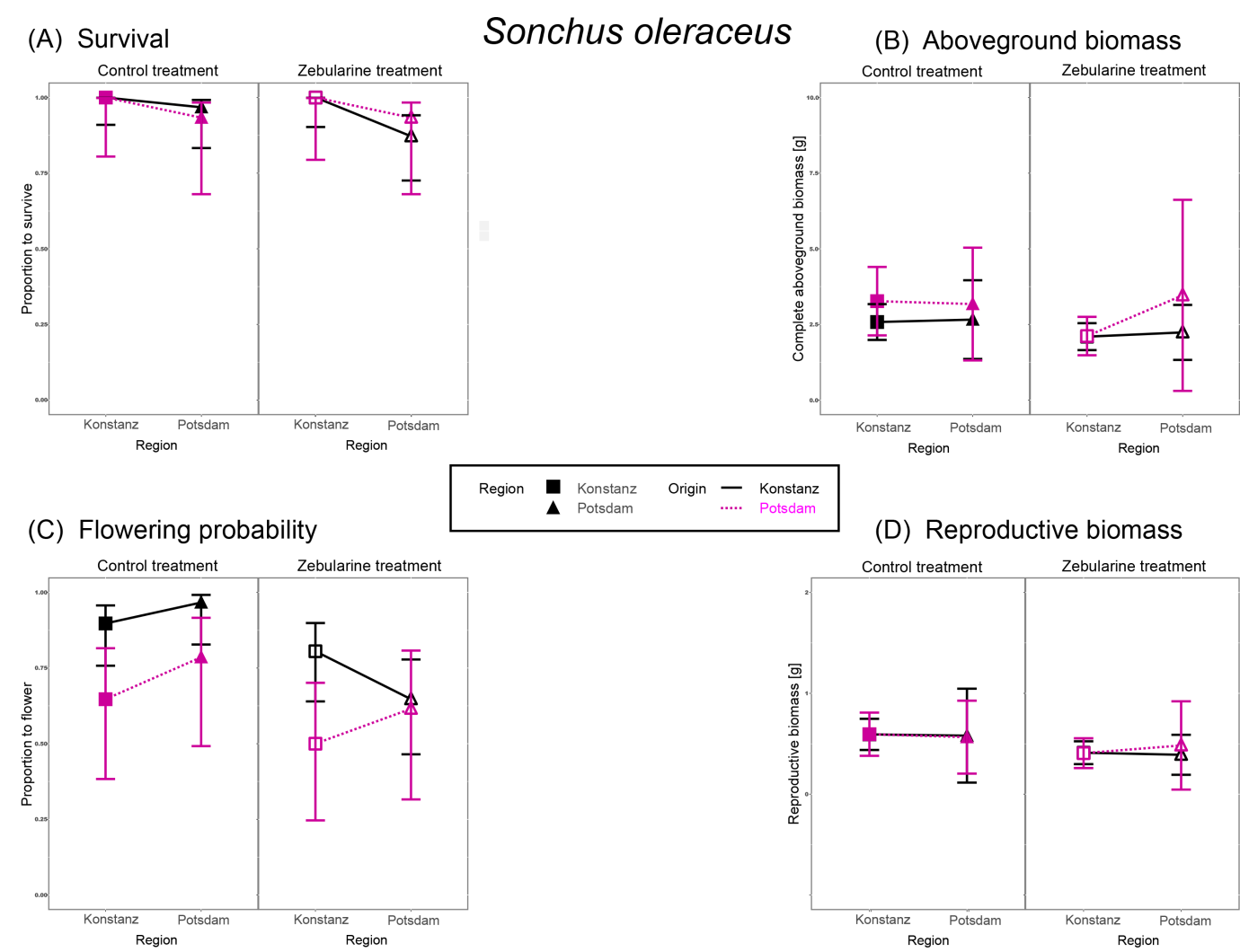


**Fig. S12** Reaction norms of the native species *Tripleurospermum inodorum* for survival (A), aboveground biomass (B), flowering probability (C) and reproductive biomass (D). Mean and 95% confidence intervals for untransformed data. Closed and open symbols denote control and zebularine treatment, respectively. Squares indicate Konstanz region data and triangles indicate Potsdam region data. Reaction norm for origins in the Konstanz transplant region are indicated in black, and origins in the Potsdam transplant region are indicated in purple.

#####
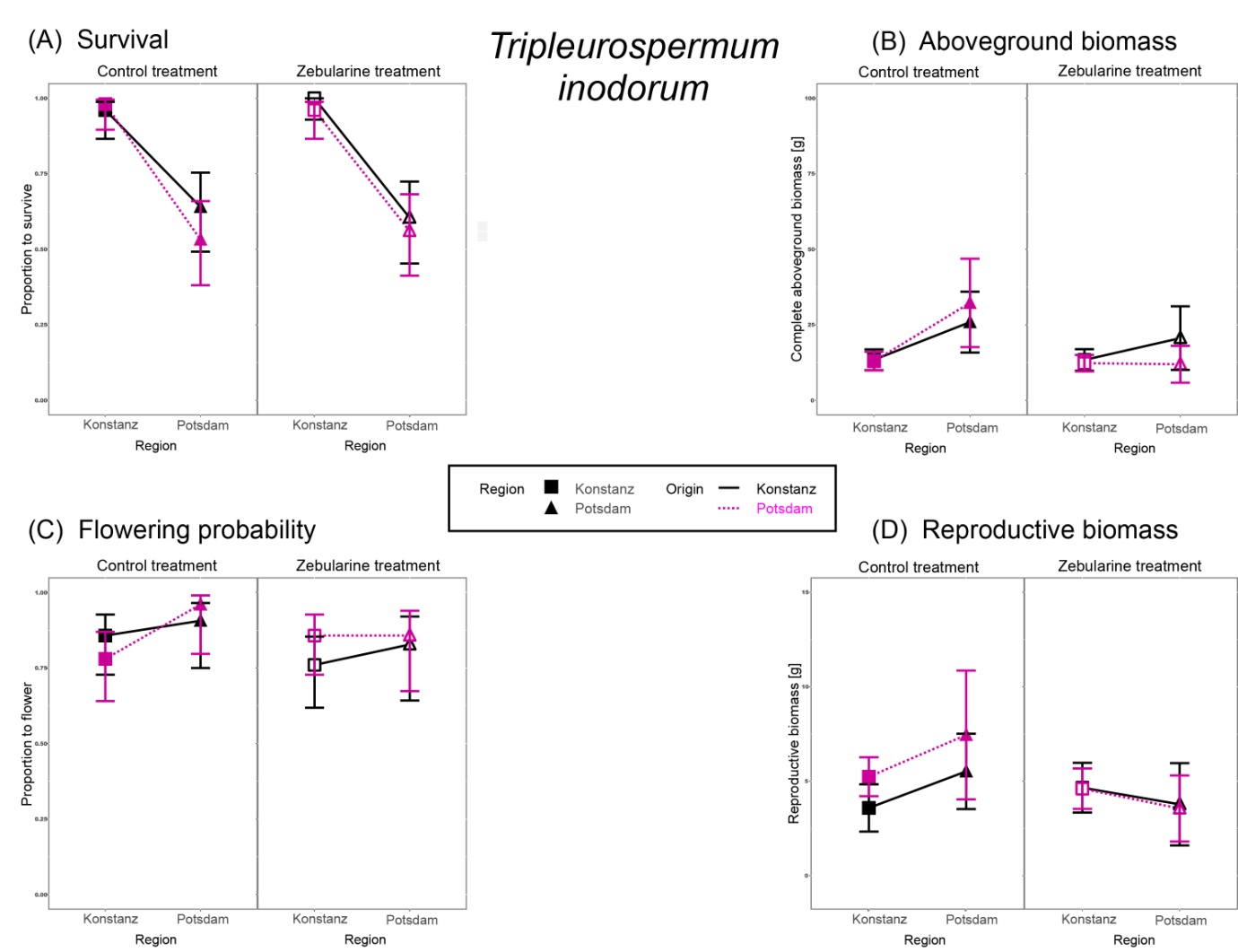


**Fig. S13** Reaction norms of the non-native species *Veronica persica* for survival (A), aboveground biomass (B), flowering probability (C) and reproductive biomass (D). Mean and 95% confidence intervals for untransformed data. Closed and open symbols denote control and zebularine treatment, respectively. Squares indicate Konstanz region data and triangles indicate Potsdam region data. Reaction norm for origins in the Konstanz transplant region are indicated in black, and origins in the Potsdam transplant region are indicated in purple.

#####
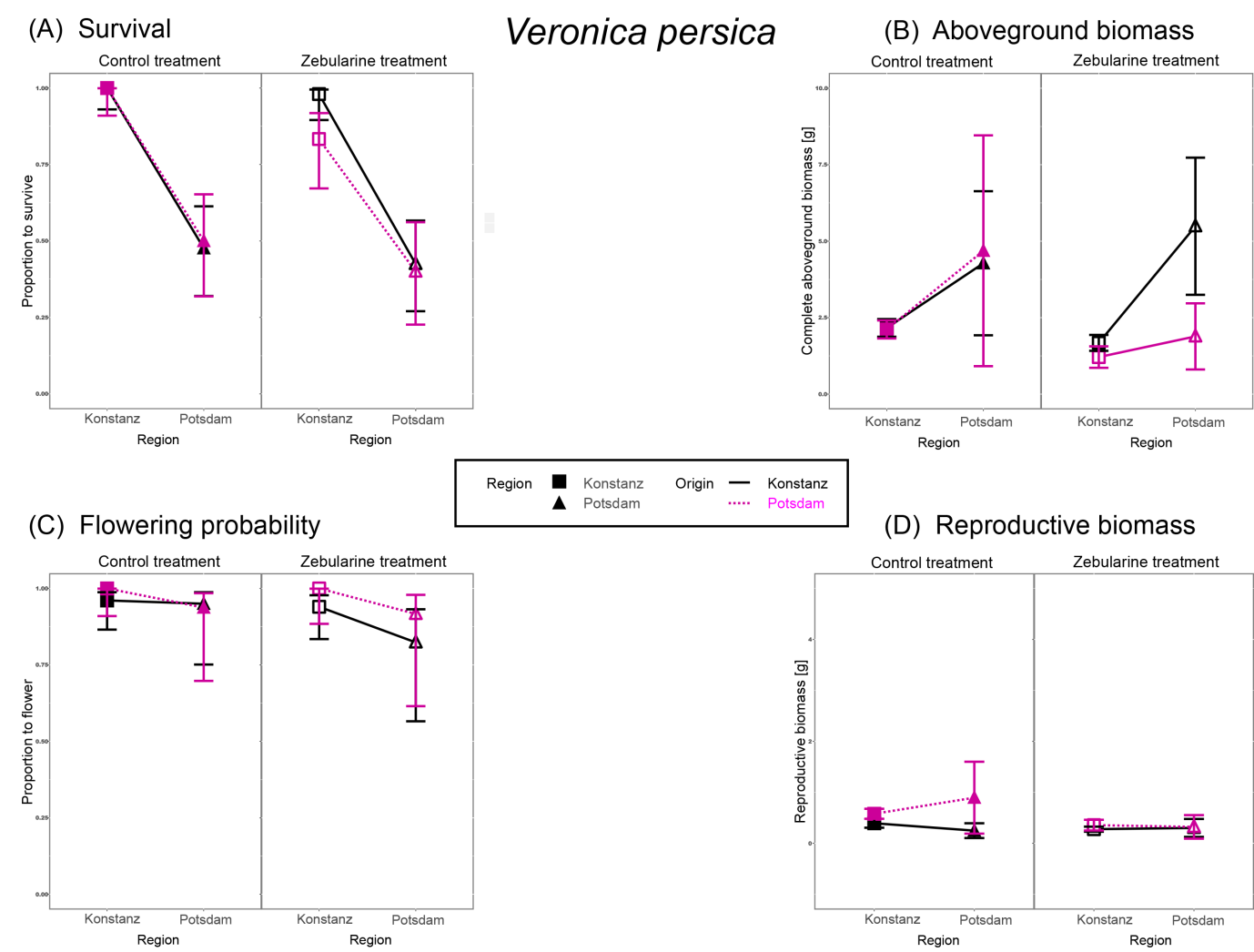


**Fig. S14** Reaction norms of the native species *Plantago major* for survival (A), aboveground biomass (B), flowering probability (C) and reproductive biomass (D). Mean and 95% confidence intervals for untransformed data. Closed and open symbols denote control and zebularine treatment, respectively. Squares indicate Konstanz region data and triangles indicate Potsdam region data. Reaction norm for origins in the Konstanz transplant region are indicated in black, and origins in the Potsdam transplant region are indicated in purple.

#####
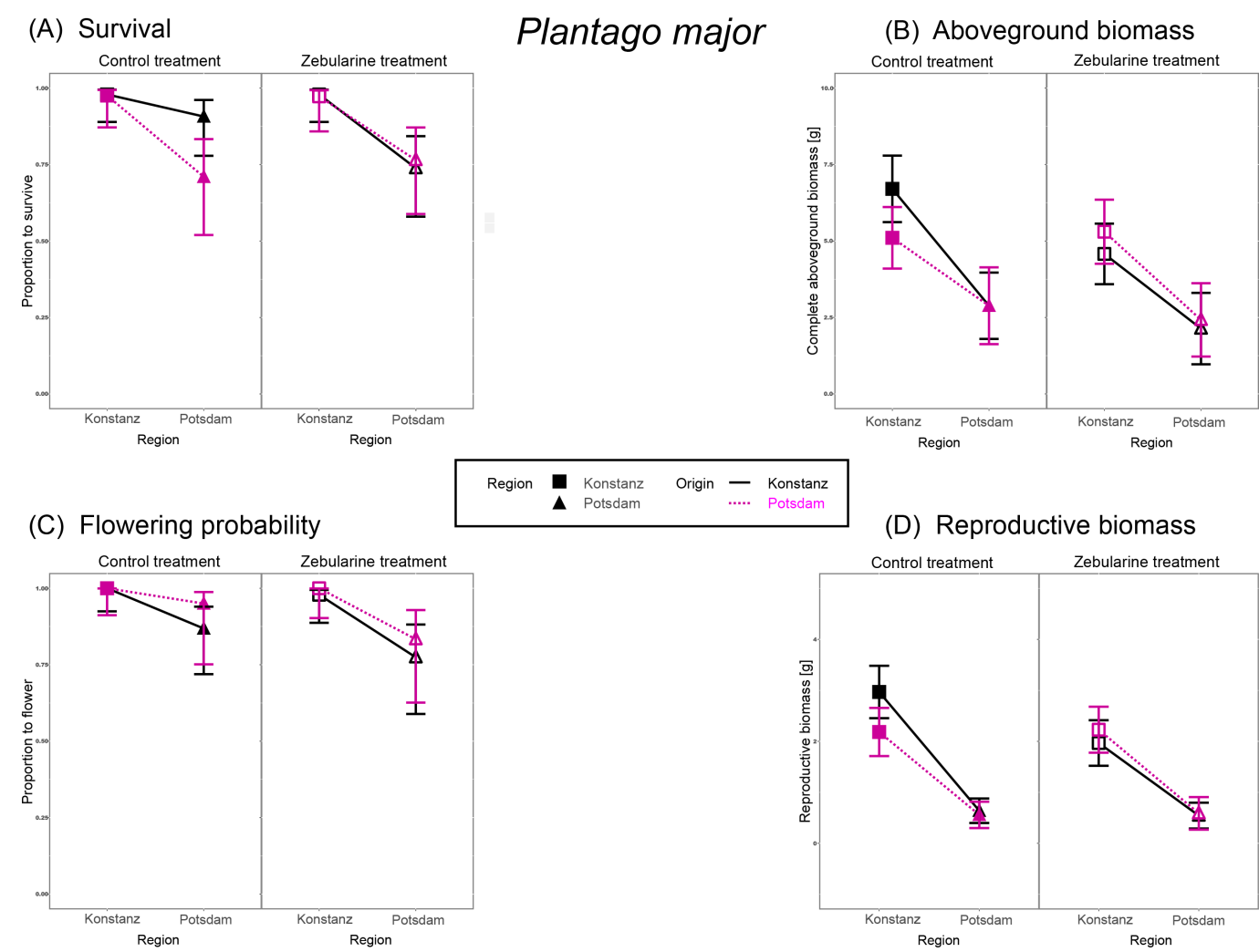


**Fig. S15** Reaction norms of the non-native species *Datura stramonium* for survival (A), aboveground biomass (B), flowering probability (C) and reproductive biomass (D). Mean and 95% confidence intervals for untransformed data Closed and open symbols denote control and zebularine treatment, respectively. Squares indicate Konstanz region data and triangles indicate Potsdam region data. Reaction norm for origins in the Konstanz transplant region are indicated in black, and origins in the Potsdam transplant region are indicated in purple.

#####
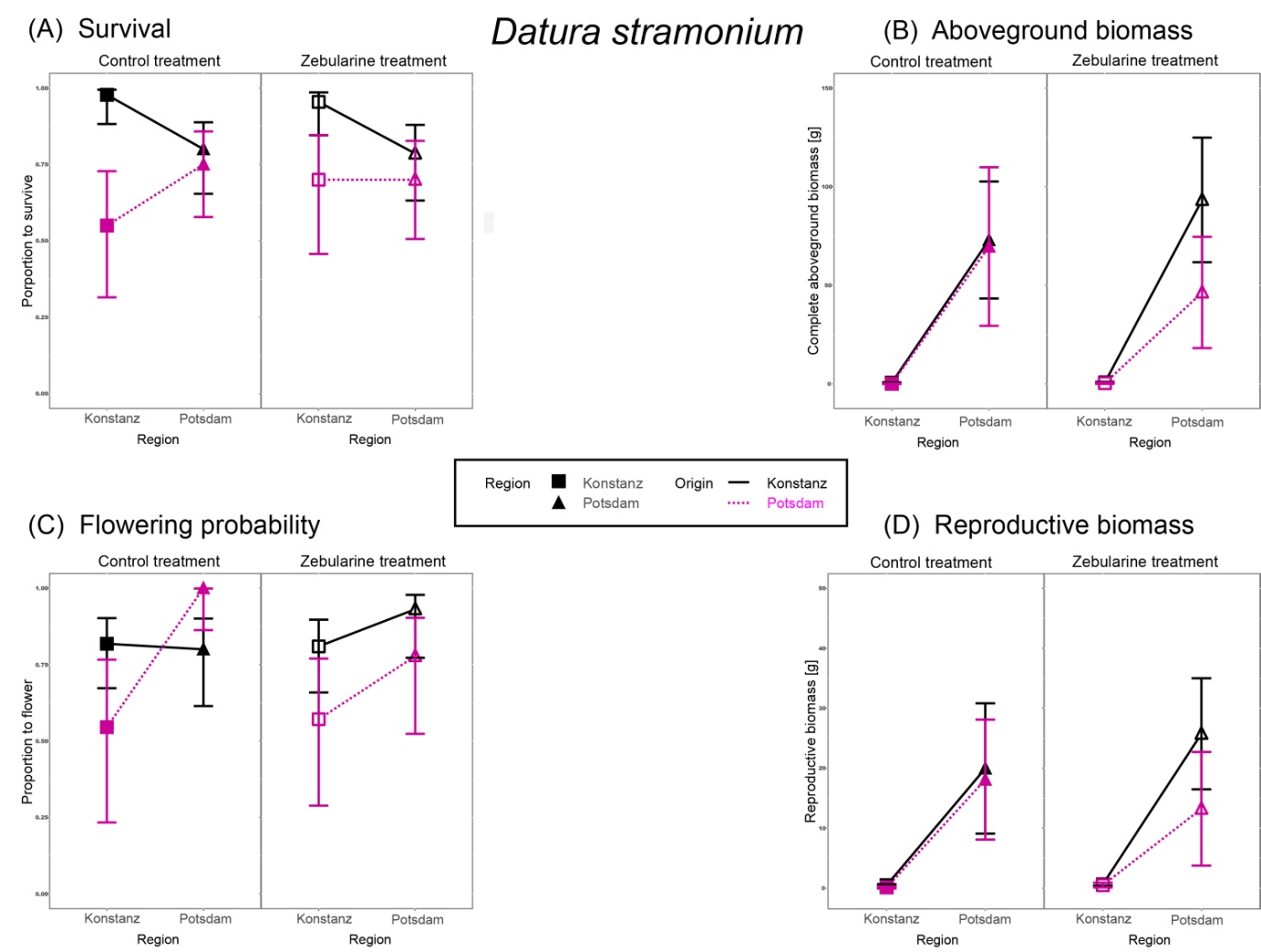


**Fig. S16** Reaction norms of the native species *Solanum nigrum* for survival (A), aboveground biomass (B), flowering probability (C) and reproductive biomass (D). Mean and 95% confidence intervals for untransformed data. Closed and open symbols denote control and zebularine treatment, respectively. Squares indicate Konstanz region data and triangles indicate Potsdam region data. Reaction norm for origins in the Konstanz transplant region are indicated in black, and origins in the Potsdam transplant region are indicated in purple.

**
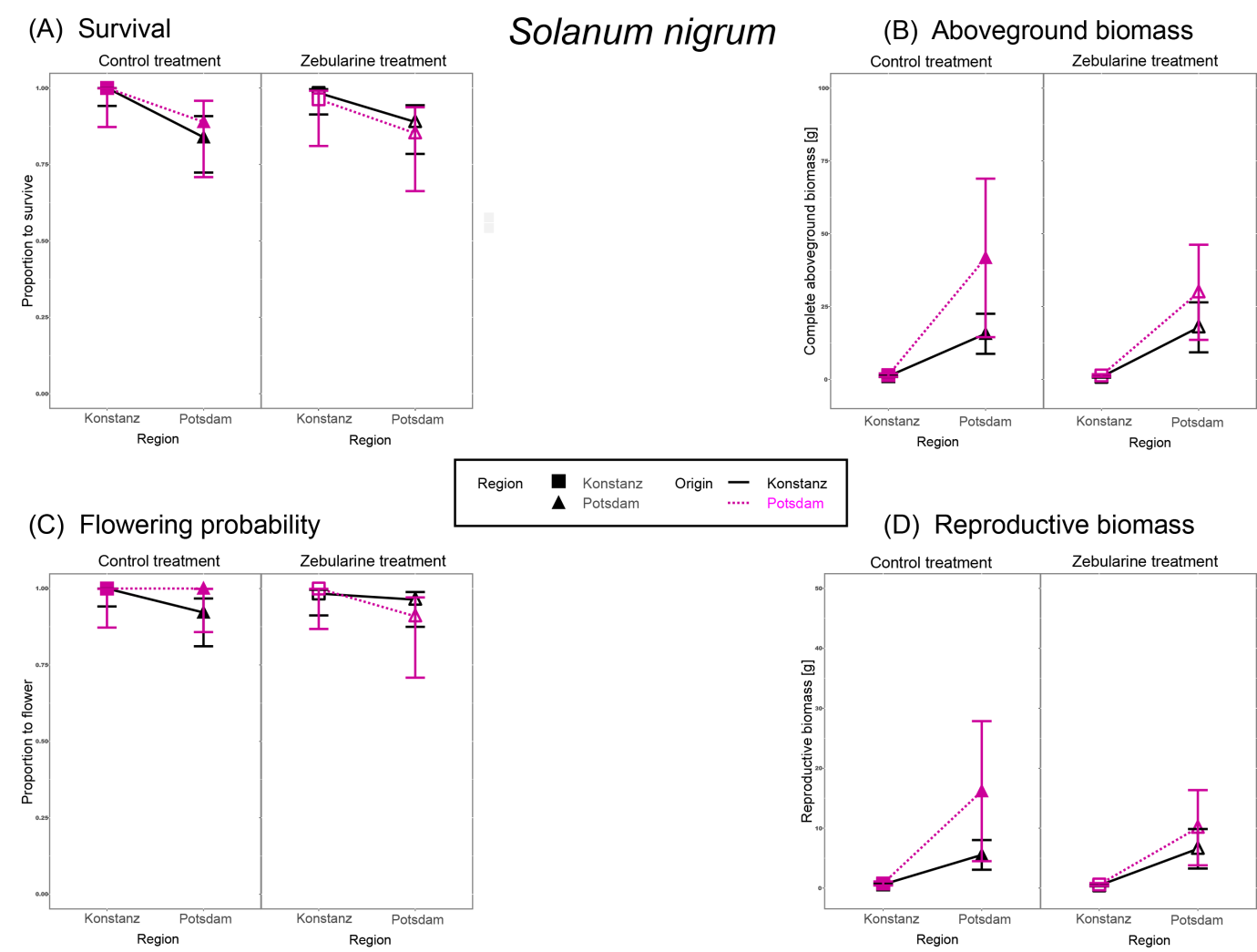
**

**Fig. S17** Soil parameters at field sites. Scree plot (A) and PCA and loadings for soil parameters at transplant field sites (B). BoGa – Botanical Garden of Konstanz; DB - Potsdam/Botanical Garden; GR - Ludwigsfelde/Gröben (close to Potsdam); Hättli - Konstanz/Hättelihof; MQ - Potsdam-Marquardt; Uni – University of Konstanz; Ammonium – plant-available ammonium [mg kg^-1^ dry mass]; Cpercent – relative [%] carbon content of dry mass; Npercent – relative [%] nitrogen content of dry mass; CNratio – carbon-to-nitrogen ratio of dry mass; Nitrogen – total nitrogen content [g kg^-1^ dry mass]; OrganicMatter – relative [%] organic matter in dry mass after loss-on-ignition; pHcurrent – pH value in ddH_2_O; pHpotential – pH value in CaCl_2_^-^; Phosphate – total phosphate content [g kg^-1^ dry mass]; PhosphatePA – plant-available phosphate content [mg L^-1^ solved dry mass]; WeightRatio – dry-to-fresh mass ratio. (See Methods S4 for a detailed description of sampling and soil analysis.)


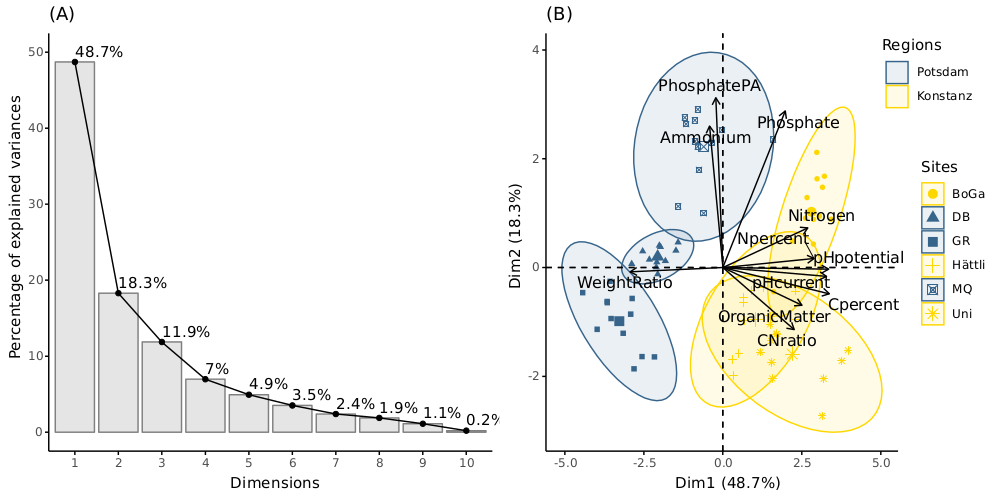


**Table S1** Number of used maternal lines per species and sampling region. K – Konstanz transplant region; P – Potsdam transplant region. Species names of the natives are in black, and the ones of the non-natives are in red.

| **Population** | **Family** | **Species** | **Source** | **Maternal lines** | **Location** | **Latitude** | **Longitude** |
| --- | --- | --- | --- | --- | --- | --- | --- |
| **1** | Amaranthaceae | *Amaranthus retroflexus* | K | 17 | Twielfeld, Singen | 47.76131 | 8.820415 |
| **2** |  |  | P | 10 | Karzow, fallow land close to Fahrländer Chaussee | 52.49144 | 12.98392 |
| **3** |  | *Chenopodium album* | K | 19 | Singen, Im Moosfeld | 47.71661 | 8.908667 |
| **4** |  |  | P | 20 | Potsdam, 14469, Maulbeerallee 2, Botanical Garden | 52.40323 | 13.02458 |
| **5** | Asteraceae | *Erigeron canadensis* | K | 20 | Konstanz, Line-Eid-Str. | 47.67347 | 9.1538 |
| **6** |  |  | P | 20 | Potsdam-Golm, 14476, Am Urnenfeld | 52.40306 | 12.97156 |
| **7** |  | *Erigeron annuus* | K | 17 | Konstanz, Botanical Garden | 47.69225 | 9.177502 |
| **8** |  |  | P | 10 | Potsdam, 14473, Babelsberger Str. 24 | 52.3923 | 13.06947 |
| **9** |  | *Lactuca serriola* | K | 18 | Tägerwilen (CH), Bahnstrasse | 47.65978 | 9.132178 |
| **10** |  |  | P | 20 | Potsdam-Bornim, 14469, Esplanade | 52.41968 | 13.05278 |
| **11** |  | *Senecio vulgaris* | K | 18 | Reichenau, Berggaessle | 47.68695 | 9.072934 |
| **12** |  |  | P | 11 | Potsdam, 14469, Maulbeerallee 2, Botanical Garden | 52.40367 | 13.0241 |
| **13** |  | *Sonchus oleraceus* | K | 13 | Konstanz, fallow land in the industrial area | 47.67347 | 9.1538 |
| **14** |  |  | P | 10 | Potsdam-Bornim, 14469, Esplanade | 52.41968 | 13.05278 |
| **15** |  | *Tripleurospermum inodorum* | K | 17 | Konstanz, Entsorgungsbetriebe (municipal waste disposal company) | 47.67964 | 9.139306 |
| **16** |  |  | P | 20 | Potsdam-Golm, 14471, Kuhforter Damm | 52.39775 | 12.97966 |
| **17** | Plantaginaceae | *Veronica persica* | K | 17 | Konstanz, Litzelstetten (Oberdorf) | 47.72857 | 9.161455 |
| **18** |  |  | P | 16 | Potsdam, 14469, Am Drachenberg | 52.40833 | 13.01942 |
| **19** |  | *Plantago major* | K | 17 | Konstanz, University | 47.69106 | 9.187332 |
| **20** |  |  | P | 18 | Potsdam, 14469, Am Drachenberg | 52.40811 | 13.02132 |
| **21** | Solanaceae | *Datura stramonium* | K | 15 | Twielfeld, Singen | 47.76201 | 8.802124 |
| **22** |  |  | P | 19 | Schwielowsee-Geltow, 14548, Am Wasser | 52.36363 | 12.96103 |
| **23** |  | *Solanum nigrum* | K | 21 | Konstanz, Litzelstetten | 47.70683 | 9.166862 |
| **24** |  |  | P | 9 | Potsdam, 14469, Parc Sanssouci | 52.40291 | 13.02453 |

**Table S2** Notes on species-specific treatments (pre-sowing treatment + sowing date [day.month.year]). Species names of the natives are in black, and the ones of the non-natives are in red.

| **Plant family** | **Species** | **Status** | **Pre-sowing treatment** | **Sowing date** |
| --- | --- | --- | --- | --- |
| Amaranthaceae | *Amaranthus retroflexus* | non-native | ----------- | 22.04.2016 (Konstanz)  22.04.2016 (Potsdam) |
|  | *Chenopodium album* | native | Chemical scarification  (3 min in 96 % H_2_SO_4_) | 22.04.2016 (Konstanz)  24.04.2016 (Potsdam) |
| Asteraceae | *Erigeron canadensis* | non-native | ----------- | 20.04.2016 (Konstanz)  22.04.2016 (Potsdam) |
|  | *Erigeron annuus* | non-native | ------------ | 20.04.2016 (Konstanz)  22.04.2016 (Potsdam) |
|  | *Lactuca serriola* | native | ----------- | 24.04.2016 (Konstanz)  26.04.2016 (Potsdam) |
|  | *Senecio vulgaris* | native | ----------- | 24.04.2016 (Konstanz)  26.04.2016 (Potsdam) |
|  | *Sonchus oleraceus* | native | ----------- | 20.04.2016 (Konstanz)  22.04.2016 (Potsdam) |
|  | *Tripleurospermum inodorum* | native | ----------- | 24.04.2016 (Konstanz)  26.04.2016 (Potsdam) |
| Plantaginaceae | *Veronica persica* | non-native | ------------ | 18.04.2016 (Konstanz)  19.04.2016 (Potsdam) |
|  | *Plantago major* | native | Soaked in ddH_2_O for 48 h | 20.04.2016 (Konstanz)  21.04.2016 (Potsdam) |
| Solanaceae | *Datura stramonium* | non-native | Soaked in ddH_2_O for 24 h | 19.04.2016 (Konstanz)  20.04.2016 (Potsdam) |
|  | *Solanum nigrum* | native | Soaked in ddH_2_O for 24 h | 19.04.2016 (Konstanz)  20.04.2016 (Potsdam) |

**Table S3** Notes on time-line of the experiment (transplanting, planting and harvesting) per species and region. Dates are in the format day.month.year. Species names of the natives are in black, and the ones of the non-natives are in red.

| **Plant family** | **Species** | **Status** | **Transplanting**  **date** | **Planting date** | **Harvesting date** |
| --- | --- | --- | --- | --- | --- |
| Amaranthaceae | *Amaranthus*  *retroflexus* | non-native | 27.-30.04.2016  (Konstanz)  01.-04.05.2016  (Potsdam) | 17.-25.05.2016 (Konstanz)  05.-13.06.2016 (Potsdam) | 23.-30.06.2016  (Konstanz)  21.07-11.08.2016  (Potsdam) |
|  | *Chenopodium*  *album* | native | 27.-30.04.2016  (Konstanz)  01.-04.05.2016  (Potsdam) | 17.-25.05.2016 (Konstanz)  05.-13.06.2016 (Potsdam) | 20.-23.06.2016  (Konstanz)  03.-17.8.2016  (Potsdam) |
| Asteraceae | *Erigeron*  *canadensis* | non-native | 26.-30.04.2016  (Konstanz)  03.05.2016  (Potsdam) | 20-25.05.2016 (Konstanz)  05.-13.06.2016 (Potsdam) | 07.10.-28.10.2016  (Konstanz)  13.-27.10.2016  (Potsdam) |
|  | *Erigeron*  *annuus* | non-native | 27.-29.04.2016  (Konstanz)  01.-03.05.2016  (Potsdam) | 20-25.05.2016 (Konstanz)  05.-13.06.2016 (Potsdam) | 02.-06.09.2015  (Konstanz)  04.-19.10.2016  (Potsdam) |
|  | *Lactuca*  *serriola* | native | 28.-29.04.2016  (Konstanz)  01.-04.05.2016  (Potsdam) | 17.-25.05.2016 (Konstanz)  05.-13.06.2016 (Potsdam) | 16.-31.10.2016  (Konstanz)  27.09.-19.10.2016  (Potsdam) |
|  | *Senecio*  *vulgaris* | native | 28.04.-04.05.2016  (Konstanz)  01.-13.05.2016  (Potsdam) | 17.-25.05.2016 (Konstanz)  05.-13.06.2016 (Potsdam) | 07.-10.06.2016  (Konstanz)  18.07-01.08.2016  Potsdam) |
|  | *Sonchus*  *oleraceus* | native | 26.04.-02.05.2016  (Konstanz)  01.-13.05.2016  (Potsdam) | 17.-25.05.2016 (Konstanz)  05.-13.06.2016 (Potsdam) | 28.06.-07.07.2016  (Konstanz)  07.-21.07.2016  (Potsdam) |
|  | *Tripleurospermum inodorum* | native | 28.-29.04.2016  (Konstanz)  01.-04.5.2016  (Potsdam) | 17.-25.05.2016 (Konstanz)  05.-13.06.2016 (Potsdam) | 09.08.-08.09.2016  (Konstanz)  29.08.-14.09.2016  (Potsdam) |

*(Table S3 continued from previous page.)*

| **Plant family** | **Species** | **Status** | **Transplanting**  **date** | **Planting date** | **Harvesting date** |
| --- | --- | --- | --- | --- | --- |
| Plantaginaceae | *Veronica*  *persica* | non-native | 28.04.2016  (Konstanz)  01.-03.05.2016  (Potsdam) | 17.-25.05.2016 (Konstanz)  05.-13.06.2016 (Potsdam) | 18.-26.07.2016  (Konstanz)  03.08.-06.09.2016  (Potsdam) |
|  | *Plantago*  *major* | native | 27.04.-08.05.2016  (Konstanz)  04.05.2016  (Potsdam) | 17.-25.05.2016 (Konstanz)  05.-13.06.2016 (Potsdam) | 21.07.-23.08.2016  (Konstanz)  21.07.-09.08.2016  (Potsdam) |
| Solanaceae | *Datura*  *stramonium* | non-native | 29.04.-08.05.2016  (Konstanz)  03.-13.05.2016  (Potsdam) | 17.-25.05.2016 (Konstanz)  05.-13.06.2016 (Potsdam) | 01.-04.08.2016  (Konstanz)  22.08.-05.09.2016  (Potsdam) |
|  | *Solanum*  *nigrum* | native | 29.-30.04.2016  (Konstanz)  01.-03.05.2016  (Potsdam) | 17.-25.05.2016 (Konstanz)  05.-13.06.2016 (Potsdam) | 28.07.-01.08.2016  (Konstanz)  16.08-01.09.2016  (Potsdam) |

**Table S4** Description of the field sites in the Konstanz region (black) and the Potsdam region (blue).

| **Site ID** | **Site** | **Latitude [°]** | **Longitude [°]** | **Address** | **Environmental**  **gradient** | **Plant cover**  **at start**  **[%]** | **Plant cover at end**  **[%]** | **Plant cover forbs [%]^a^** | **Plant cover grasses [%]^a^** |
| --- | --- | --- | --- | --- | --- | --- | --- | --- | --- |
| **MQ** | Potsdam-  Marquardt | 52.46647 | 12.95647 | Hauptstrasse 36B,  14476 Potsdam | Soil moisture  (lower part of field contrastingly wetter) | 0 | 100 | 70 | 30 |
| **DB** | Potsdam/  Botanical  Garden | 52.40769 | 13.0224 | Eichenallee 36,  14469 Potsdam | Shading | 0 | 100 | 90 | 10 |
| **GR** | Ludwigsfelde/Gröben | 52.28219 | 13.16803 | Uppstallweg 1,  14974 Ludwigsfelde | Shading | 0 | 25 | 20 | 80 |
| **Boga** | Konstanz/  Botanical  Garden | 47.69216 | 9.179243 | Langhardtstraße,  78464 Konstanz | Soil moisture  (upper part of field slightly drier than  lower part) | ~1 | 100 | 30 | 50 |
| **Hättli** | Konstanz/  Hättelihof | 47.68384 | 9.19343 | Universitätsstraße10, 78464 Konstanz | Soil moisture | ~2 | 75 | 35 | 40 |
| **Uni** | Konstanz/  University | 47.69081 | 9.186349 | Mainaustraße 185,  78464 Konstanz | Shading  (e.g. only full sun exposure 9:00 -17:00, Mid-August),  soil moisture | ~5-10 | 100 | 55 | 10 |

^a^Plant cover of forbs and grasses was scored in August 2016 (i.e. at the end of the growing season).

**Table S5** Results of generalized linear mixed models for survival. Analysis followed the prior specification by Bolker and Hadfield (see Methods S1) for the analysis of datasets with quasi- or complete separation (i.e. complete 0s or 1s in one of the subgroups). We accounted for the random effects of maternal lines and blocks nested within field sites. Significant *P*-values at the *P* < 0.05 threshold are marked in bold. CI is the 95% credible interval. Species names of the natives are in black, and the ones of the non-natives are in red.

| **Species** | ***Amaranthus retroflexus*** | | | | | ***Chenopodium album*** | | | | | ***Veronica persica*** | | | |
| --- | --- | --- | --- | --- | --- | --- | --- | --- | --- | --- | --- | --- | --- | --- |
| **Sample size n** | 214 plants (95.3% surviving) | | | | | 282 plants (87.9% surviving) | | | | | 321 plants (73.2% surviving) | | | |
| **Ratio of non-event to event** | 10 dead | | 204 surviving | | | 34 dead | | 248 surviving | | | 86 dead | | 235 surviving | |
| **Status** | non-native | | | | | native | | | | | non-native | | | |
| **Plant family** | Amaranthaceae | | | | | | | | | | Plantaginaceae | | | |
| **Moderators** | Posterior mean | L-95%  CI | | U-95% CI | *P*MCMC | Posterior mean | L-95%  CI | | U-95% CI | *P*MCMC | Posterior mean | L-95%  CI | U-95% CI | *P*MCMC |
| **Intercept** | **3.98** | **1.74** | | **6.45** | **0.009** | 4.15 | -0.39 | | 8.17 | 0.086 | 2.59 | -2.56 | 7.25 | 0.300 |
| **Region [R]** | 0.48 | -2.39 | | 3.89 | 0.784 | -1.13 | -5.37 | | 3.70 | 0.581 | -1.25 | -6.42 | 4.04 | 0.627 |
| **Zebularine treatment [Z]** | -0.50 | -2.32 | | 1.34 | 0.589 | -1.42 | -4.07 | | 1.30 | 0.281 | -1.56 | -4.41 | 1.15 | 0.257 |
| **Origin [O]** | 3.12 | -0.37 | | 6.70 | 0.062 | -0.54 | -3.58 | | 2.30 | 0.707 | -0.23 | -3.11 | 2.64 | 0.878 |
| **R×Z** | 0.13 | -3.00 | | 3.26 | 0.935 | 1.09 | -1.70 | | 3.95 | 0.462 | 1.45 | -1.49 | 4.45 | 0.335 |
| **R×O** | -1.29 | -5.49 | | 2.88 | 0.534 | -0.68 | -3.74 | | 2.43 | 0.663 | 0.86 | -2.10 | 3.81 | 0.583 |
| **Z×O** | 0.07 | -3.86 | | 4.09 | 0.977 | -1.34 | -4.52 | | 1.51 | 0.392 | -2.78 | -5.83 | 0.08 | 0.063 |
| **R×Z×O** | -2.52 | -7.11 | | 1.79 | 0.260 | 2.32 | -1.18 | | 5.84 | 0.200 | 1.59 | -1.62 | 4.84 | 0.328 |

*(Table S5 continued from previous page).*

| **Species** | ***Plantago major*** | | | | | ***Datura stramonium*** | | | | | ***Solanum nigrum*** | | | |
| --- | --- | --- | --- | --- | --- | --- | --- | --- | --- | --- | --- | --- | --- | --- |
| **Sample size n** | 324 plants (88.9% surviving) | | | | | 282 plants (80.9% surviving) | | | | | 356 plants (92.7% surviving) | | | |
| **Ratio of non-event to event** | 36 dead | | 288 surviving | | | 54 dead | | 228 surviving | | | 26 dead | | 330 surviving | |
| **Status** | native | | | | | non-native | | | | | native | | | |
| **Plant family** | Plantaginaceae | | | | | Solanaceae | | | | | | | | |
| **Moderators** | Posterior mean | L-95%  CI | | U-95% CI | *P*MCMC | Posterior mean | L-95%  CI | | U-95% CI | *P*MCMC | Posterior mean | L-95%  CI | U-95% CI | *P*MCMC |
| **Intercept** | **4.49** | **1.50** | | **7.40** | **0.016** | **3.27** | **0.61** | | **5.76** | **0.033** | **4.78** | **1.27** | **8.26** | **0.032** |
| **Region [R]** | -0.65 | -3.96 | | 2.84 | 0.658 | -0.72 | -3.88 | | 2.58 | 0.601 | -1.94 | -5.72 | 2.14 | 0.308 |
| **Zebularine treatment [Z]** | -0.11 | -2.24 | | 2.18 | 0.916 | 0.19 | -1.59 | | 2.05 | 0.849 | -0.48 | -2.96 | 2.15 | 0.720 |
| **Origin [O]** | -0.24 | -2.53 | | 2.06 | 0.826 | **-3.46** | **-5.20** | | **-1.84** | **<1e-04** | 0.65 | -2.31 | 3.64 | 0.680 |
| **R×Z** | -1.70 | -4.30 | | 0.66 | 0.171 | -0.38 | -2.51 | | 1.69 | 0.728 | 0.98 | -1.80 | 3.63 | 0.482 |
| **R×O** | -1.47 | -4.09 | | 1.03 | 0.251 | **2.92** | **0.92** | | **4.91** | **0.004** | -0.06 | -3.17 | 3.02 | 0.986 |
| **Z×O** | 0.55 | -2.39 | | 3.52 | 0.720 | 0.45 | -1.65 | | 2.68 | 0.692 | -1.24 | -4.63 | 1.89 | 0.450 |
| **R×Z×O** | 1.58 | -1.62 | | 4.77 | 0.329 | -0.58 | -3.22 | | 2.02 | 0.674 | 0.24 | -3.27 | 3.61 | 0.899 |

*(Table S5 continued from previous page).*

| **Species** | ***Erigeron canadensis*** | | | | | ***Erigeron annuus*** | | | | | ***Lactuca serriola*** | | | |
| --- | --- | --- | --- | --- | --- | --- | --- | --- | --- | --- | --- | --- | --- | --- |
| **Sample size n** | 313 plants (75.1% surviving) | | | | | 317 plants (80.1% surviving) | | | | | 427 plants (42.9% surviving) | | | |
| **Ratio of non-event to event** | 78 dead | | 235 surviving | | | 63 dead | | 254 surviving | | | 244 dead | | 183 surviving | |
| **Status** | non-native | | | | | non-native | | | | | native | | | |
| **Plant family** | Asteraceae | | | | | | | | | | | | | |
| **Moderators** | Posterior mean | L-95%  CI | | U-95% CI | *P*MCMC | Posterior mean | L-95%  CI | | U-95% CI | *P*MCMC | Posterior mean | L-95%  CI | U-95% CI | *P*MCMC |
| **Intercept** | 0.97 | -4.37 | | 6.35 | 0.712 | 1.50 | -3.74 | | 6.18 | 0.530 | -0.55 | -3.67 | 2.35 | 0.699 |
| **Region [R]** | -0.63 | -6.18 | | 4.91 | 0.822 | -0.71 | -5.97 | | 4.48 | 0.777 | -2.05 | -5.93 | 1.80 | 0.296 |
| **Zebularine treatment [Z]** | 0.69 | -1.24 | | 2.61 | 0.479 | -0.33 | -2.37 | | 1.71 | 0.749 | -0.54 | -1.59 | 0.46 | 0.313 |
| **Origin [O]** | 0.25 | -2.84 | | 3.14 | 0.856 | 0.76 | -1.65 | | 3.13 | 0.532 | 1.06 | -0.09 | 2.17 | 0.058 |
| **R×Z** | 0.73 | -1.82 | | 3.50 | 0.595 | -1.68 | -4.54 | | 0.92 | 0.223 | **1.97** | **0.29** | **3.77** | **0.024** |
| **R×O** | -0.74 | -4.88 | | 3.68 | 0.737 | -1.16 | -3.95 | | 1.87 | 0.432 | **1.94** | **0.09** | **3.70** | **0.033** |
| **Z×O** | **-4.12** | **-7.28** | | **-1.07** | **0.005** | 0.66 | -2.34 | | 3.53 | 0.666 | -0.77 | -2.20 | 0.68 | 0.291 |
| **R×Z×O** | 3.39 | -0.94 | | 7.80 | 0.131 | 0.79 | -2.63 | | 4.29 | 0.650 | -0.98 | -3.24 | 1.26 | 0.389 |

*(Table S5 continued from previous page).*

| **Species** | ***Senecio vulgaris*** | | | | | ***Sonchus oleraceus*** | | | | | ***Tripleurospermum inodorum*** | | | |
| --- | --- | --- | --- | --- | --- | --- | --- | --- | --- | --- | --- | --- | --- | --- |
| **Sample size n** | 287 plants (97.9% surviving) | | | | | 208 plants (96.2% surviving) | | | | | 398 plants (78.4% surviving) | | | |
| **Ratio of non-event to event** | 6 dead | | 281 surviving | | | 8 dead | | 200 surviving | | | 86 dead | | 312 surviving | |
| **Status** | native | | | | | native | | | | | native | | | |
| **Plant family** | Asteraceae | | | | | | | | | | | | | |
| **Moderators** | Posterior mean | L-95%  CI | | U-95% CI | *P*MCMC | Posterior mean | L-95%  CI | | U-95% CI | *P*MCMC | Posterior mean | L-95%  CI | U-95% CI | *P*MCMC |
| **Intercept** | 3.97 | -2.44 | | 9.42 | 0.235 | 3.37 | -3.33 | | 9.33 | 0.350 | 3.61 | -0.54 | 7.26 | 0.096 |
| **Region [R]** | 0.51 | -4.50 | | 5.69 | 0.869 | 0.51 | -4.94 | | 5.98 | 0.860 | -1.84 | -6.19 | 2.82 | 0.400 |
| **Zebularine treatment [Z]** | 1.62 | -1.72 | | 5.26 | 0.361 | -0.22 | -4.49 | | 3.91 | 0.928 | 1.24 | -1.16 | 3.82 | 0.306 |
| **Origin [O]** | -0.45 | -4.18 | | 3.55 | 0.802 | 0.00 | -4.58 | | 4.56 | 0.993 | 0.52 | -1.70 | 2.61 | 0.638 |
| **R×Z** | -1.68 | -5.47 | | 2.02 | 0.376 | -1.59 | -5.82 | | 2.59 | 0.456 | -1.49 | -4.20 | 1.03 | 0.249 |
| **R×O** | 1.29 | -3.13 | | 5.75 | 0.557 | -0.89 | -5.42 | | 3.65 | 0.694 | -1.26 | -3.61 | 1.16 | 0.300 |
| **Z×O** | 0.32 | -4.10 | | 4.27 | 0.883 | 0.78 | -3.78 | | 5.38 | 0.740 | -1.34 | -4.15 | 1.48 | 0.352 |
| **R×Z×O** | -1.55 | -6.23 | | 2.94 | 0.505 | 0.37 | -4.33 | | 4.86 | 0.867 | 1.51 | -1.51 | 4.58 | 0.323 |

**Table S6** Results of linear mixed models for complete aboveground biomass. Data was power transformed to fulfil assumption of normality and homogeneity of variance in the residuals for the linear mixed models. We accounted for the random effects of maternal lines and blocks nested within field sites. Significant P-values at the P < 0.05 threshold are marked in bold. Species names of the natives are in black, and the ones of the non-natives are in red.

| **Species** | ***Amaranthus retroflexus*** | | ***Chenopodium album*** | | | ***Veronica persica*** | | ***Plantago major*** | | | ***Datura stramonium*** | | ***Solanum nigrum*** | |
| --- | --- | --- | --- | --- | --- | --- | --- | --- | --- | --- | --- | --- | --- | --- |
| **No. of fields** | 6 | | 6 | | | 6 | | 6 | | | 6 | | 6 | |
| **No. of blocks** | 18 | | 18 | | | 16 | | 18 | | | 18 | | 18 | |
| **Maternal lines** | 27 | | 30 | | | 31 | | 33 | | | 28 | | 30 | |
| **Sample size n** | 204 plants | | 244 plants | | | 231 plants | | 281 plants | | | 216 plants | | 327 plants | |
| **Data trans-formation (y^x^)** | x=0.1 | | x=0.1 | | | x=0.1 | | x=0.1 | | | x=0.1 | | x=0.1 | |
| **Status** | non-native | | native | | | non-native | | native | | | non-native | | native | |
| **Plant family** | Amaranthaceae | | | | | Plantaginaceae | | | | | Solanaceae | | | |
| **Moderators** | χ^2^  (d.f. = 1) | *P* | χ^2^  (d.f. = 1) | *P* | χ^2^  (d.f. = 1) | | *P* | | χ^2^  (d.f. = 1) | *P* | χ^2^  (d.f. = 1) | *P* | χ^2^  (d.f. = 1) | *P* |
| **Region [R]** | **4.05** | **0.044** | **7.07** | **0.008** | 0.01 | | 0.924 | | **3.86** | **0.049** | **6.39** | **0.011** | 3.30 | 0.069 |
| **Zebularine treatment [Z]** | 2.55 | 0.111 | **5.87** | **0.015** | **15.66** | | **<1e-03** | | **12.25** | **<1e-03** | 0.31 | 0.575 | 2.83 | 0.093 |
| **Origin [O]** | 1.33 | 0.249 | 0.58 | 0.447 | **5.41** | | **0.020** | | 1.22 | 0.269 | **11.65** | **<1e-03** | **11.01** | **<1e-03** |
| **R×Z** | 2.14 | 0.143 | **4.21** | **0.040** | **3.98** | | **0.046** | | 0.35 | 0.555 | 0.11 | 0.741 | 1.50 | 0.220 |
| **R×O** | **5.65** | **0.017** | 3.68 | 0.055 | 0.56 | | 0.455 | | 0.00 | 0.963 | **5.59** | **0.018** | 2.55 | 0.110 |
| **Z×O** | 1.75 | 0.186 | 0.04 | 0.841 | **12.64** | | **<1e-03** | | **7.01** | **0.008** | 0.06 | 0.800 | 1.07 | 0.301 |
| **R×Z×O** | 0.73 | 0.394 | 0.72 | 0.396 | 3.41 | | 0.065 | | 1.81 | 0.178 | 0.67 | 0.413 | 1.41 | 0.234 |

*(Table S6 continued from previous page).*

| **Species** | ***Erigeron***  ***canadensis*** | | ***Erigeron annuus*** | | ***Lactuca serriola*** | | | ***Senecio vulgaris*** | | ***Sonchus oleraceus*** | | ***Tripleurospermum inodorum*** | |
| --- | --- | --- | --- | --- | --- | --- | --- | --- | --- | --- | --- | --- | --- |
| **No. of fields** | 5 | | 5 | | 6 | | | 6 | | 6 | | 6 | |
| **No. of blocks** | 15 | | 15 | | 16 | | | 18 | | 18 | | 17 | |
| **Maternal lines** | 38 | | 35 | | 38 | | | 29 | | 19 | | 34 | |
| **Sample size n** | 233 plants | | 250 plants | | 182 plants | | | 279 plants | | 197 plants | | 307 plants | |
| **Data trans-formation (y^x^)** | x=0.1 | | x=0.1 | | x=0.1 | | | x=0.1 | | x=0.1 | | x=0.1 | |
| **Status** | non-native | | non-native | | native | | | native | | native | | native | |
| **Plant family** | Asteraceae | | | | | | | | | | | | |
| **Moderators** | χ^2^  (d.f. = 1) | *P* | χ^2^  (d.f. = 1) | *P* | χ^2^  (d.f. = 1) | *P* | | χ^2^  (d.f. = 1) | *P* | χ^2^  (d.f. = 1) | *P* | χ^2^  (d.f. = 1) | *P* |
| **Region [R]** | 0.10 | 0.750 | 0.43 | 0.510 | 0.29 | | 0.589 | 0.34 | 0.562 | 0.38 | 0.540 | 0.10 | 0.752 |
| **Zebularine treatment [Z]** | 1.57 | 0.211 | **6.48** | **0.011** | **10.93** | | **<1e-03** | **26.71** | **<1e-06** | **5.78** | **0.016** | **9.70** | **0.002** |
| **Origin [O]** | 0.15 | 0.696 | **4.39** | **0.036** | **7.73** | | **0.005** | **5.67** | **0.017** | 1.66 | 0.197 | 0.92 | 0.337 |
| **R×Z** | 1.01 | 0.315 | 0.08 | 0.775 | 0.11 | | 0.743 | **18.23** | **<1e-04** | 0.12 | 0.734 | **14.62** | **<1e-03** |
| **R×O** | 1.58 | 0.209 | **10.84** | **<1e-03** | **6.87** | | **0.009** | **4.46** | **0.035** | 0.05 | 0.830 | 0.17 | 0.678 |
| **Z×O** | 0.32 | 0.575 | 0.71 | 0.398 | 0.60 | | 0.438 | 0.82 | 0.366 | 1.10 | 0.295 | 0.00 | 0.994 |
| **R×Z×O** | NA | NA | 2.23 | 0.136 | 0.00 | | 0.997 | 0.09 | 0.763 | 0.18 | 0.675 | 1.01 | 0.314 |

**Table S7** Results of generalized linear mixed models for flowering probability. Analysis followed the prior specification by Bolker and Hadfield (see Methods S1) for the analysis of datasets with quasi- or complete separation (i.e. complete 0s or 1s in one of the subgroups). We accounted for the random effects of maternal lines and blocks nested within field sites. CI is the 95% credible interval. Significant *P*-values at the *P* < 0.05. Species names of the natives are in black, and the ones of the non-natives are in red.

| **Species** | ***Amaranthus retroflexus*** | | | | ***Chenopodium album*** | | | | ***Veronica persica*** | | | |
| --- | --- | --- | --- | --- | --- | --- | --- | --- | --- | --- | --- | --- |
| **Sample size n** | 204 plants (94.1% flowering) | | | | 248 plants (89.9% flowering) | | | | 235 plants (95.3% flowering) | | | |
| **Ratio of non-event to event** | 12 non-flowering | | 192 flowering | | 25 non-flowering | | 223 flowering | | 11 non-flowering | | 224 flowering | |
| **Status** | non-native | | | | native | | | | non-native | | | |
| **Plant family** | Amaranthaceae | | | | | | | | Plantaginaceae | | | |
| **Moderators** | Posterior mean | L-95%  CI | U-95% CI | *P*MCMC | Posterior mean | L-95%  CI | U-95% CI | *P*MCMC | Posterior mean | L-95%  CI | U-95% CI | *P*MCMC |
| **Intercept** | **2.67** | **0.14** | **5.26** | **0.047** | 3.30 | -0.67 | 7.19 | 0.108 | 3.65 | -1.35 | 7.78 | 0.133 |
| **Region [R]** | 1.70 | -1.75 | 5.28 | 0.310 | 0.99 | -3.28 | 5.50 | 0.663 | -0.14 | -4.70 | 4.66 | 0.925 |
| **Zebularine treatment [Z]** | 1.52 | -0.26 | 3.36 | 0.075 | -1.40 | -3.12 | 0.22 | 0.092 | -0.59 | -2.62 | 1.49 | 0.578 |
| **Origin [O]** | **2.71** | **-0.08** | **5.39** | **0.030** | 3.10 | -0.10 | 6.61 | 0.059 | 2.67 | -0.31 | 5.98 | 0.076 |
| **R×Z** | -1.00 | -4.44 | 2.26 | 0.556 | 1.13 | -1.41 | 3.69 | 0.386 | -1.30 | -4.36 | 1.64 | 0.399 |
| **R×O** | **-4.08** | **-7.81** | **-0.26** | **0.035** | -0.53 | -4.81 | 3.72 | 0.789 | -1.50 | -5.29 | 2.18 | 0.428 |
| **Z×O** | 0.82 | -3.13 | 4.90 | 0.706 | -1.75 | -5.15 | 1.56 | 0.297 | 1.33 | -2.80 | 5.21 | 0.524 |
| **R×Z×O** | -0.65 | -5.10 | 3.69 | 0.790 | -1.74 | -6.25 | 2.97 | 0.460 | -0.21 | -4.51 | 4.13 | 0.942 |

*(Table S7 continued from previous page).*

| **Species** | ***Plantago major*** | | | | ***Datura stramonium*** | | | | ***Solanum nigrum*** | | | |
| --- | --- | --- | --- | --- | --- | --- | --- | --- | --- | --- | --- | --- |
| **Sample size n** | 283 plants (93.6% flowering) | | | | 213 plants (81.7% flowering) | | | | 327 plants (97.4% flowering) | | | |
| **Ratio of non-event to event** | 18 non-flowering | | 265 flowering | | 39 non-flowering | | 174 flowering | | 9 non-flowering | | 318 flowering | |
| **Status** | native | | | | non-native | | | | native | | | |
| **Plant family** | Plantaginaceae | | | | Solanaceae | | | | | | | |
| **Moderators** | Posterior mean | L-95%  CI | U-95% CI | *P*MCMC | Posterior mean | L-95%  CI | U-95% CI | *P*MCMC | Posterior mean | L-95%  CI | U-95% CI | *P*MCMC |
| **Intercept** | 4.96 | -0.42 | 9.50 | 0.095 | **1.83** | **0.35** | **3.70** | **0.046** | **5.38** | **1.05** | **9.00** | **0.038** |
| **Region [R]** | -1.40 | -5.71 | 3.64 | 0.510 | 0.49 | -1.74 | 2.71 | 0.644 | -1.16 | -4.85 | 3.07 | 0.470 |
| **Zebularine treatment [Z]** | -0.52 | -3.62 | 2.43 | 0.742 | 0.28 | -0.99 | 1.61 | 0.692 | -0.02 | -2.80 | 2.89 | 0.991 |
| **Origin [O]** | 2.68 | -0.91 | 6.95 | 0.168 | -1.00 | -2.71 | 0.76 | 0.278 | 2.59 | -1.18 | 6.71 | 0.187 |
| **R×Z** | -0.68 | -3.94 | 2.60 | 0.659 | 0.65 | -1.27 | 3.07 | 0.546 | 0.74 | -2.43 | 3.91 | 0.643 |
| **R×O** | -0.19 | -4.11 | 3.69 | 0.936 | **3.49** | **0.76** | **6.30** | **0.008** | 0.17 | -4.03 | 4.24 | 0.934 |
| **Z×O** | -0.16 | -4.30 | 3.78 | 0.929 | -0.73 | -2.80 | 1.40 | 0.508 | -0.86 | -5.04 | 3.40 | 0.676 |
| **R×Z×O** | -1.90 | -6.11 | 2.29 | 0.384 | -3.24 | -6.52 | 0.26 | 0.052 | -2.51 | -6.88 | 1.70 | 0.254 |

*(Table S7 continued from previous page).*

| **Species** | ***Erigeron canadensis*** | | | | ***Erigeron annuus*** | | | | ***Lactuca serriola*** | | | |
| --- | --- | --- | --- | --- | --- | --- | --- | --- | --- | --- | --- | --- |
| **Sample size n** | 235 plants (50.6% flowering) | | | | 254 plants (44.9% flowering) | | | | 181 plants (11.1% flowering) | | | |
| **Ratio of non-event to event** | 116 non-flowering | | 119 flowering | | 140 non-flowering | | 114 flowering | | 161 non-flowering | | 20 flowering | |
| **Status** | non-native | | | | non-native | | | | native | | | |
| **Plant family** | Asteraceae | | | | | | | | | | | |
| **Moderators** | Posterior mean | L-95%  CI | U-95% CI | *P*MCMC | Posterior mean | L-95%  CI | U-95% CI | *P*MCMC | Posterior mean | L-95%  CI | U-95% CI | *P*MCMC |
| **Intercept** | -0.19 | -4.45 | 3.56 | 0.930 | -0.70 | -3.87 | 2.64 | 0.631 | -3.68 | -7.60 | 0.69 | 0.084 |
| **Region [R]** | -1.02 | -5.76 | 3.81 | 0.674 | -0.36 | -4.61 | 3.90 | 0.864 | -0.21 | -4.70 | 4.20 | 0.936 |
| **Zebularine treatment [Z]** | 0.74 | -0.30 | 1.85 | 0.177 | -0.11 | -1.20 | 1.00 | 0.843 | -0.88 | -3.38 | 1.66 | 0.494 |
| **Origin [O]** | -0.95 | -2.51 | 0.56 | 0.219 | **1.72** | **0.59** | **3.03** | **0.004** | -1.75 | -5.04 | 1.32 | 0.269 |
| **R×Z** | -0.80 | -3.21 | 1.74 | 0.516 | -0.18 | -2.61 | 2.18 | 0.872 | -1.49 | -4.81 | 2.27 | 0.405 |
| **R×O** | 2.92 | -0.92 | 6.93 | 0.140 | -1.81 | -3.98 | 0.51 | 0.114 | 0.78 | -3.04 | 4.36 | 0.658 |
| **Z×O** | **-2.26** | **-3.94** | **-0.51** | **0.007** | -0.72 | -2.27 | 0.75 | 0.363 | 1.79 | -1.29 | 4.84 | 0.243 |
| **R×Z×O** | NA | NA | NA | NA | 0.76 | -2.17 | 3.75 | 0.615 | -1.58 | -5.35 | 2.46 | 0.433 |

*(Table S7 continued from previous page).*

| **Species** | ***Senecio vulgaris*** | | | | ***Sonchus oleraceus*** | | | | | ***Tripleurospermum inodorum*** | | | |
| --- | --- | --- | --- | --- | --- | --- | --- | --- | --- | --- | --- | --- | --- |
| **Sample size n** | 265 plants (95.1% flowering) | | | | 199 plants (76.9% flowering) | | | | | 312 plants (84.0% flowering) | | | |
| **Ratio of non-event to event** | 13 non-flowering | | 252 flowering | | 46 non-flowering | | 153 flowering | | | 50 non-flowering | | 262 flowering | |
| **Status** | native | | | | native | | | | | native | | | |
| **Plant family** | Asteraceae | | | | | | | | | | | | |
| **Moderators** | Posterior mean | L-95%  CI | U-95% CI | *P*MCMC | Posterior mean | L-95%  CI | U-95% CI | *P*MCMC | Posterior mean | | L-95%  CI | U-95% CI | *P*MCMC |
| **Intercept** | **4.71** | **1.27** | **7.96** | **0.032** | **3.65** | **0.72** | **6.31** | **0.014** | | 2.74 | 0.20 | 5.51 | 0.056 |
| **Region [R]** | -0.82 | -4.16 | 3.10 | 0.575 | 0.10 | -2.64 | 3.10 | 0.956 | | 0.55 | -2.48 | 3.95 | 0.750 |
| **Zebularine treatment [Z]** | -0.52 | -2.72 | 1.70 | 0.641 | **-1.76** | **-3.69** | **-0.01** | **0.048** | | -0.81 | -2.19 | 0.39 | 0.234 |
| **Origin [O]** | 1.56 | -1.76 | 4.81 | 0.357 | -1.77 | -5.22 | 1.95 | 0.312 | | -0.61 | -2.12 | 0.74 | 0.396 |
| **R×Z** | -0.96 | -3.52 | 1.70 | 0.459 | -1.57 | -4.14 | 0.76 | 0.212 | | -0.32 | -2.30 | 1.57 | 0.742 |
| **R×O** | -1.67 | -5.50 | 2.18 | 0.397 | 0.49 | -2.25 | 3.20 | 0.725 | | 1.19 | -1.15 | 3.66 | 0.344 |
| **Z×O** | 2.60 | -1.67 | 7.12 | 0.252 | -0.31 | -2.86 | 2.25 | 0.806 | | 1.55 | -0.65 | 3.25 | 0.116 |
| **R×Z×O** | 1.63 | -2.97 | 6.86 | 0.535 | 1.38 | -2.00 | 4.56 | 0.414 | | -1.55 | -4.43 | 1.33 | 0.288 |

**Table S8** Results of linear mixed models for reproductive biomass. Data was power transformed to fulfil assumption of normality and homogeneity of variance in the residuals for the linear mixed models. We accounted for the random effects of maternal lines and blocks nested within field sites. Significant *P*-values at the *P* < 0.05 threshold are marked in bold. Species names of the natives are in black, and the ones of the non-natives are in red.

| **Species** | ***Amaranthus retroflexus*** | | ***Chenopodium album*** | | ***Veronica persica*** | | ***Plantago major*** | | ***Datura stramonium*** | | ***Solanum nigrum*** | |
| --- | --- | --- | --- | --- | --- | --- | --- | --- | --- | --- | --- | --- |
| **No. of fields** | 6 | | 6 | | 6 | | 6 | | 6 | | 6 | |
| **No. of blocks** | 18 | | 18 | | 16 | | 18 | | 18 | | 18 | |
| **Maternal lines** | 27 | | 30 | | 31 | | 33 | | 27 | | 30 | |
| **Sample size n** | 192 plants | | 223 plants | | 220 plants | | 258 plants | | 172 plants | | 315 plants | |
| **Data trans-formation (y^x^)** | x=0.1 | | x=0.1 | | x=0.1 | | x=0.1 | | x=0.2 | | x=0.2 | |
| **Status** | non-native | | native | | non-native | | native | | non-native | | native | |
| **Plant family** | Amaranthaceae | | | | Plantaginaceae | | | | Solanaceae | | | |
| **Moderators** | χ2 (d.f. =1) | *P* | χ2 (d.f. =1) | *P* | χ2 (d.f. =1) | *P* | χ2 (d.f.=1) | *P* | χ2 (d.f. =1) | *P* | χ2 (d.f. =1) | *P* |
| **Region [R]** | **4.42** | **0.036** | **11.04** | **<1e-03** | 3.24 | 0.072 | **5.97** | **0.015** | **5.80** | **0.016** | 2.23 | 0.136 |
| **Zebularine treatment [Z]** | 0.17 | 0.681 | **7.98** | **0.005** | **14.99** | **<1e-03** | **7.55** | **0.006** | 1.50 | 0.221 | **5.10** | **0.024** |
| **Origin [O]** | **22.18** | **<1e-05** | **9.98** | **0.002** | **8.44** | **0.004** | 2.21 | 0.137 | 3.13 | 0.077 | **6.26** | **0.012** |
| **R×Z** | 3.37 | 0.066 | 0.17 | 0.680 | 0.57 | 0.451 | 1.15 | 0.283 | 0.01 | 0.919 | 0.88 | 0.347 |
| **R×O** | **12.58** | **<1e-03** | **7.67** | **0.006** | 1.30 | 0.254 | 0.42 | 0.517 | 1.27 | 0.259 | 2.89 | 0.089 |
| **Z×O** | 1.03 | 0.309 | 0.01 | 0.910 | **6.07** | **0.014** | **5.38** | **0.020** | 1.21 | 0.272 | 0.04 | 0.836 |
| **R×Z×O** | 1.69 | 0.194 | 0.49 | 0.486 | 1.39 | 0.238 | 0.41 | 0.521 | 1.73 | 0.188 | 2.84 | 0.092 |

*(Table S8 continued from previous page).*

| **Species** | ***Erigeron***  ***canadensis*** | | ***Erigeron annuus*** | | ***Lactuca serriola*** | | ***Senecio vulgaris*** | | ***Sonchus oleraceus*** | | ***Tripleurospermum inodorum*** | |
| --- | --- | --- | --- | --- | --- | --- | --- | --- | --- | --- | --- | --- |
| **No. of fields** | 4 | | 5 | | 5 | | 6 | | 6 | | 6 | |
| **No. of blocks** | 12 | | 13 | | 9 | | 18 | | 18 | | 17 | |
| **Maternal lines** | 34 | | 34 | | 10 | | 29 | | 17 | | 34 | |
| **Sample size n** | 118 plants | | 112 plants | | 20 plants | | 252 plants | | 152 plants | | 259 plants | |
| **Data trans-formation (y^x^)** | x=0.1 | | x=0.2 | | x=0.1 | | x=0.2 | | x=0.1 | | x=0.2 | |
| **Status** | non-native | | non-native | | native | | native | | native | | native | |
| **Plant family** | Asteraceae | | | | | | | | | | | |
| **Moderators** | χ2 (d.f.=1) | *P* | χ2 (d.f. =1) | *P* | χ2 (d.f. =1) | *P* | χ2 (d.f. =1) | *P* | χ2 (d.f. =1) | *P* | χ2 (d.f. =1) | *P* |
| **Region [R]** | **5.85** | **0.016** | 2.30 | 0.129 | **3.84** | **0.050** | 0.12 | 0.729 | 0.52 | 0.470 | 0.31 | 0.579 |
| **Zebularine treatment [Z]** | **6.84** | **0.009** | 0.41 | 0.523 | 3.24 | 0.072 | **16.10** | **<1e-04** | **5.92** | **0.015** | **4.03** | **0.045** |
| **Origin [O]** | 1.40 | 0.237 | 3.51 | 0.061 | 0.95 | 0.330 | 0.12 | 0.730 | 0.48 | 0.489 | 2.14 | 0.144 |
| **R×Z** | 0.53 | 0.467 | 0.32 | 0.574 | 1.39 | 0.239 | **8.37** | **0.004** | 2.39 | 0.122 | **9.40** | **0.002** |
| **R×O** | **4.78** | **0.029** | **6.06** | **0.014** | 1.00 | 0.317 | **5.68** | **0.017** | 0.00 | 0.977 | 0.10 | 0.753 |
| **Z×O** | 0.59 | 0.442 | 1.29 | 0.256 | 2.66 | 0.103 | 0.09 | 0.764 | 0.01 | 0.929 | 1.91 | 0.167 |
| **R×Z×O** | NA | NA | 1.35 | 0.245 | 1.88 | 0.171 | 0.03 | 0.854 | 0.06 | 0.807 | 1.68 | 0.195 |

**Table S9** Estimates and confidence intervals of survival effect sizes summarized at the region-level, in Fig. S4A). Significant effect sizes at the *P* < 0.05 threshold are marked in bold. K – Konstanz transplant region; P – Potsdam transplant region; CON – control treatment; ZEB – zebularine treatment; predicted – summarized effect size; ci.lb – lower 95% confidence interval; ci.ub – upper 95% confidence interval. Species names of the natives are in black, and the ones of the non-natives are in red.

| **Plant family** | **Status** | **Species** | **Region** | **Treatment** | **predicted** | **ci.lb** | **ci.ub** |
| --- | --- | --- | --- | --- | --- | --- | --- |
| Amaranthaceae | non-native | *Amaranthus*  *retroflexus* | K | CON | -0.724 | -2.928 | 1.480 |
|  |  |  |  | ZEB | -1.093 | -3.270 | 1.085 |
|  |  |  | P | CON | 0.453 | -2.542 | 3.448 |
|  |  |  |  | ZEB | -0.407 | -2.259 | 1.445 |
|  | native | *Chenopodium*  *album* | K | CON | 0.000 | -2.415 | 2.415 |
|  |  |  |  | ZEB | 1.034 | -0.600 | 2.667 |
|  |  |  | P | CON | -1.331 | -3.009 | 0.347 |
|  |  |  |  | ZEB | -0.300 | -2.229 | 1.628 |
| Asteraceae | non-native | *Erigeron*  *canadensis* | K | CON | -0.864 | -2.168 | 0.441 |
|  |  |  |  | ZEB | 0.777 | -0.402 | 1.957 |
|  |  |  | P | CON | -1.513 | -7.330 | 4.303 |
|  |  |  |  | ZEB | 0.303 | -1.836 | 2.441 |
|  |  | *Erigeron annuus* | K | CON | -0.331 | -2.076 | 1.413 |
|  |  |  |  | ZEB | -0.332 | -2.049 | 1.386 |
|  |  |  | P | CON | -0.335 | -2.057 | 1.387 |
|  |  |  |  | ZEB | 0.855 | -0.533 | 2.242 |
|  | native | *Lactuca*  *serriola* | K | CON | -0.467 | -1.487 | 0.552 |
|  |  |  |  | ZEB | -0.148 | -0.894 | 0.598 |
|  |  |  | P | CON | **1.666** | **0.438** | **2.893** |
|  |  |  |  | ZEB | 0.720 | -0.738 | 2.179 |
|  |  | *Senecio*  *vulgaris* | K | CON | 0.673 | -1.483 | 2.829 |
|  |  |  |  | ZEB | 0.000 | -2.422 | 2.422 |
|  |  |  | P | CON | 0.622 | -2.285 | 3.529 |
|  |  |  |  | ZEB | -0.565 | -2.720 | 1.591 |
|  |  | *Sonchus*  *oleraceus* | K | CON | 0.000 | -2.526 | 2.526 |
|  |  |  |  | ZEB | 0.000 | -2.519 | 2.519 |
|  |  |  | P | CON | -0.385 | -2.332 | 1.561 |
|  |  |  |  | ZEB | 0.248 | -2.235 | 2.730 |
|  |  | *Tripleurospermum*  *inodorum* | K | CON | -0.363 | -2.066 | 1.340 |
|  |  |  |  | ZEB | 0.855 | -1.152 | 2.862 |
|  |  |  | P | CON | -0.499 | -1.424 | 0.425 |
|  |  |  |  | ZEB | -0.161 | -1.019 | 0.696 |

*(Table 9 continued from previous page)*

| **Plant family** | **Status** | **Species** | **Region** | **Treatment** | **predicted** | **ci.lb** | **ci.ub** |
| --- | --- | --- | --- | --- | --- | --- | --- |
| Plantaginaceae | non-native | *Veronica persica* | K | CON | 0.000 | -2.322 | 2.322 |
|  |  |  |  | ZEB | **1.538** | **0.013** | **3.063** |
|  |  |  | P | CON | 0.235 | -1.237 | 1.707 |
|  |  |  |  | ZEB | -0.149 | -1.412 | 1.115 |
|  | native | *Plantago major* | K | CON | 0.090 | -1.732 | 1.911 |
|  |  |  |  | ZEB | 0.095 | -1.936 | 2.125 |
|  |  |  | P | CON | -1.176 | -2.389 | 0.037 |
|  |  |  |  | ZEB | 0.015 | -1.124 | 1.153 |
| Solanaceae | non-native | *Datura*  *stramonium* | K | CON | **2.525** | **0.968** | **4.081** |
|  |  |  |  | ZEB | **1.653** | **0.255** | **3.050** |
|  |  |  | P | CON | -0.302 | -1.925 | 1.322 |
|  |  |  |  | ZEB | -0.442 | -1.555 | 0.671 |
|  | native | *Solanum*  *nigrum* | K | CON | 0.000 | -2.494 | 2.494 |
|  |  |  |  | ZEB | 0.487 | -1.616 | 2.590 |
|  |  |  | P | CON | 0.208 | -1.178 | 1.594 |
|  |  |  |  | ZEB | -0.333 | -1.587 | 0.922 |

**Table S10** Estimates and confidence intervals of aboveground biomass effect sizes summarized at the region-level, in Fig**.** S4B). Significant effect sizes at the *P* < 0.05 threshold are marked in bold. Single block effect sizes are highlighted in red. K – Konstanz transplant region; P – Potsdam transplant region; CON – control treatment; ZEB – zebularine treatment; predicted – summarized effect size; ci.lb – lower 95% confidence interval; ci.ub – upper 95% confidence interval. Species names of the natives are in black, and the ones of the non-natives are in red.

| **Plant family** | **Status** | **Species** | **Region** | **Treatment** | **predicted** | **ci.lb** | **ci.ub** |
| --- | --- | --- | --- | --- | --- | --- | --- |
| Amaranthaceae | non-native | *Amaranthus*  *retroflexus* | K | CON | -0.391 | -1.360 | 0.578 |
|  |  |  |  | ZEB | 0.130 | -0.834 | 1.094 |
|  |  |  | P | CON | NA | NA | NA |
|  |  |  |  | ZEB | -0.548 | -1.556 | 0.460 |
|  | native | *Chenopodium*  *album* | K | CON | -0.286 | -0.755 | 0.183 |
|  |  |  |  | ZEB | -0.171 | -0.680 | 0.339 |
|  |  |  | P | CON | -1.049 | -3.317 | 1.219 |
|  |  |  |  | ZEB | 0.144 | -1.342 | 1.630 |
| Asteraceae | non-native | *Erigeron*  *canadensis* | K | CON | -0.108 | -0.605 | 0.389 |
|  |  |  |  | ZEB | -0.079 | -0.857 | 0.700 |
|  |  |  | P | CON | NA | NA | NA |
|  |  |  |  | ZEB | NA | NA | NA |
|  |  | *Erigeron annuus* | K | CON | **-0.581** | **-1.050** | **-0.112** |
|  |  |  |  | ZEB | -0.325 | -0.836 | 0.185 |
|  |  |  | P | CON | -0.505 | -1.148 | 0.137 |
|  |  |  |  | ZEB | -0.027 | -0.785 | 0.731 |
|  | native | *Lactuca*  *serriola* | K | CON | -0.521 | -1.086 | 0.043 |
|  |  |  |  | ZEB | -0.050 | -0.666 | 0.567 |
|  |  |  | P | CON | 0.924 | -0.079 | 1.927 |
|  |  |  |  | ZEB | 0.677 | -0.047 | 1.401 |
|  |  | *Senecio*  *vulgaris* | K | CON | **0.630** | **0.141** | **1.119** |
|  |  |  |  | ZEB | 0.551 | -0.215 | 1.316 |
|  |  |  | P | CON | -0.204 | -1.193 | 0.786 |
|  |  |  |  | ZEB | 0.388 | -0.573 | 1.348 |
|  |  | *Sonchus*  *oleraceus* | K | CON | -0.308 | -0.953 | 0.338 |
|  |  |  |  | ZEB | -0.047 | -0.702 | 0.607 |
|  |  |  | P | CON | 0.487 | -0.264 | 1.238 |
|  |  |  |  | ZEB | 0.459 | -0.322 | 1.240 |
|  |  | *Tripleurospermum*  *inodorum* | K | CON | 0.220 | -0.230 | 0.670 |
|  |  |  |  | ZEB | 0.146 | -0.267 | 0.558 |
|  |  |  | P | CON | 0.291 | -0.298 | 0.880 |
|  |  |  |  | ZEB | -0.274 | -1.090 | 0.542 |

*(Table 10 continued from previous page)*

| **Plant family** | **Status** | **Species** | **Region** | **Treatment** | **predicted** | **ci.lb** | **ci.ub** |
| --- | --- | --- | --- | --- | --- | --- | --- |
| Plantaginaceae | non-native | *Veronica persica* | K | CON | -0.033 | -0.460 | 0.394 |
|  |  |  |  | ZEB | 0.433 | -0.069 | 0.935 |
|  |  |  | P | CON | 0.507 | -0.357 | 1.372 |
|  |  |  |  | ZEB | **-0.922** | **-1.789** | **-0.055** |
|  | native | *Plantago major* | K | CON | 0.390 | -0.050 | 0.830 |
|  |  |  |  | ZEB | -0.359 | -0.909 | 0.192 |
|  |  |  | P | CON | -0.496 | -1.094 | 0.102 |
|  |  |  |  | ZEB | 0.329 | -0.382 | 1.040 |
| Solanaceae | non-native | *Datura*  *stramonium* | K | CON | 0.644 | -0.285 | 1.573 |
|  |  |  |  | ZEB | 0.276 | -0.415 | 0.966 |
|  |  |  | P | CON | -0.033 | -0.582 | 0.516 |
|  |  |  |  | ZEB | -0.413 | -1.066 | 0.240 |
|  | native | *Solanum*  *nigrum* | K | CON | -0.348 | -0.810 | 0.114 |
|  |  |  |  | ZEB | **-0.819** | **-1.302** | **-0.336** |
|  |  |  | P | CON | **1.008** | **0.278** | **1.739** |
|  |  |  |  | ZEB | **0.876** | **0.177** | **1.574** |

**Table S11** Estimates and confidence intervals of flowering probability effect sizes summarized at the region-level, in Fig. S4C). Significant effect sizes at the *P* < 0.05 threshold are marked in bold. Single field effect sizes are highlighted in red. K – Konstanz transplant region; P – Potsdam transplant region; CON – control treatment; ZEB – zebularine treatment; predicted – summarized effect size; ci.lb – lower 95% confidence interval; ci.ub – upper 95% confidence interval. Species names of the natives are in black, and the ones of the non-natives are in red.

| **Plant family** | **Status** | **Species** | **Region** | **Treatment** | **predicted** | **ci.lb** | **ci.ub** |
| --- | --- | --- | --- | --- | --- | --- | --- |
| Amaranthaceae | non-native | *Amaranthus*  *retroflexus* | K | CON | -1.644 | -3.686 | 0.398 |
|  |  |  |  | ZEB | -0.733 | -2.915 | 1.449 |
|  |  |  | P | CON | -1.037 | -4.073 | 1.998 |
|  |  |  |  | ZEB | -0.642 | -2.722 | 1.438 |
|  | native | *Chenopodium*  *album* | K | CON | -1.318 | -3.437 | 0.801 |
|  |  |  |  | ZEB | -0.713 | -2.128 | 0.701 |
|  |  |  | P | CON | 0.705 | -2.916 | 4.327 |
|  |  |  |  | ZEB | -0.427 | -3.578 | 2.725 |
| Asteraceae | non-native | *Erigeron*  *canadensis* | K | CON | 0.457 | -0.418 | 1.333 |
|  |  |  |  | ZEB | **1.698** | **0.474** | **2.923** |
|  |  |  | P | CON | NA | NA | NA |
|  |  |  |  | ZEB | 0.775 | -3.139 | 4.689 |
|  |  | *Erigeron annuus* | K | CON | **-1.272** | **-2.163** | **-0.381** |
|  |  |  |  | ZEB | -0.635 | -1.841 | 0.572 |
|  |  |  | P | CON | -0.020 | -1.607 | 1.567 |
|  |  |  |  | ZEB | 0.131 | -1.647 | 1.909 |
|  | native | *Lactuca*  *serriola* | K | CON | 1.317 | -0.262 | 2.897 |
|  |  |  |  | ZEB | 0.154 | -1.335 | 1.643 |
|  |  |  | P | CON | 0.288 | -1.947 | 2.524 |
|  |  |  |  | ZEB | -0.576 | -3.781 | 2.630 |
|  |  | *Senecio*  *vulgaris* | K | CON | -0.370 | -2.718 | 1.977 |
|  |  |  |  | ZEB | -0.592 | -2.910 | 1.726 |
|  |  |  | P | CON | -0.573 | -3.968 | 2.822 |
|  |  |  |  | ZEB | 1.488 | -1.588 | 4.564 |
|  |  | *Sonchus*  *oleraceus* | K | CON | **1.289** | **0.006** | **2.572** |
|  |  |  |  | ZEB | **1.334** | **0.081** | **2.588** |
|  |  |  | P | CON | -1.139 | -2.916 | 0.639 |
|  |  |  |  | ZEB | -0.199 | -1.461 | 1.063 |
|  |  | *Tripleurospermum*  *inodorum* | K | CON | 0.552 | -0.516 | 1.620 |
|  |  |  |  | ZEB | -0.636 | -1.782 | 0.511 |
|  |  |  | P | CON | 0.413 | -1.576 | 2.402 |
|  |  |  |  | ZEB | 0.083 | -1.260 | 1.425 |

*(Table 11 continued from previous page)*

| **Plant family** | **Status** | **Species** | **Region** | | **Treatment** | **predicted** | **ci.lb** | | **ci.ub** | |  |
| --- | --- | --- | --- | --- | --- | --- | --- | --- | --- | --- | --- |
| Plantaginaceae | non-native | *Veronica persica* | K | CON | | -0.695 | | -2.850 | | 1.459 | |
|  |  |  |  | ZEB | | -0.737 | | -3.009 | | 1.534 | |
|  |  |  | P | CON | | -0.145 | | -2.383 | | 2.092 | |
|  |  |  |  | ZEB | | 0.681 | | -1.595 | | 2.957 | |
|  | native | *Plantago major* | K | CON | | 0.000 | | -2.309 | | 2.309 | |
|  |  |  |  | ZEB | | -0.471 | | -2.653 | | 1.711 | |
|  |  |  | P | CON | | 0.485 | | -1.329 | | 2.299 | |
|  |  |  |  | ZEB | | 0.392 | | -0.921 | | 1.706 | |
| Solanaceae | non-native | *Datura*  *stramonium* | K | CON | | 1.222 | | -0.287 | | 2.731 | |
|  |  |  |  | ZEB | | 1.061 | | -0.238 | | 2.359 | |
|  |  |  | P | CON | | 1.694 | | -0.226 | | 3.613 | |
|  |  |  |  | ZEB | | -0.864 | | -2.613 | | 0.886 | |
|  | native | *Solanum*  *nigrum* | K | CON | | 0.000 | | -2.494 | | 2.494 | |
|  |  |  |  | ZEB | | -0.365 | | -2.791 | | 2.062 | |
|  |  |  | P | CON | | 1.056 | | -1.221 | | 3.333 | |
|  |  |  |  | ZEB | | -0.693 | | -2.314 | | 0.928 | |

**Table S12** Estimates and confidence intervals of reproductive biomass effect sizes summarized at the region-level, in Fig. S4D). Significant effect sizes at the *P* < 0.05 threshold are marked in bold. Single block effect sizes are highlighted in red. K – Konstanz transplant region; P – Potsdam transplant region; CON – control treatment; ZEB – zebularine treatment; predicted – summarized effect size; ci.lb – lower 95% confidence interval; ci.ub – upper 95% confidence interval. Species names of the natives are in black, and the ones of the non-natives are in red.

| **Plant family** | **Status** | **Species** | **Region** | **Treatment** | **predicted** | **ci.lb** | **ci.ub** |
| --- | --- | --- | --- | --- | --- | --- | --- |
| Amaranthaceae | non-native | *Amaranthus*  *retroflexus* | K | CON | **-1.792** | **-2.408** | **-1.175** |
|  |  |  |  | ZEB | **-1.994** | **-2.844** | **-1.143** |
|  |  |  | P | CON | NA | NA | NA |
|  |  |  |  | ZEB | 0.577 | -1.976 | 3.131 |
|  | native | *Chenopodium*  *album* | K | CON | **-1.025** | **-1.627** | **-0.423** |
|  |  |  |  | ZEB | **-0.885** | **-1.689** | **-0.081** |
|  |  |  | P | CON | -0.377 | -2.544 | 1.789 |
|  |  |  |  | ZEB | 0.647 | -1.151 | 2.444 |
| Asteraceae | non-native | *Erigeron*  *canadensis* | K | CON | 0.242 | -0.606 | 1.090 |
|  |  |  |  | ZEB | 0.021 | -1.042 | 1.084 |
|  |  |  | P | CON | NA | NA | NA |
|  |  |  |  | ZEB | NA | NA | NA |
|  |  | *Erigeron annuus* | K | CON | 0.399 | -1.929 | 2.728 |
|  |  |  |  | ZEB | 0.057 | -0.814 | 0.928 |
|  |  |  | P | CON | **-1.695** | **-3.067** | **-0.323** |
|  |  |  |  | ZEB | -0.833 | -2.977 | 1.311 |
|  | native | *Lactuca*  *serriola* | K | CON | NA | NA | NA |
|  |  |  |  | ZEB | NA | NA | NA |
|  |  |  | P | CON | NA | NA | NA |
|  |  |  |  | ZEB | NA | NA | NA |
|  |  | *Senecio*  *vulgaris* | K | CON | 0.039 | -0.441 | 0.519 |
|  |  |  |  | ZEB | -0.037 | -0.858 | 0.785 |
|  |  |  | P | CON | NA | NA | NA |
|  |  |  |  | ZEB | 0.594 | -0.582 | 1.770 |
|  |  | *Sonchus*  *oleraceus* | K | CON | -0.185 | -1.365 | 0.995 |
|  |  |  |  | ZEB | -0.135 | -3.043 | 2.773 |
|  |  |  | P | CON | 0.363 | -0.657 | 1.383 |
|  |  |  |  | ZEB | 0.336 | -2.084 | 2.755 |
|  |  | *Tripleurospermum*  *inodorum* | K | CON | -0.471 | -0.944 | 0.003 |
|  |  |  |  | ZEB | -0.066 | -0.609 | 0.477 |
|  |  |  | P | CON | 0.278 | -0.327 | 0.882 |
|  |  |  |  | ZEB | -0.354 | -1.553 | 0.846 |

*(Table 12 continued from previous page)*

| **Plant family** | **Status** | **Species** | **Region** | **Treatment** | | **predicted** | **ci.lb** | **ci.ub** |  |
| --- | --- | --- | --- | --- | --- | --- | --- | --- | --- |
| Plantaginaceae | non-native | *Veronica persica* | K | | CON | **-0.895** | **-1.478** | **-0.311** | |
|  |  |  |  |  | ZEB | -0.316 | -0.862 | 0.229 | |
|  |  |  | P | | CON | 1.496 | -0.037 | 3.029 | |
|  |  |  |  |  | ZEB | -0.366 | -1.858 | 1.127 | |
|  | native | *Plantago major* | K | | CON | **0.472** | **0.032** | **0.913** | |
|  |  |  |  |  | ZEB | -0.236 | -0.698 | 0.225 | |
|  |  |  | P | | CON | -0.938 | -1.908 | 0.033 | |
|  |  |  |  |  | ZEB | 0.361 | -0.371 | 1.092 | |
| Solanaceae | non-native | *Datura*  *stramonium* | K | | CON | -0.019 | -2.424 | 2.385 | |
|  |  |  |  |  | ZEB | 0.995 | -0.746 | 2.736 | |
|  |  |  | P | | CON | -0.109 | -0.734 | 0.516 | |
|  |  |  |  |  | ZEB | -0.075 | -1.041 | 0.891 | |
|  | native | *Solanum*  *nigrum* | K | | CON | -0.293 | -0.754 | 0.167 | |
|  |  |  |  |  | ZEB | **-0.638** | **-1.116** | **-0.160** | |
|  |  |  | P | | CON | **1.053** | **0.385** | **1.722** | |
|  |  |  |  |  | ZEB | **0.668** | **0.094** | **1.241** | |

**Table S13** Estimates and confidence intervals of across-region summarized effect sizes for survival (Fig. 1A). Significant effect sizes at the *P* < 0.05 threshold are marked in bold. CON – control treatment; ZEB – zebularine treatment; predicted – summarized effect size; ci.lb – lower 95% confidence interval; ci.ub – upper 95% confidence interval. Species names of the natives are in black, and the ones of the non-natives are in red.

| **Plant family** | **Status** | **Species** | **Treatment** | **predicted** | **ci.lb** | **ci.ub** |
| --- | --- | --- | --- | --- | --- | --- |
| Amaranthaceae | non-native | *Amaranthus*  *retroflexus* | CON | -0.310 | -2.085 | 1.464 |
|  |  |  | ZEB | -0.695 | -2.105 | 0.716 |
|  | native | *Chenopodium album* | CON | -0.898 | -2.276 | 0.480 |
|  |  |  | ZEB | 0.469 | -0.823 | 1.761 |
| Asteraceae | non-native | *Erigeron canadensis* | CON | -0.895 | -2.167 | 0.378 |
|  |  |  | ZEB | 0.666 | -0.366 | 1.699 |
|  |  | *Erigeron annuus* | CON | -0.333 | -1.559 | 0.893 |
|  |  |  | ZEB | 0.374 | -0.768 | 1.516 |
|  | native | *Lactuca serriola* | CON | 0.569 | -1.585 | 2.723 |
|  |  |  | ZEB | 0.111 | -0.594 | 0.815 |
|  |  | *Senecio vulgaris* | CON | 0.655 | -1.077 | 2.387 |
|  |  |  | ZEB | -0.315 | -1.925 | 1.295 |
|  |  | *Sonchus oleraceus* | CON | -0.242 | -1.784 | 1.300 |
|  |  |  | ZEB | 0.069 | -1.539 | 1.677 |
|  |  | *Tripleurospermum inodorum* | CON | -0.468 | -1.281 | 0.344 |
|  |  |  | ZEB | -0.004 | -0.793 | 0.784 |
| Plantaginaceae | non-native | *Veronica persica* | CON | 0.153 | -0.951 | 1.257 |
|  |  |  | ZEB | 0.638 | -1.011 | 2.287 |
|  | native | *Plantago major* | CON | -0.733 | -1.916 | 0.450 |
|  |  |  | ZEB | 0.034 | -0.960 | 1.027 |
| Solanaceae | non-native | *Datura stramonium* | CON | 1.112 | -1.677 | 3.901 |
|  |  |  | ZEB | 0.561 | -1.490 | 2.612 |
|  | native | *Solanum nigrum* | CON | 0.159 | -1.053 | 1.371 |
|  |  |  | ZEB | -0.118 | -1.195 | 0.960 |

**Table S14** Estimates and confidence intervals of across-region summarized effect sizes for aboveground biomass (Fig. 1B). Significant effect sizes at the *P* < 0.05 threshold are marked in bold. CON – control treatment; ZEB – zebularine treatment; predicted – summarized effect size; ci.lb – lower 95% confidence interval; ci.ub – upper 95% confidence interval. Species names of the natives are in black, and the ones of the non-natives are in red.

| **Plant family** | **Status** | **Species** | **Treatment** | **predicted** | **ci.lb** | **ci.ub** |
| --- | --- | --- | --- | --- | --- | --- |
| Amaranthaceae | non-native | *Amaranthus*  *retroflexus* | CON | NA | NA | NA |
|  |  |  | ZEB | -0.068 | -0.762 | 0.627 |
|  | native | *Chenopodium album* | CON | -0.338 | -0.791 | 0.114 |
|  |  |  | ZEB | -0.136 | -0.590 | 0.317 |
| Asteraceae | non-native | *Erigeron canadensis* | CON | NA | NA | NA |
|  |  |  | ZEB | NA | NA | NA |
|  |  | *Erigeron annuus* | CON | **-0.552** | **-0.923** | **-0.182** |
|  |  |  | ZEB | -0.251 | -0.630 | 0.128 |
|  | native | *Lactuca serriola* | CON | 0.139 | -1.271 | 1.550 |
|  |  |  | ZEB | 0.288 | -0.422 | 0.999 |
|  |  | *Senecio vulgaris* | CON | 0.328 | -0.458 | 1.113 |
|  |  |  | ZEB | 0.485 | -0.051 | 1.020 |
|  |  | *Sonchus oleraceus* | CON | 0.066 | -0.712 | 0.843 |
|  |  |  | ZEB | 0.161 | -0.340 | 0.663 |
|  |  | *Tripleurospermum inodorum* | CON | 0.244 | -0.093 | 0.580 |
|  |  |  | ZEB | -0.009 | -0.423 | 0.405 |
| Plantaginaceae | non-native | *Veronica persica* | CON | 0.117 | -0.355 | 0.588 |
|  |  |  | ZEB | -0.193 | -1.521 | 1.135 |
|  | native | *Plantago major* | CON | 0.071 | -0.297 | 0.440 |
|  |  |  | ZEB | -0.058 | -0.702 | 0.587 |
| Solanaceae | non-native | *Datura stramonium* | CON | 0.198 | -0.431 | 0.827 |
|  |  |  | ZEB | -0.082 | -0.752 | 0.589 |
|  | native | *Solanum nigrum* | CON | 0.327 | -1.013 | 1.667 |
|  |  |  | ZEB | 0.011 | -1.621 | 1.642 |

**Table S15** Estimates and confidence intervals of across-region summarized effect sizes for flowering probability (Fig. 1C). Significant effect sizes at the *P* < 0.05 threshold are marked in bold. CON – control treatment; ZEB – zebularine treatment; predicted – summarized effect size; ci.lb – lower 95% confidence interval; ci.ub – upper 95% confidence interval. Species names of the natives are in black, and the ones of the non-natives are in red.

| **Plant family** | **Status** | **Species** | **Treatment** | **predicted** | **ci.lb** | **ci.ub** |
| --- | --- | --- | --- | --- | --- | --- |
| Amaranthaceae | non-native | *Amaranthus*  *retroflexus* | CON | -1.455 | -3.149 | 0.239 |
|  |  |  | ZEB | -0.685 | -2.191 | 0.820 |
|  | native | *Chenopodium album* | CON | -0.802 | -2.631 | 1.027 |
|  |  |  | ZEB | -0.665 | -1.956 | 0.625 |
| Asteraceae | non-native | *Erigeron canadensis* | CON | NA | NA | NA |
|  |  |  | ZEB | **1.614** | **0.537** | **2.691** |
|  |  | *Erigeron annuus* | CON | -0.802 | -2.002 | 0.398 |
|  |  |  | ZEB | -0.463 | -1.365 | 0.440 |
|  | native | *Lactuca serriola* | CON | 0.975 | -0.315 | 2.264 |
|  |  |  | ZEB | -0.048 | -1.318 | 1.221 |
|  |  | *Senecio vulgaris* | CON | -0.436 | -2.366 | 1.495 |
|  |  |  | ZEB | 0.192 | -1.784 | 2.168 |
|  |  | *Sonchus oleraceus* | CON | 0.156 | -2.217 | 2.530 |
|  |  |  | ZEB | 0.570 | -0.933 | 2.072 |
|  |  | *Tripleurospermum inodorum* | CON | 0.521 | -0.420 | 1.462 |
|  |  |  | ZEB | -0.333 | -1.205 | 0.539 |
| Plantaginaceae | non-native | *Veronica persica* | CON | -0.431 | -1.983 | 1.121 |
|  |  |  | ZEB | -0.030 | -1.637 | 1.578 |
|  | native | *Plantago major* | CON | 0.300 | -1.126 | 1.726 |
|  |  |  | ZEB | 0.163 | -0.963 | 1.288 |
| Solanaceae | non-native | *Datura stramonium* | CON | **1.401** | **0.218** | **2.584** |
|  |  |  | ZEB | 0.192 | -1.685 | 2.068 |
|  | native | *Solanum nigrum* | CON | 0.576 | -1.106 | 2.257 |
|  |  |  | ZEB | -0.592 | -1.940 | 0.756 |

**Table S16** Estimates and confidence intervals of across-region summarized effect sizes for reproductive biomass (Fig. 1D). Significant effect sizes at the *P* < 0.05 threshold are marked in bold. CON – control treatment; ZEB – zebularine treatment; predicted – summarized effect size; ci.lb – lower 95% confidence interval; ci.ub – upper 95% confidence interval. Species names of the natives are in black, and the ones of the non-natives are in red.

| **Plant family** | **Status** | **Species** | **Treatment** | **predicted** | **ci.lb** | **ci.ub** |
| --- | --- | --- | --- | --- | --- | --- |
| Amaranthaceae | non-native | *Amaranthus*  *retroflexus* | CON | NA | NA | NA |
|  |  |  | ZEB | -1.051 | -3.039 | 0.936 |
|  | native | *Chenopodium album* | CON | **-0.957** | **-1.505** | **-0.409** |
|  |  |  | ZEB | -0.259 | -1.682 | 1.163 |
| Asteraceae | non-native | *Erigeron canadensis* | CON | NA | NA | NA |
|  |  |  | ZEB | NA | NA | NA |
|  |  | *Erigeron annuus* | CON | -0.345 | -2.284 | 1.594 |
|  |  |  | ZEB | -0.230 | -1.002 | 0.542 |
|  | native | *Lactuca serriola* | CON | NA | NA | NA |
|  |  |  | ZEB | NA | NA | NA |
|  |  | *Senecio vulgaris* | CON | NA | NA | NA |
|  |  |  | ZEB | 0.159 | -0.513 | 0.830 |
|  |  | *Sonchus oleraceus* | CON | 0.129 | -0.643 | 0.900 |
|  |  |  | ZEB | 0.111 | -1.125 | 1.348 |
|  |  | *Tripleurospermum inodorum* | CON | -0.121 | -0.853 | 0.611 |
|  |  |  | ZEB | -0.092 | -0.541 | 0.358 |
| Plantaginaceae | non-native | *Veronica persica* | CON | 0.202 | -2.101 | 2.504 |
|  |  |  | ZEB | -0.329 | -0.829 | 0.170 |
|  | native | *Plantago major* | CON | -0.247 | -1.714 | 1.221 |
|  |  |  | ZEB | -0.008 | -0.577 | 0.560 |
| Solanaceae | non-native | *Datura stramonium* | CON | -0.098 | -0.685 | 0.489 |
|  |  |  | ZEB | 0.233 | -0.679 | 1.145 |
|  | native | *Solanum nigrum* | CON | 0.363 | -0.944 | 1.670 |
|  |  |  | ZEB | 0.005 | -1.274 | 1.284 |

**Table S17** Soil parameters at field sites. For each transplant field site, the average of 11-12 soil samples is given (collected in four species plots per block). Samples were collected in July/August 2016. BoGa – Botanical Garden of Konstanz. Hättli - Konstanz/Hättelihof. Uni – University of Konstanz. DB - Potsdam/Botanical Garden. GR - Ludwigsfelde/Gröben (close to Potsdam). MQ - Potsdam-Marquardt. WeightRatio – dry-to-fresh mass ratio. Npercent – relative [%] nitrogen content of dry mass. Cpercent – relative [%] carbon content of dry mass. CNratio – carbon-to-nitrogen ratio of dry mass. pHcurrent – pH value in ddH_2_O. pHpotential – pH value in CaCl_2_. Phosphate – total phosphate content [g kg^-1^ dry mass]. Nitrogen – total nitrogen content [g kg^-1^ dry mass]. Ammonium – plant-available ammonium [mg kg^-1^ dry mass]. PhosphatePA – plant-available phosphate content [mg L^-1^ solved dry mass]. OrganicMatter – relative [%] organic matter in dry mass after loss-on-ignition. (See Methods S4 for a detailed description of sampling and soil analysis.)

| **Region** | **Konstanz** | | | **Potsdam** | | | |
| --- | --- | --- | --- | --- | --- | --- | --- |
| **Field site** | **BoGa** | **Hättli** | **Uni** | **DB** | **GR** | **MQ** | |
| **WeightRatio** | 0.75 | 0.81 | 0.77 | 0.90 | 0.97 | 0.88 |  |
| **Npercent**  **[% dry mass]** | 0.26 | 0.21 | 0.20 | 0.14 | 0.09 | 0.13 |  |
| **Cpercent**  **[% dry mass]** | 5.95 | 3.51 | 5.49 | 1.58 | 1.07 | 1.96 |  |
| **CNratio** | 23.84 | 20.27 | 29.33 | 11.60 | 11.58 | 14.99 |  |
| **pHcurrent** | 7.99 | 7.81 | 8.05 | 5.91 | 5.62 | 7.03 |  |
| **pHpotential** | 7.34 | 7.24 | 7.35 | 5.42 | 5.01 | 6.49 |  |
| **Phosphate**  **[g kg^-1^ dry mass]** | 1.18 | 0.67 | 0.64 | 0.53 | 0.41 | 1.18 |  |
| **Nitrogen**  **[g kg^-1^ dry mass]** | 2.65 | 1.97 | 2.02 | 1.66 | 0.97 | 1.55 |  |
| **Ammonium**  **[mg kg^-1^ dry mass]** | 3.35 | 1.60 | 1.20 | 2.83 | 2.02 | 2.73 |  |

*(Table S17 continued from previous page)*

| **Region** | **Konstanz** | | | **Potsdam** | | |
| --- | --- | --- | --- | --- | --- | --- |
| **Field site** | **BoGa** | **Hättli** | **Uni** | **DB** | **GR** | **MQ** |
| **PhosphatePA**  **[mg L^-1^ solved dry mass]** | 2.41 | 0.59 | 0.70 | 2.87 | 0.13 | 9.03 |
| **OrganicMatter**  **[% dry mass]** | 6.45 | 5.73 | 7.86 | 4.19 | 3.02 | 4.55 |
| **Nr. of samples** | 12 | 11 | 12 | 12 | 12 | 12 |

**Table S18** Climatic differences Data of Konstanz and Potsdam weather stations averaged over years 1975 to 2015. (Based on data available from the German weather service, Deutscher Wetterdienst DWD, 2019. See below for data sources.)

| **Climate variable** | **Konstanz**  **weather station** | **Potsdam**  **weather station** |
| --- | --- | --- |
| **latitude^a^** | 47.67° N | 52.38° N |
| **longitude^a^** | 9.18° E | 13.05° E |
| **maximum day length** | 16.0^d^ | 16.8^e^ |
| **minimum temperature [°C]^c^** | -10.06 | -14.00 |
| **maximum temperature [°C]^c^** | 33.3 | 34.53 |
| **average air temperature [°C]^b^** | 9.82 | 9.30 |
| **sun hours per year^b^** | 1701.47 | 1740.02 |
| **annual precipitation [mm]^b^** | 850.86 | 582.79 |
| **nr of frost days**  **(< 0°C) per year^c^** | 69.90 | 83.00 |
| **nr. of hot (≥ 30 °C) days**  **per year^c^** | 10.63 | 10.78 |

Data sources:

^a^ https://www.dwd.de/DE/leistungen/klimadatendeutschland/stationsuebersicht.html

(accessed 17 March 2019).

^b^ Values based on monthly data by the German weather service (Deutscher Wetterdienst, DWD) https://www.dwd.de/DE/leistungen/klimadatendeutschland/klarchivtagmonat.html. (accessed 18 March 2019)

^c^ Values based on daily data by the German weather service (Deutscher Wetterdienst, DWD) https://www.dwd.de/DE/leistungen/klimadatendeutschland/klarchivtagmonat.html. (accessed 18 March 2019)

^d^ https://sunrisesunset.de/sonne/deutschland/konstanz/ (accessed 18 March 2019).

^e^ https://sunrisesunset.de/sonne/deutschland/potsdam/ (accessed 18 March 2019).

**Table S19** First records of the non-native study species in Germany and in the states of Germany in which Konstanz (Baden-Württemberg) and Potsdam (Brandenburg, but close to Berlin) are.

| Species | Plant family | Native range | First record in Germany^a^ | First record in Baden-  Württemberg | First record in Berlin (BE), Brandenburg (BR) or Berlin-Brandenburg (BB) |
| --- | --- | --- | --- | --- | --- |
| *Amaranthus retroflexus* | Amaranthaceae | Tropical Americas  (Costea, Weaver, & Tardif, 2011) | 1815 | 1820; maybe even earlier (1813)  (Sebald, Philippi, & Seybold, 1993) | 1859 (BB)  (Ascherson, 1859)^i^ |
| *Erigeron annuus* | Asteraceae | North America  (Frey, 2003) | 18^th^ century | 1562?, but definitely in the 18^th^ century  (Sebald, Phillippi, Seybold, & Wörz, 1996) | 1859 (BE)  (Ascherson, 1859) |
| *Erigeron canadensis* |  | North America  (Weaver, 2011)^e^ | 1700 | 1728 (Ulm)  (Sebald, Phillippi, et al., 1996) | 1787 (BE) and 1859 (BR)  (Ascherson, 1859; Willdenow, 1787) |
| *Veronica persica* | Plantaginaceae | Caucasus region  (Fischer, 1987) | 1805 | 1805 (Karlsruhe)  (Sebald, Seybold, Philippi, Kleinsteuber, & Lange, 1996) | 1850 (BR) and 1859 (BE)  (Ascherson, 1859) |
| *Datura stramonium* | Solanaceae | most likely the Americas  (Weaver & Warwick, 1984) | 1584 | 1728 (Ulm)  (Sebald, Seybold, et al., 1996) | 1787 (BE) and 1859 (BR)  (Ascherson, 1859; Willdenow, 1787) |

^a^Based on the Biolflor database (Kühn, Durka, & Klotz, 2004).

**Methods S1** Single-species analyses of fitness parameters

We tested for each species separately the effects of region of the field sites, zebularine treatment, origin and their interactions. In all models, we included as fixed terms the factors region of the field sites (Konstanz vs. Potsdam), zebularine treatment (untreated vs. treated), origin of maternal lines (Konstanz region vs. Potsdam region) and their two- and three-way interactions. As random terms, we included field site and block nested within field to account for non-independence of plants in the same block and field site, and maternal lines to account for non-independence of offspring from the same mother plant.

We analyzed aboveground biomass and reproductive biomass with linear mixed models, implemented in the R package ‘lme4’ (Bates, Mächler, Bolker, & Walker, 2015). Both biomass variables were power transformed (see Tables S5 and S7) to fulfil the assumptions of normality of the residuals and to reduce heterogeneity of variance. We analyzed survival probability (0, 1) and flowering probability (0, 1) with generalized linear mixed models with a binomial distribution with the MCMCGLMM package (Hadfield, 2010), since generalized mixed models with the R package ‘lme4’ (Bates et al., 2015) often did not converge. There was nearly complete survival and flowering in certain field sites (a phenomenon known as quasi-complete separation; see e.g. Albert & Anderson, 1984; Rainey, 2016; Sauter & Held, 2016). The reason for this is that, in such cases, the likelihood function often has no maximum. Therefore, instead of a probabilistic approach, we used a Markov chain Monte Carlo (MCMC) estimation approach implemented in the MCMCGLMM package (Hadfield, 2010) following a worked example provided at https://ms.mcmaster.ca/~bolker/R/misc/foxchapter/bolker_chap.html (last accessed on 28 March 2019). Priors for random effects, observation-level variance and the fixed effects were specified as recommended in the worked example. We used the logit link (family = categorical), a burn-in period of at least 100,000 iterations, and thinned at least every 900 iterations until we had an effective sample size of ~10,000 draws. We checked that draws were not affected by autocorrelation (non-independence between successive samples in the chain) and that the model had converged (no trend in the time series, i.e. traces of the chains are stationary). The effect of each fixed effect was estimated using the calculated 95% credible interval of their posterior distribution and the computed probability that such an effect is larger than zero (PMCMC).

**Methods S2** Effect size calculation in meta-regression

The number of local and non-local plants represented in each effect size differed between block-level effect sizes (1-7 plant individuals) and field-level effect sizes (1-21). Unfortunately, in some species, there were considerable amounts of missing data for all field sites of a transplant region, due to either very low survival (e.g. in the non-native *Erigeron canadensis* in Potsdam) or very few flowering plants (e.g. in the native *Lactuca serriola* in both Konstanz and Potsdam) (see Figs. S5-S16). But to calculate an effect size, we needed data for at least one plant individual in each group (local vs. non-local) for the log odds ratio, and at least two individuals in each group (local vs. non-local) for the standardized mean difference (SMD). Therefore, for some species, we sometimes could not calculate effect sizes and summarize them at the region-level (see Fig. S4), or, when effect sizes for one of the two regions were missing entirely, we could not summarize them across regions (see Fig. 1). Missing effect sizes are indicated with NA in figures.

**Methods S3** Effect sizes corrected with the ratio of sample sizes of the compared groups

For the analyses of the binomial variables (survival, flowering probability), a zero in one of the cells of a 2×2 contingency table can be a problem for effect size calculation (e.g. the odds ratio). Therefore, routinely a so-called continuity correction is added to every cell of a contingency table that has at least one zero. The most commonly used continuity correction value is 0.5 (Sweeting, Sutton, & Lambert, 2004; Viechtbauer, 2010), and the ‘escalc’ function in the metafor package (Viechtbauer, 2010) by default adds this value to tables with zeroes. However, when sample sizes between the local and non-local groups become increasingly unbalanced, a continuity correction of 0.5 can lead to erroneous effect sizes, if events are rare (e.g. in less than 1% of all cases; Sweeting et al*.*, 2004).

Our data showed both high rates of survival and flowering (i.e. rare events of dead or non-flowering plants), and occasionally huge sample size differences (with a maximum ratio of 1:16 between local and non-local plants). Therefore, we calculated a second set of effect sizes, corrected with the ratio of sample sizes of the compared groups, as proposed by Sweeting et al*.* (2004). Based on the ratio R of sample sizes between both groups, with R = n_non-local_/n_local_, a continuity correction (non-local_CC_ or local_CC_) is added to the cells of the respective group. The non-local_CC_ is calculated as non-local_CC_ = R/(1+R), and the local_CC_ for the treatment group as local_CC_ = 1/(1+R). (It follows that the sum of both non-local_CC_ and local_CC_ is 1.) In cases with balanced sample sizes (i.e. at a R of 1:1), both the standard continuity correction in the metafor package (Viechtbauer, 2010) and the method by Sweeting et al*.* (2004) give the same results. Meta-regressions with both methods gave very similar results (see Table 3).

Finally, the method by Sweeting et al*.* (2004) allows to include tables with two zeroes in events or non-events (i.e. complete flowering or complete non-flowering) in the analysis with metafor (Viechtbauer, 2010), whereas the standard method does not. Effect sizes of zero do not contribute significantly to the overall result when odds ratios are used (Friedrich, Adhikari, & Beyene, 2007), but it helped to better visualize effect sizes of zero in forest plots (Figs. 1 and S4).

**Methods S4** Soil parameter measurements in field sites

To assess the soil parameters in the six field sites, we randomly collected 11-12 soil samples (each at least 300 cm^3^) per field site at the end of July 2016 down to a depth of 15 – 22 cm. The 72 soil samples were stored in plastic bags at 8°C. For each sample, we determined dry-to-fresh mass ratio, organic matter after loss-on-ignition, plant-available phosphate according to Olsen et al*.* (1954), plant-available ammonium, total phosphate content, total nitrogen content, relative carbon content, relative nitrogen content, pH-value and C:N ratio. The averaged ratio of dry-to-fresh mass was determined by weighing two replicate subsamples of 15 – 82 g per soil sample and drying it for 48 h at 105°C in a drying chamber (M 115, Binder GmbH, Tuttlingen, Germany). The percentage of organic matter was obtained as the mean percentage weight loss of two subsamples (subsample weight: 170 – 490 mg), incinerated for 2h at 550°C in a muffle furnace (Carbolite LHT, Carbolite Gero Limited, Hope Valley, UK).

Plant-available phosphate (mg P L^-1^ dry mass) was extracted using three subsamples (2.5 g of fresh weight), following Olsen et al*.* (1954), and concentration was determined photometrically at 880 nm (DR/2000, Hach Lange GmbH, Düsseldorf, Germany) and results averaged over three replicated measurements per subsample. Plant-available ammonium (mg NH_4_ kg^-1^ dry mass) was determined using three subsamples (5 g of fresh weight) diluted each in 50 ml of 2M KCl (99.5 % p.a., Roth, Karlsruhe, Germany), shaken for 30 min (Level 2/9, REAX 2, Heidolph, Schwabach, Germany) and filtered with a glass fibre filter (MG160, ⌀ 150mm, Qty 50, glass-micro fibre discs, Sartorius, Göttingen, Germany). Concentration was determined photometrically at 425 nm (DR/2000, Hach Lange GmbH, Düsseldorf, Germany) adding one drop of mineral stabilizer solution (Hach Lange GmbH, Karlsruhe, Germany), one drop of polyvinyl alcohol dispersing agent (Hach Lange GmbH, Düsseldorf, Germany) and 0.2 ml of Nessler’s reagent (Hach Lange GmbH, Düsseldorf, Germany). The measurement was replicated three times for each of the three subsamples and the results were averaged per soil sample. The amounts of total nitrogen and total phosphate were obtained according to Kneis et al*.* (2006) and Heinze et al. (2016), using 250 mg of dried soil and chemical digestion for 15 min at 450°C (Digesdahl digestion apparatus, Hach Lange GmbH, Düsseldorf, Germany), and applying a mixture of 4 mL of H_2_SO_4_^+^ first (95 %, AnalaR NORMAPUR, VWR, Radnor/Pennsylvania, USA) for 8 min and 15 ml of H_2_O_2_ second (30 %, AnalaR NORMAPUR, VWR, Radnor/Pennsylvannia, USA) for 10 min. Samples were heated at 100°C in a water quench (using pumice stones) for 15 min, brought to pH 2 – 2.5 with 67.5% KOH (VWR, Radnor/Pennsylvania, USA), and filled with double-distilled H_2_O up to 100 mL. Concentrations were then determined photometrically at 425 nm for total nitrogen and 880 nm for total phosphate (DR/2000, HACH, Düsseldorf, Germany). Both total nitrogen (g N kg^-1^ dry mass) and total phosphate (g P kg^-1^ dry mass) were extracted using two subsamples and measurements were replicated three times per subsample. Actual pH was measured on subsamples of 5 g fresh mass at a soil:water ratio of 1:5 (WTW 325 pH meter, Germany) using double-distilled H_2_O after 30 min of dilution (Heinze et al., 2016). Subsamples were in the same ratio diluted in 0.01M CaCl_2_^-^ to measure potential pH. To measure C:N ratio, subsamples of 40 – 70 mg were sieved to a grain size of 1 mm and ground in a mixer mill (25 Hertz, 3 min, MM 200, Retsch, Haan, Germany) for 3 min. Samples were then transferred to aluminium cartridges (HE 25208000, 10 x 10, HEKAtech GmbH, Wegberg, Germany) and measured with a C:N analyzer (EA3000 CHNS-O, Eurovector Srl, Pavia, Italia).

**Notes S1** Results of the single species analyses.

**Survival**

Of the 3729 plants included in our survival analyses, 2993 (80.3%) survived until harvest, and survival ranged from 42.9% for the native *Lactuca serriola* to 97.9% for the native *Senecio vulgaris*. The single-species analyses showed that survival probabilities were for all 12 study species similar in the Konstanz and Potsdam transplant regions and not affected by the zebularine treatment (Table S5). For the non-native *Datura stramonium*, plants from the Konstanz origin had a significantly higher survival than those from the Potsdam origin (significant origin effect in in Table S5, Fig. S15), but this effect was only visible in the Konstanz transplant region (significant region:origin interaction Table S5, Fig. S15). In the Potsdam transplant region, on the other hand, the local plants of the native *L. serriola* had a higher survival probability than the non-local ones, whereas in the Konstanz transplant region this difference was absent (significant region:origin interaction in Table S5, Fig. S9).

## Aboveground biomass

For the subset of plants that survived until harvest, the single-species analyses showed that three of the 12 species, i.e. the non-native *Amaranthus retroflexus* and *D. stramonium*, and the native *Chenopodium album*, produced significantly more aboveground biomass in the Potsdam transplant region than in the Konstanz transplant region, whereas the reverse was true for the native *Plantago major* (Table S6, Figs. S5-S6 and S14-S15). The origin effects also varied among species: for the non-native *D. stramonium* and *Veronica persica*, and the native *S. vulgaris*, plants from the Konstanz origin produced more biomass than those from the Potsdam origin (Table S6, Figs. S10, S13 and S15), whereas the reverse was true for the non-native *Erigeron annuus*, and the native *L. serriola* and *Solanum nigrum* (Table S6, Figs. S8, S9 and S16). There were significant region:origin interactions for aboveground biomass in five of the 12 species (Table S6). However, only for one of those five species, the native *S. vulgaris*, the local plants produced more biomass than the non-local plants, both in the Konstanz and Potsdam transplant regions (Fig. S10). For the other four species, the non-native *A. retroflexus*, *D. stramonium* and *E. annuus*, and the native *L. serriola*, the non-local plants tended to produce more aboveground biomass than the local plants, at least in one of the two transplant regions (Figs. S6, S8, S9, and S15). So, the single-species analyses of aboveground biomass production provided more evidence for local maladaptation than adaptation. The zebularine treatment had negative effects on biomass production of seven of the species (see Table S6 and Figs. S8-S14) and a positive effect for one of them (see Table S6 and Fig. S6), but it did not significantly affect the expression of local adaptation or maladaptation (no significant region:treatment:origin interaction in Table S6).

**Flowering probability**

Of the 2953 plants included in our flowering analyses, 2316 (78.4%) flowered until harvest. All 12 species flowered, but the flowering percentage ranged from 11.1% for the native *L. serriola* to 97.4% for the native *S. nigrum* (Table S7). The single-species analyses showed that for all 12 study species flowering probabilities were similar in the Konstanz and Potsdam transplant regions (Table S7). For two of the non-native species, *A. retroflexus* and *E. annuus*, plants from the Potsdam origin were more likely to flower than those from the Konstanz origin (Table S7, Figs. S5 and S8). For *A. retroflexus* the origin effect was most pronounced in the Konstanz transplant region, whereas in the Potsdam transplant region plants from Konstanz tended to be more likely to flower than the ones from Potsdam (significant region:origin interaction in Table S7, Fig. S5). On the other hand, for the non-native *D. stramonium* local plants were more likely to flower than the non-local plants (significant region:origin interaction in Table S7, Fig. S15). The zebularine treatment had a significantly negative effect on flowering of the native *Sonchus oleraceus* (Table S7, Fig. 11). Interestingly, the flowering advantage of local over non-local plants in *D. stramonium* tended to disappear in the zebularine treatment, as indicated by a marginally non-significant (*P* = 0.052) region:origin:zebularine interaction (Table S7, Fig. S15). So, overall, the single-species analyses of flowering probability provided some scant evidence for both local maladaptation and local adaptation, and showed that the latter might be mediated by DNA methylation.

**Reproductive biomass**

For the subset of plants that flowered, the single-species analyses showed that six of the 12 species, i.e. the non-native *A. retroflexus*, *D. stramonium*, *E. canadensis* and *V. persica*, and the native *Ch. album* and *L. serriola*, produced significantly more reproductive biomass in the Potsdam transplant region than in the Konstanz transplant region, whereas the reverse was true for the native *P. major* (Table S8, Figs. S5-S7, S9, S13-S15). The origin effects also varied among species: for the native *Ch. album*, plants from the Konstanz origin tended to produce more reproductive biomass than those from the Potsdam origin (Table S8, Fig. S6), whereas the reverse was true for the non-native *A. retroflexus* and *V. persica* and the native *S. nigrum* (Table S8, Figs. S5, S13 and S16). There were significant region:origin interactions for reproductive biomass in five of the 12 species (Table S8). However, only for one of those five species, the native *S. vulgaris*, the local plants produced more reproductive biomass than the non-local plants, both in the Konstanz and Potsdam transplant regions (Fig. S10). For three other species, the non-native *A. retroflexus* and *E. annuus* and the native *Ch. album*, the non-local plants tended to produce more reproductive biomass than the local plants, at least in one of the two transplant regions (Figs. S5-S6, S8). For a fifth species, the non-native *E. canadensis*, local plants produced more reproductive biomass than non-local plants in Konstanz transplant region, but data is missing for local plants at Potsdam transplant region (Fig. S8). So, the single-species analyses of reproductive biomass provided partial evidence for local adaptation but particularly for local maladaptation. The zebularine treatment had negative effects on biomass production of seven of the 12 species (Table S8, Figs. S7, S10-S14 and S16) and a positive effect for one of them (Table S8, Fig. S6), but it did not significantly affect the expression of local adaptation or maladaptation (no significant region:treatment:origin interaction in Table S8).

**Notes S2** Results of the effect sizes summarized at the region-level.

## Survival

There were significant positive effect sizes for the native species *Lactuca serriola* in the control treatment but not in the zebularine treatment in the Potsdam field sites (Fig. S4A). For the non-native species *Veronica persica* and *Datura stramonium*, there were significant positive effect sizes in both the control and the zebularine treatments in the Konstanz field sites (Fig. S4A).

**Aboveground biomass**

Besides the negative effect size for *Erigeron annuus*, which was only apparent in the Konstanz transplant region despite its prominence in across-region effect sizes (Fig. 1), there were also a negative effect sizes for the non-native *V. persica* in the zebularine treatment of the Potsdam transplant region and for the native *Solanum nigrum* in the zebularine treatment of the Konstanz transplant region (Fig. S4B). Moreover, there were positive biomass effect sizes for the native *Senecio vulgaris* in the control treatment of the Konstanz transplant region and for the native *S. nigrum* in both treatments of the Potsdam transplant region (Fig. S4B).

**Flowering probability**

When the flowering effect sizes were plotted for each transplant region separately (Fig. S4C), it appears that at least in the Konstanz transplant region the positive effect size for *Erigeron canadensis* that was found for across-region effect sizes (Fig. 1) was not present in the control treatment. These plots also revealed a significant negative flowering effect size for the non-native *E. annuus* in the control treatment of the Konstanz transplant region, and significantly positive flowering effect sizes for the native *Sonchus oleraceus* in both treatments of the Konstanz transplant region (Fig. S4C).

**Reproductive biomass**

When the reproductive biomass effect sizes were plotted for each transplant region separately (Fig. S4D), however, the negative effect size for *Chenopodium album* which was prominent in the across-region effect sizes (Fig. 1) was only present in the Konstanz transplant region, and for both treatments. These plots also revealed significant negative reproductive biomass effect sizes for the non-native *Amaranthus retroflexus* in both treatments of the Konstanz transplant region, for the non-native *E. annuus* in the control treatment of the Potsdam transplant region, and for the non-native *V. persica* in the control treatment of the Konstanz transplant region (Fig. S4D). There was, however, a significant positive reproductive biomass effect sizes for the native *Plantago major* in the control treatment of the Konstanz transplant region (Fig. S4D). For the native *S. nigrum*, the reproductive biomass effect size was negative, and significantly so in the zebularine treatment in the Konstanz transplant region, whereas they were significantly positive in the Potsdam transplant region (Fig. S4D).

**References**

Albert, A., & Anderson, J. A. (1984). On the existence of maximum likelihood estimates in logistic regression models. *Biometrika*, *71*(1), 1–10.

Ascherson, P. (1859). Die wichtigeren im Jahre 1859 entdeckten und bekannt gewordenen Fundorte in der Flora des Vereins­gebiets. *Verh. Bot. Ver. Prov. Brandenburg*, *1*, 1–29.

Bates, D., Mächler, M., Bolker, B., & Walker, S. (2015). Fitting linear mixed-effects models using lme4. *Journal of Statistical Software*, *67*(1), 1–48. doi:10.18637/jss.v067.i01

Costea, M., Weaver, S. E., & Tardif, F. J. (2011). The biology of Canadian weeds. 130. *Amaranthus retroflexus* L., *A. powellii* S. Watson and *A. hybridus* L. . *Canadian Journal of Plant Science*, *84*(2), 631–668.

Fischer, M. A. (1987). On the origin of *Veronica persica* (Scrophulariaceae)-a contribution to the history of a neophytic weed. *Plant Systematics and Evolution*, *155*(1–4), 105–132.

Frey, D. (2003). *Patterns of variation within the* Erigeron annuus *complex in the United States and Europe*. ETH Zürich. doi:/10.3929/ethz-a-004779416

Friedrich, J. O., Adhikari, N. K. J., & Beyene, J. (2007). Inclusion of zero total event trials in meta-analyses maintains analytic consistency and incorporates all available data. *BMC Medical Research Methodology*, *7*, 1–6. doi:10.1186/1471-2288-7-5

Hadfield, J. D. (2010). MCMC methods for multi-response generalized linear mixed models: The MCMCglmm R package. *Journal of Statistical Software*, *33*(2), 1–22. doi:10.18637/jss.v033.i02

Heinze, J., Gensch, S., Weber, E., & Joshi, J. (2016). Soil temperature modifies effects of soil biota on plant growth. *Journal of Plant Ecology*, *10*(5), 808–821.

Kneis, D., Knoesche, R., & Bronstert., A. (2006). Analysis and simulation of nutrient retention and manage ment for a lowland river-lake system. *Hydrology and Earth System Sciences Discussions*, *10*, 575– 588.

Kühn, I., Durka, W., & Klotz, S. (2004). BiolFlor : A new plant-trait database as a tool for plant invasion ecology. *Diversity & Distributions*, *10*(5), 363–365.

Olsen, S. R., Cole, C. V., Watanabe, F. S., & Dean, L. A. (1954). Estimation of available phosphorus in soils by extraction with sodium bicarbonate. In *Circular, Vol 939* (pp. 1–19). Washington, DC: US Department of Agriculture.

Rainey, C. (2016). Dealing with separation in logistic regression models. *Political Analysis*, *24*(3), 339–355.

Sauter, R., & Held, L. (2016). Quasi-complete separation in random effects of binary response mixed models. *Journal of Statistical Computation and Simulation*, *86*(14), 2781–2796.

Sebald, O., Philippi, G., & Seybold, S. (1993). *Die Farn- und Blütenpflanzen Baden-Württembergs, Bd.1, Allgemeiner Teil; Spezieller Teil (Pteridophyta, Spermatophyta)* (2nd ed.). Stuttgart: Verlag Eugen Ulmer.

Sebald, O., Phillippi, G., Seybold, S., & Wörz, A. (1996). *Die Farn- und Blütenpflanzen Baden-Württembergs, Bd.6, Spezieller Teil (Spermatophyta, Unterklasse Asteridae)*. Stuttgart: Verlag Eugen Ulmer.

Sebald, O., Seybold, S., Philippi, G., Kleinsteuber, A., & Lange, D. (1996). *Die Farn- und Blütenpflanzen Baden-Württembergs, Bd.5 : Spezieller Teil (Spermatophyta, Unterklasse Asteridae)*. Stuttgart: Ulmer (Eugen).

Sweeting, M. J., Sutton, A. J., & Lambert, P. C. (2004). What to add to nothing? Use and avoidance of continuity corrections in meta-analysis of sparse data. *Statistics in Medicine*, *23*(9), 1351–1375.

Viechtbauer, W. (2010). Conducting meta-analyses in R with the metafor Package. *Journal of Statistical Software*, *36*(3), 1–48. doi:10.1103/PhysRevB.91.121108

Weaver, S. E. (2011). The biology of Canadian weeds. 115. *Conyza canadensis* . *Canadian Journal of Plant Science*, *81*(4), 867–875.

Weaver, S. E., & Warwick, S. L. (1984). The biology of canadian weeds. 64. *Datura stramonium* L. *Canadian Journal of Plant Science*, *64*, 979–991.

Willdenow, C. L. (1787). *Florae Berolinensis Prodromus*. Berlin.
